# Supplementary material for: Microenvironment-responsive magnesium stents for targeted atherosclerotic plaque removal and restenosis mitigation
Source: Cell Rep Med. 2026 Aug 6;7(8):102980. doi: 10.1016/j.xcrm.2026.102980 (PMC13522762; doi:10.1016/j.xcrm.2026.102980)
Supplement: Supplementary file 1 [file mmc1.pdf]

Cell Reports Medicine, Volume *7*

Supplemental information

Microenvironment-responsive magnesium stents for targeted atherosclerotic plaque removal and restenosis mitigation

Lin Shen, Yi Zhong, Yanran Bi, Ziyu Pang, Youyu Wei, Zhangyu Yang, Xingru Ding, Chengli Jiang, Junchao Yu, Zijian Fang, Mengqian Zhai, Jinhong Sun, Junyan Li, Jieli Zhu, Zhongwei Zhao, Gaofeng Shu, Chenying Lu, Lingchun Lv, Jingjing Song, Minjiang Chen, Xiaokun Li, Zhouguang Wang, Weiqian Chen, and Jiansong Ji

Supplementary Information

Supplemental Figures

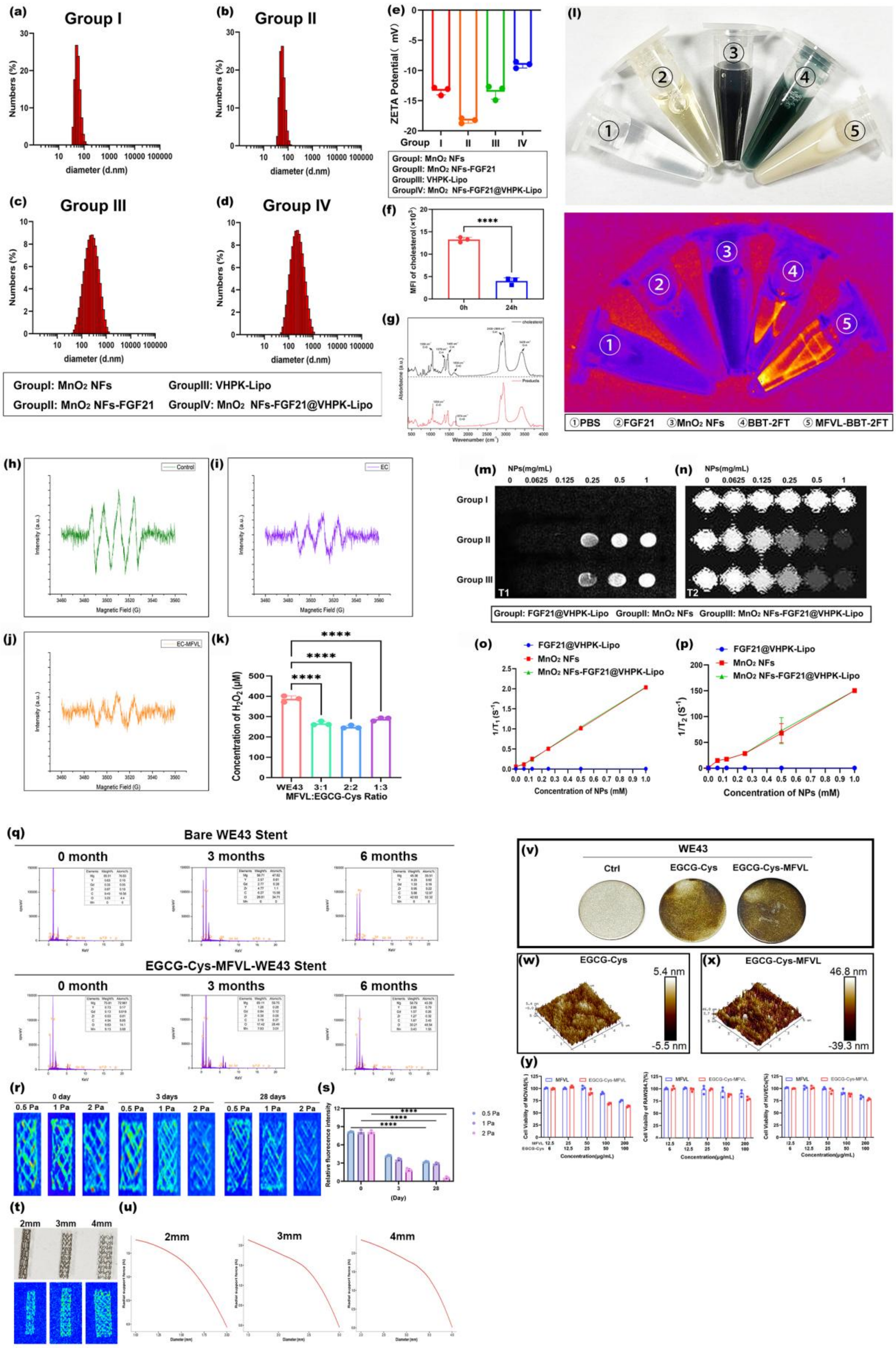

Fig. S1.Characterization of nanoparticle formulations, ROS scavenging capacity, MRI contrast properties, stent coating performance, and cytocompatibility (related to Fig. 1). (a) Group I: MnO_2

NFs, **(b)** Group II: MnO_2 NF-FGF21, **(c)** Group III: VHPK-Lipo, and **(d)** Group IV: MFVL. **(e)** Zeta potential measurements of each nanoparticle formulation. Group I: MnO_2 NFs, Group II: MnO_2 NF-FGF21, Group III: VHPK-Lipo, and Group IV: MFVL. **(f)** Quantitative bar graph analysis corresponding to Fig. 1b. **(g)** FTIR spectra of cholesterol (black) and its enzymatic oxidation products (red). **(h-j)** ESR spectra of different sample groups—(h) Ctrl, (i) EC-coated, and (j) EGCG-Cys-MFVL-coated substrates—used to evaluate the scavenging capacity of ROS, including •OH and/or superoxide radical (O_2•⁻). **(k)** Determination of H_2O_2 scavenging efficiency of nanocoatings with varying MFVL:EGCG-Cys mass ratios (3:1, 2:2, and 1:3) using the HRP-catalyzed ABTS assay. **(l)** Visible light (top) and NIR-II fluorescence (bottom) images of PBS, FGF21, MnO_2 NFs, BBT-2FT, and MFVL-BBT-2FT. **(m, n)** T_1-weighted (m) and T_2-weighted (n) MRI images of FGF21@VHPK-Lipo (Group I), MnO_2 NFs (Group II), and MFVL (Group III) at varying concentrations (0~1 mg/mL). **(o, p)** Plots of $1/T_1$ (o) and $1/T_2$ (p) relaxation rates as a function of nanoparticle concentration for each group. **(q)** Time-dependent EDS spectra of bare and EGCG-Cys-MFVL coated WE43 stents during degradation (0, 3, and 6 months). **(r)** Fluorescence imaging of EGCG-Cys-MFVL coated stents under different shear stress conditions (0.5, 1, and 2 Pa) at indicated time points (0, 3, and 28 days). **(s)** Quantification of fluorescence intensity under different shear stress conditions corresponding to (r). **(t)** Representative optical images and corresponding fluorescence distribution maps of EGCG-Cys-MFVL coated WE43 stents with different diameters (2, 3, and 4 mm). **(u)** Radial force–diameter curves of EGCG-Cys-MFVL coated WE43 stents with different diameters (2, 3, and 4 mm). **(v)** Images of WE43 alloy discs with different surface modifications: uncoated (Ctrl) and coated with EGCG-Cys or EGCG-Cys-MFVL. **(w, x)** AFM images of EC (w) and EGCG-Cys-MFVL (x) nanocoatings, showing differences in nanoscale surface roughness. **(y)** Cell viability of MOVAS VSMCs, RAW264.7 macrophages, and HUVECs after incubation with varying concentrations of MFVL and EGCG-Cys-MFVL formulations, evaluated using the CCK-8 assay to assess cytocompatibility. Data are shown as mean ± SD. Statistical significance was calculated by one-way ANOVA with Tukey's test. Significance: *, $P < 0.05$; **, $P < 0.01$; ***, $P < 0.001$; ****, $P < 0.0001$.

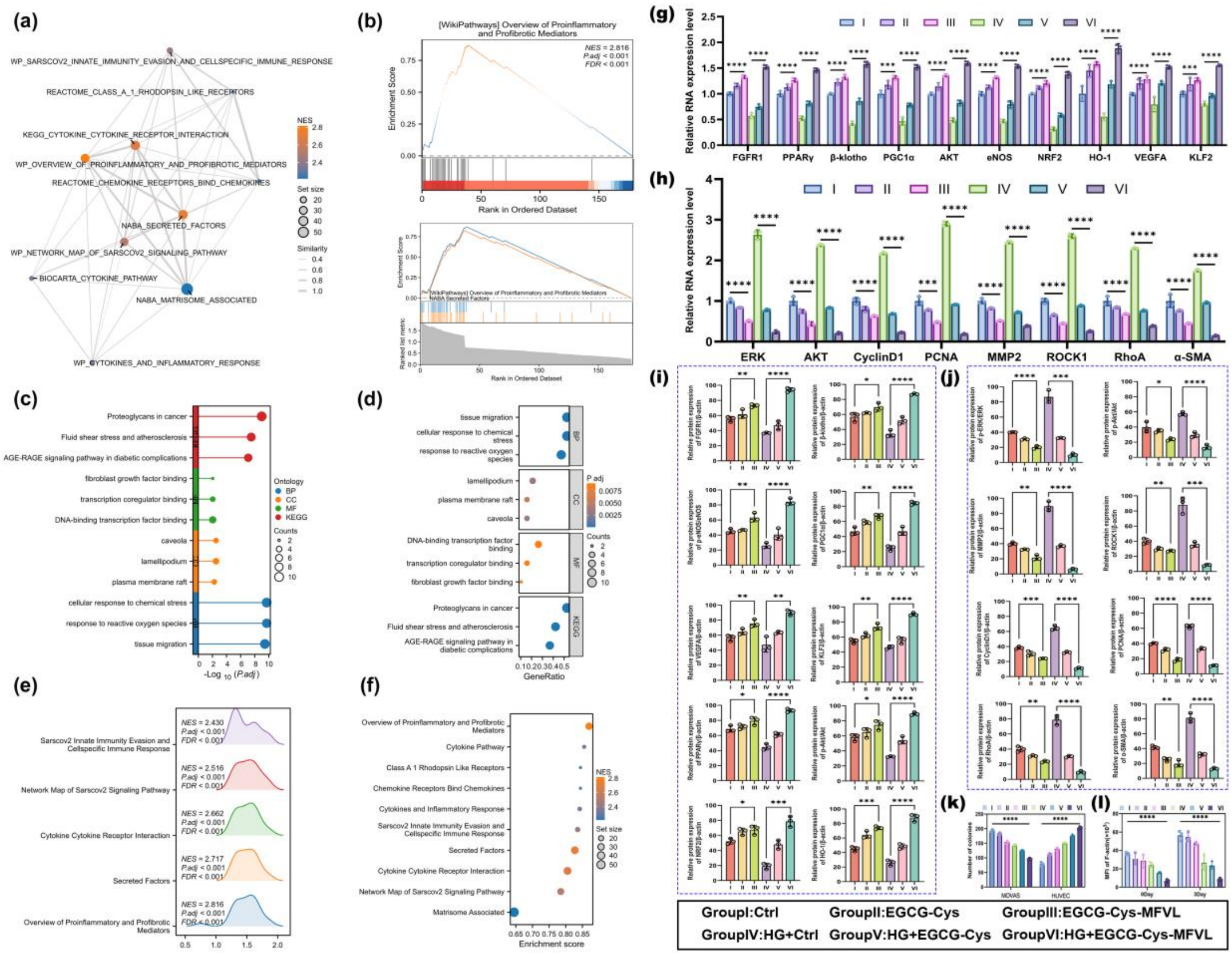

Fig. S2.Transcriptomic pathway enrichment analysis and validation of endothelial and smooth muscle cell functional gene expression under ISR-like pathological conditions (related to Fig. 2). **(a)** Network visualization of enriched pathways based on transcriptomic profiling, highlighting interactions among cytokine signaling, chemokine activity, and extracellular matrix–related processes. **(b)** Gene set enrichment analysis (GSEA) plots of representative pathways associated with proinflammatory and profibrotic mediators. **(c)** Functional enrichment analysis of differentially expressed genes based on Gene Ontology (GO) and KEGG pathways. **(d)** Bubble plot showing enriched GO terms (BP, CC, MF) and KEGG pathways ranked by gene ratio and adjusted *P* value. **(e)** GSEA enrichment plots of selected pathways involved in immune regulation and inflammatory signaling. **(f)** Summary of enriched pathways with corresponding normalized enrichment scores (NES) and gene set sizes. The data were derived from transcriptomic profiling under ISR-like pathological conditions. Statistical significance was determined based on adjusted *P* value and false discovery rate (FDR). **(g)** Relative mRNA expression levels of endothelial function–related genes, including FGFR1, PPARγ, β-klotho, PGC1α, AKT, eNOS, NRF2, HO-1, VEGFA, and KLF2. **(h)** Relative mRNA expression levels of genes associated with smooth muscle cell proliferation and migration, including ERK, AKT, Cyclin D1, PCNA, MMP2, ROCK1, RhoA, and α-SMA. **(i)** Quantification of Western blot analysis corresponding to endothelial function–related signaling pathways in HUVECs shown in Fig. 2e. **(j)** Quantification of Western blot analysis corresponding to proliferation- and migration-related signaling pathways in MOVAS cells shown in Fig. 2d. **(k)** Quantification of colony formation in MOVAS and HUVECs. **(l)** Quantification of F-actin fluorescence intensity at indicated time points.Group I: Ctrl; Group II: EGCG-Cys; Group III: EGCG-Cys-MFVL; Group IV: Ctrl + HG; Group V: EGCG-Cys + HG; Group VI: EGCG-Cys-MFVL + HG. HG, high-glucose condition mimicking a pathological ISR-like microenvironment. Data are shown as mean ± SD. Statistical significance was calculated by one-way ANOVA with Tukey's test. Significance: *, $P < 0.05$; **, $P <$

0.01; ***, $P < 0.001$; ****, $P < 0.0001$.

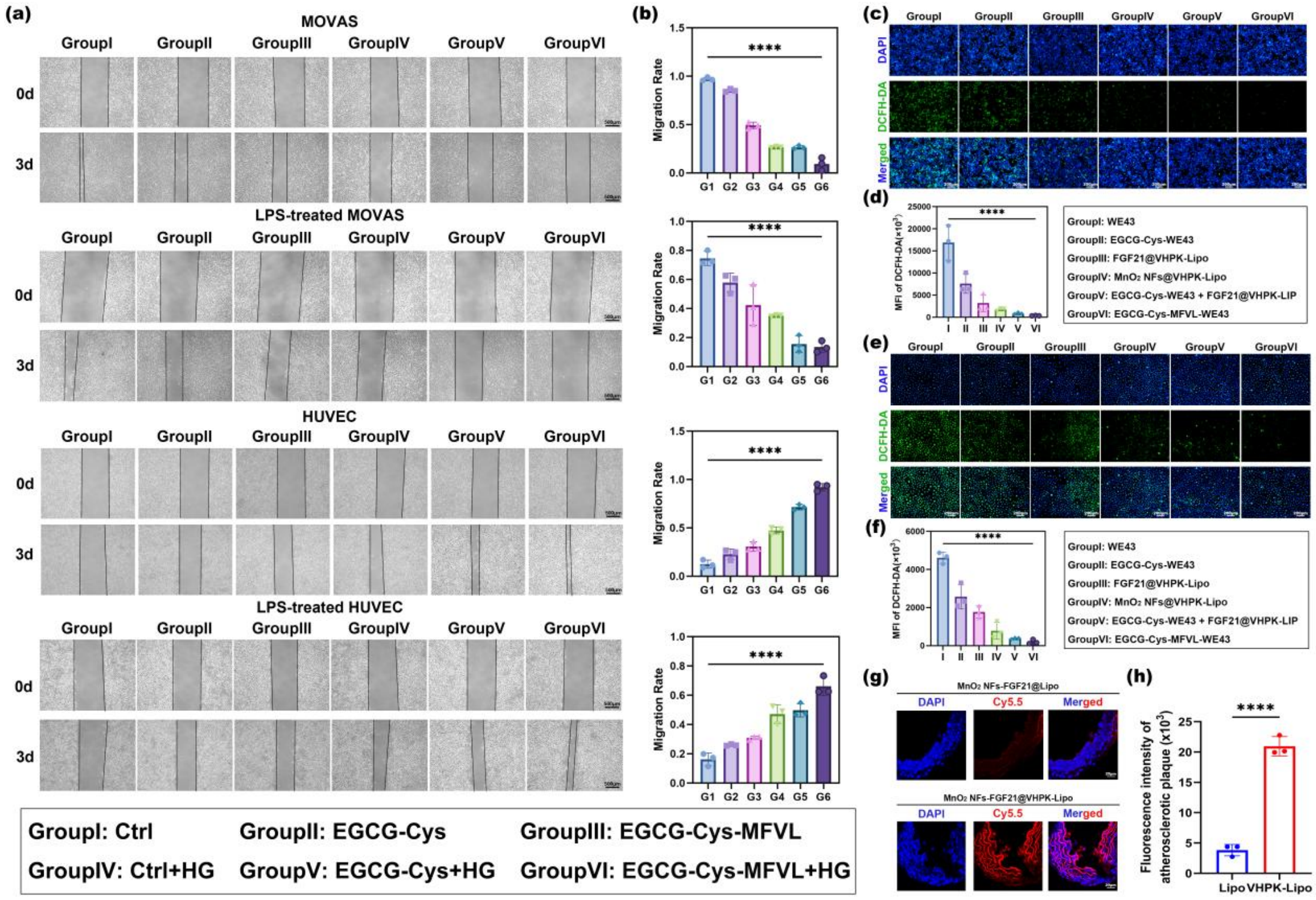

Fig. S3.Evaluation of cell migration, intracellular ROS scavenging in foam cells, and plaque targeting of nanoparticle formulations under inflammatory and metabolic stress conditions (related to Fig. 2-4). (a) Wound healing assay of MOVAS cells and HUVECs under normal and LPS-treated conditions across different treatment groups at 0 and 3 days. Scale bars, 500 μm. **(b)** Quantification of migration rates corresponding to panel (a). Group I: Ctrl; Group II: EGCG-Cys; Group III: EGCG-Cys-MFVL; Group IV: Ctrl + HG; Group V: EGCG-Cys + HG; Group VI: EGCG-Cys-MFVL + HG. LPS, lipopolysaccharide used to induce an inflammatory ISR-like microenvironment; HG, high-glucose condition mimicking metabolic pathological conditions. **(c)** Representative fluorescence images of intracellular ROS levels detected by DCFH-DA staining in macrophage-derived foam cells across different treatment groups. Nuclei are stained with DAPI (blue), and ROS signals are shown in green. Scale bars, 200 μm. **(d)** Quantification of mean fluorescence intensity (MFI) of DCFH-DA corresponding to panel (c). Group I: WE43; Group II: EGCG-Cys-WE43; Group III: FGF21@VHPK-Lipo; Group IV: MnO_2 NFs@VHPK-Lipo; Group V: EGCG-Cys-WE43 + FGF21@VHPK-Lipo; Group VI: EGCG-Cys-MFVL-WE43. **(e)** Representative fluorescence images of intracellular ROS levels detected by DCFH-DA staining in smooth muscle cell–derived foam cells across different treatment groups. Nuclei are stained with DAPI (blue), and ROS signals are shown in green. Scale bars, 200 μm. **(f)** Quantification of mean fluorescence intensity (MFI) of DCFH-DA corresponding to panel (e). Group I: WE43; Group II: EGCG-Cys-WE43; Group III: FGF21@VHPK-Lipo; Group IV: MnO_2 NFs@VHPK-Lipo; Group V: EGCG-Cys-WE43 + FGF21@VHPK-Lipo; Group VI: EGCG-Cys-MFVL-WE43. **(g)** Representative fluorescence microscopy images of atherosclerotic plaques from each group showing DAPI (blue) and Cy5.5-labeled Lipo or MFVL (red) signals. Scale bars, 20 μm. **(h)** Quantitative analysis of Cy5.5 fluorescence intensity in plaques from Lipo and MFVL groups. Data are shown as mean ± SD.

Statistical significance was calculated by one-way ANOVA with Tukey's test. Significance: *, $P < 0.05$; **, $P < 0.01$; ***, $P < 0.001$; ****, $P < 0.0001$.

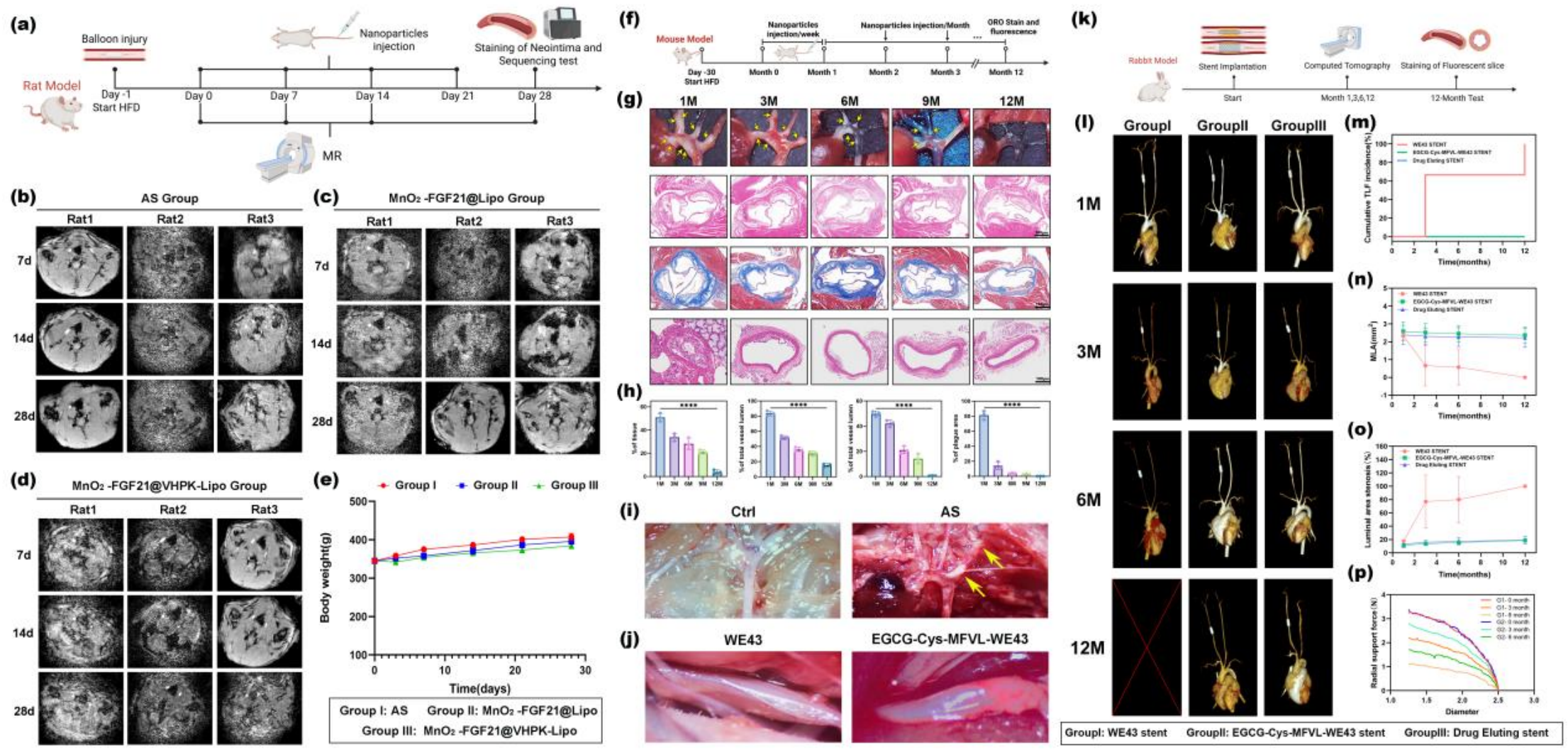

Fig. S4.Longitudinal *in vivo* evaluation of nanoparticle treatment and stent performance in rat, mouse, and rabbit models of atherosclerosis and vascular injury (related to Fig. 5, 6). (a) Schematic timeline of the rat model experiment. Following balloon-induced vascular injury and initiation of a high-fat diet, the rats received nanoparticle treatment on day 7, followed by serial MRI scans at days 7, 14, and 28. Neointimal tissue was harvested on day 28 for histological staining and sequencing analysis. **(b-d)** Representative MRI images of aortic roots from rats in Group I (AS), Group II (MnO_2-FGF21@Lipo), and Group III (MFVL) at 7, 14, and 28 days post-treatment. **(e)** Body weight changes of rats over 28 days in all groups. **(f)** Schematic illustration of the experimental design for long-term treatment in *Apoe*$^{-/-}$ mice, including high-fat diet (HFD) induction and periodic nanoparticle administration. **(g)** Representative stereomicroscopic images of aortic roots at different time points (1, 3, 6, 9, and 12 months) following MFVL treatment. Yellow arrows indicate atherosclerotic lesions. Hematoxylin and eosin (H&E) staining of aortic root sections at indicated time points. Masson's trichrome staining of aortic root sections for evaluation of collagen deposition and plaque remodeling. Histological staining of vascular sections showing morphological changes during long-term treatment. Scale bars, 100 μm. **(h)** Quantitative analysis of plaque burden, including percentage of lesion area, vessel lumen area, and plaque area at different time points. **(i)** Stereomicroscopic observation of aortic arches in the Ctrl (C57BL/6J) and AS (*Apoe*$^{-/-}$ + high-fat diet) mouse models. **(j)** Stereomicroscopic images of carotid arteries in AS rabbits implanted with WE43 or EGCG-Cys-MFVL-WE43 stents. **(k)** Schematic illustration of the experimental design for long-term evaluation in a rabbit stenting model, including stent implantation, serial computed tomography (CT) imaging, and histological analysis up to 12 months. **(l)** Representative three-dimensional CT reconstructions of stented vessels at 1, 3, 6, and 12 months post-implantation in Group I (bare WE43 stent), Group II (EGCG-Cys-MFVL-WE43 stent), and Group III (drug-eluting stent). **(m)** Cumulative target lesion failure (TLF) incidence over time across different treatment groups. **(n)** Longitudinal quantification of minimal lumen area (MLA) derived from CT imaging. **(o)** Luminal area stenosis (%) over time in different groups. **(p)** Radial support force–diameter curves of stents at different time points, reflecting the evolution of mechanical integrity during degradation. Data are shown as mean ± SD. Statistical significance was calculated by

one-way ANOVA with Tukey's test. Significance: *, $P < 0.05$; **, $P < 0.01$; ***, $P < 0.001$; ****, $P < 0.0001$.

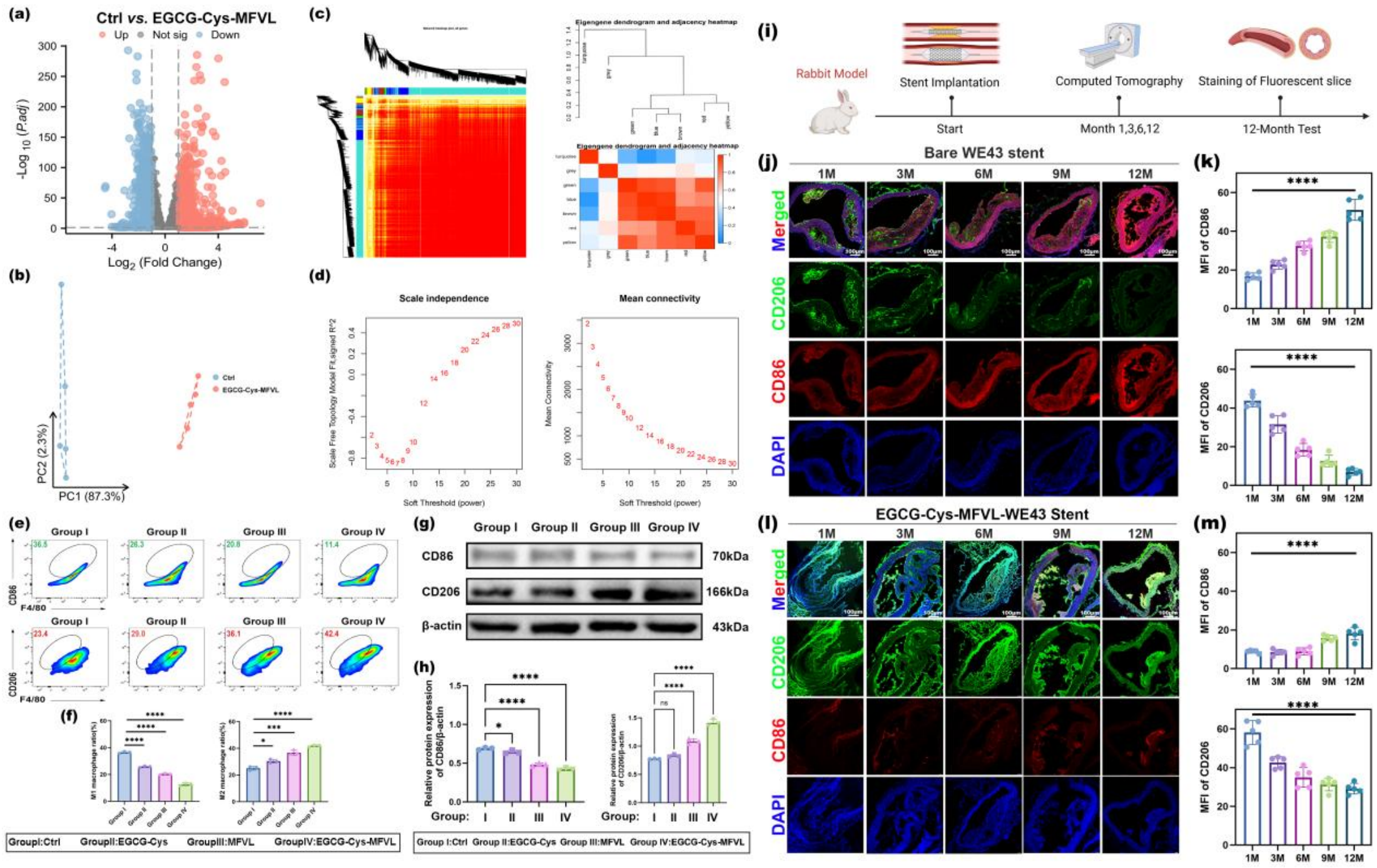

Fig. S5.Transcriptomic profiling, WGCNA, and long-term macrophage polarization analysis following EGCG-Cys-MFVL-coated stent implantation (related to Fig. 7). (a) Volcano plot of genes differentially expressed between EGCG-Cys-MFVL-coated stents and bare WE43 stents, generated based on $\log_2$ fold change ($\log_2$FC) and adjusted *p*-values. Red and blue dots represent genes with significant upregulation and downregulation, respectively ($P < 0.05$, $|\log_2FC| > 1$). **(b)** Principal component analysis (PCA) of transcriptomic profiles from the Ctrl and EGCG-Cys-MFVL groups, with dimensionality reduction plotted across the first two principal components. **(c)** WGCNA of transcriptomic data obtained from rat carotid arteries 1 month after implantation of EGCG-Cys-MFVL-coated and bare WE43 stents. A topological overlap matrix (TOM) was constructed, and gene modules were identified using hierarchical clustering with dynamic tree cutting. Module eigengene relationships were visualized through dendrograms and adjacency heatmaps. **(d)** Soft-thresholding power (β) was determined based on the scale-free topology criterion. The relationship between soft threshold and scale independence (left) and mean connectivity (right) was assessed to ensure optimal network construction. Flow cytometry analysis of aortic single-cell suspensions was performed 1 month after stent implantation to assess macrophage polarization. **(e)** Cells were stained with F4/80 and CD86 (M1) or CD206 (M2) antibodies. Representative density plots are shown for CD86 and CD206. **(f)** Quantification of M1 and M2 macrophage ratios. Group I: bare stents (Ctrl), Group II: EC-coated stents, Group III: MFVL-coated stents, and Group IV: EGCG-Cys-MFVL-coated stents. **(g)** Representative immunoblots. **(h)** Quantitative analysis of relative protein expression for CD86 and CD206. Group I: bare stents (Ctrl), Group II: EC-coated stents, Group III: MFVL-coated stents, and Group IV: EGCG-Cys-MFVL-coated stents. **(i)** Schematic illustration of the long-term experimental design, including stent implantation and serial histological assessment at 1, 3, 6, 9, and 12 months. **(j)** Representative immunofluorescence staining of aortic sections from rabbits implanted with bare WE43 stents, showing CD206$^+$ (M2 macrophages, green), CD86$^+$ (M1

macrophages, red), and nuclei (DAPI, blue) at indicated time points. Scale bars, 100 μm. **(k)** Quantitative analysis of mean fluorescence intensity (MFI) of $CD86^+$ and $CD206^+$ in the bare WE43 group over time. **(l)** Representative immunofluorescence staining of aortic sections from rabbits implanted with EGCG-Cys-MFVL-WE43 stents at corresponding time points. Scale bars, 100 μm. **(m)** Quantitative analysis of MFI of $CD86^+$ and $CD206^+$ in the EGCG-Cys-MFVL-WE43 group. Data are shown as mean ± SD. Statistical significance was calculated by one-way ANOVA with Tukey's test. Significance: *, $P < 0.05$; **, $P < 0.01$; ***, $P < 0.001$; ****, $P < 0.0001$.

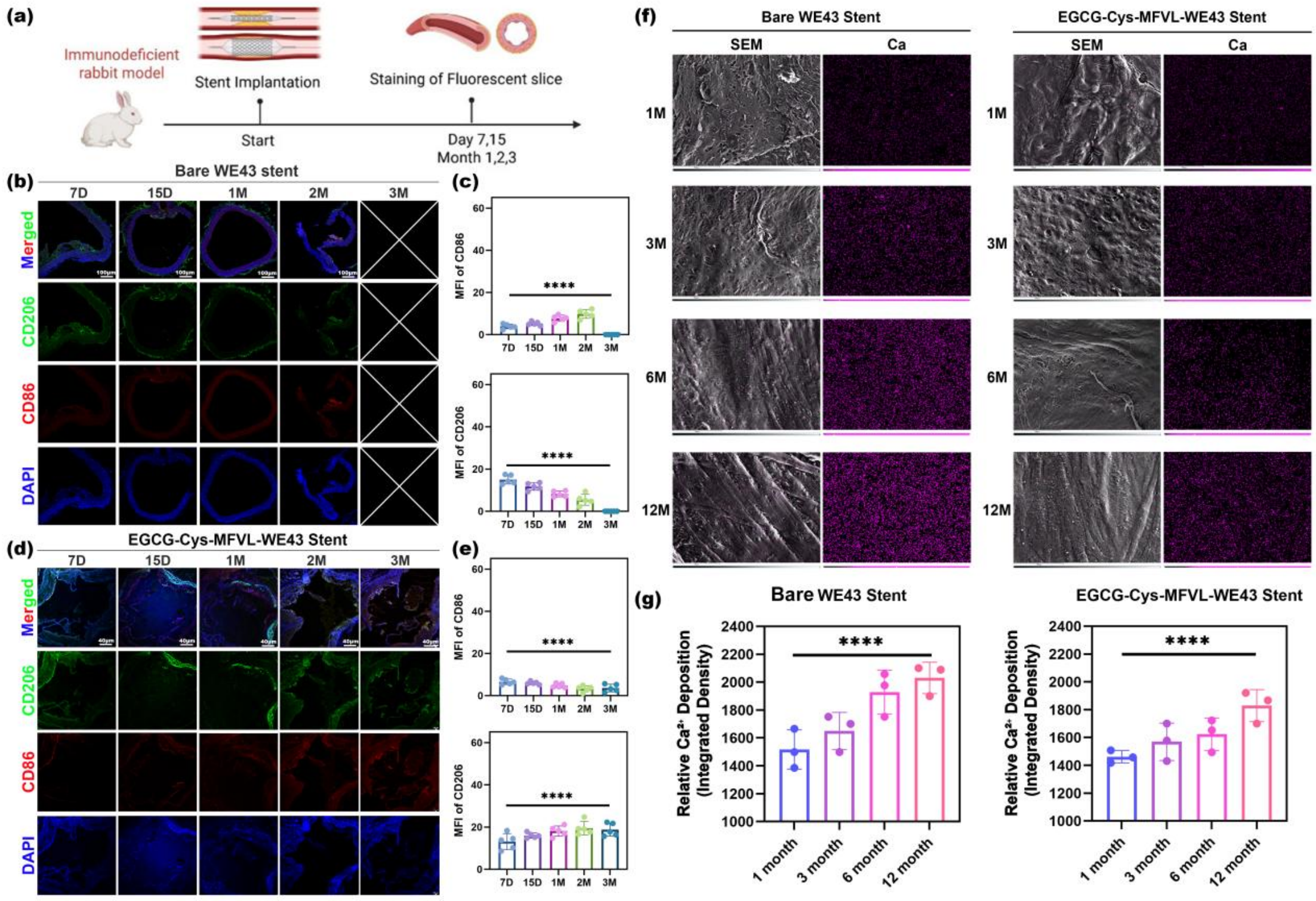

Fig. S6.Time-dependent macrophage polarization and peri-stent calcium deposition analysis in immunodeficient rabbits following bare and EGCG-Cys-MFVL-coated WE43 stent implantation (related to Fig. 7). (a) Schematic illustration of the experimental design in immunodeficient rabbits, including stent implantation and serial histological analysis at 7 days, 15 days, and 1–3 months. **(b)** Representative immunofluorescence staining of vascular sections from rabbits implanted with bare WE43 stents, showing $CD206^+$ (green), $CD86^+$ (red), and nuclei (DAPI, blue) at indicated time points. Scale bars, 100 μm. **(c)** Quantitative analysis of MFI of CD86 and CD206 in the bare WE43 group. **(d)** Representative immunofluorescence staining of vascular sections from rabbits implanted with EGCG-Cys-MFVL-WE43 stents. Scale bars, 100 μm. **(e)** Quantitative analysis of MFI of $CD86^+$ and $CD206^+$ in the EGCG-Cys-MFVL-WE43 group. **(f)** Representative scanning electron microscopy (SEM) images and corresponding Ca elemental mapping of peri-stent vascular surfaces adjacent to bare WE43 and EGCG-Cys-MFVL-coated WE43 stents at indicated time points (1, 3, 6, and 12 months) after implantation. **(g)** Quantitative analysis of Ca^{2+} deposition based on integrated Ca signal intensity corresponding to (f). Data are shown as mean ± SD. Statistical significance was calculated by one-way ANOVA with Tukey's test. Significance: *, $P < 0.05$; **, $P < 0.01$; ***, $P < 0.001$; ****, $P < 0.0001$.

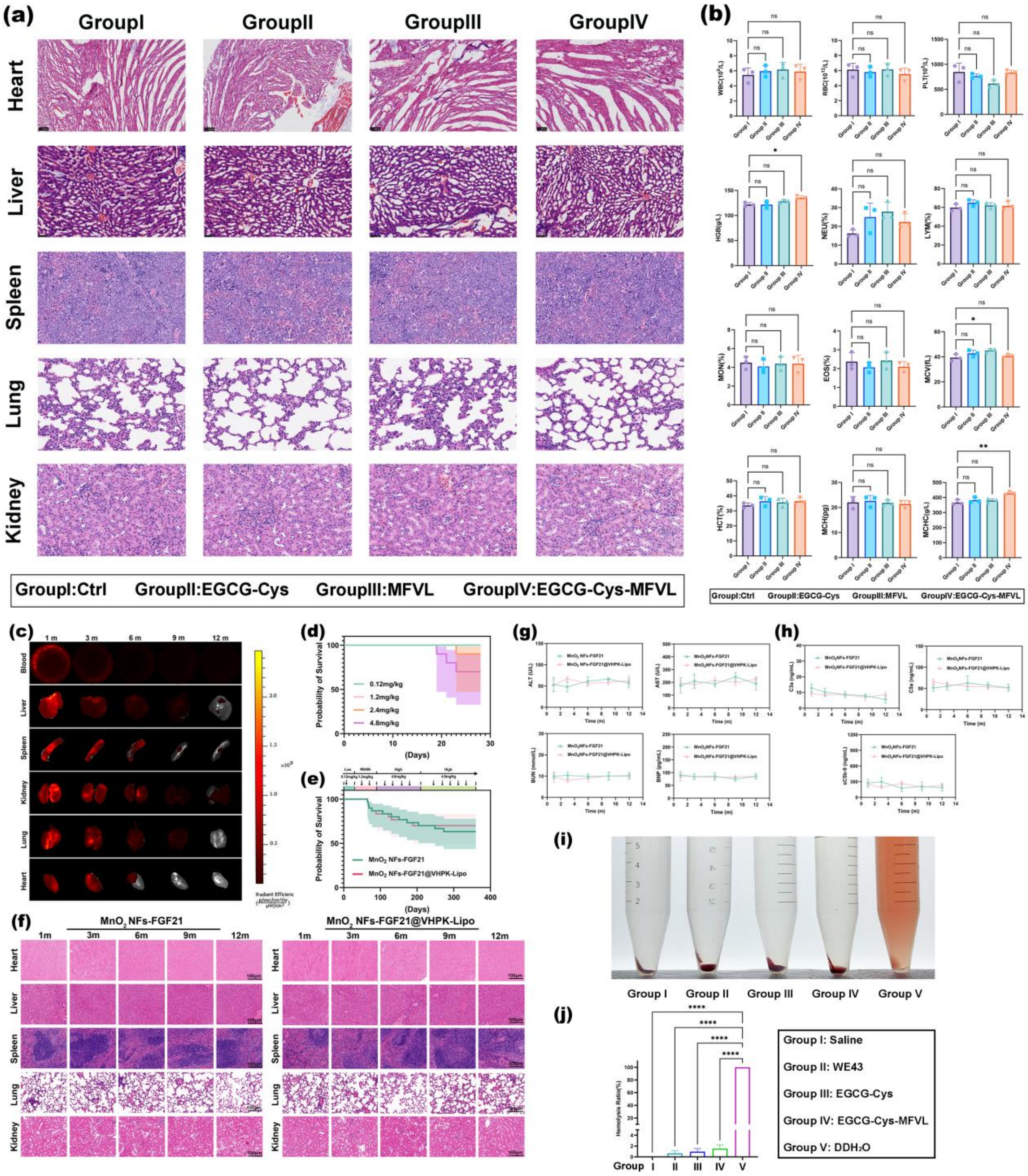

Fig. S7.*In vivo* biocompatibility, biodistribution, long-term safety, and hemocompatibility assessment of nanoparticle formulations and coated stents across multiple organ systems and hematological parameters (related to Fig. 7). (a) Major organs, including the heart, liver, spleen, lung, and kidney, were stained with H&E 1 month after stent implantation to evaluate systemic biocompatibility and potential off-target toxicity. Group I: bare stents (Ctrl), Group II: EC-coated stents, Group III: MFVL-coated stents, and Group IV: EGCG-Cys-MFVL-coated stents. Scale bars, 100 µm. **(b)** Comprehensive hematological analysis was performed 1 month after stent implantation to evaluate systemic hematotoxicity: WBC, RBC, PLT, hemoglobin, NEU%, LYM%, MON%, EOS%, MCV, HCT, MCH, and MCHC. Group I: bare stents (Ctrl), Group II: EC-coated stents, Group III: MFVL-coated stents, and Group IV: EGCG-Cys-MFVL-coated stents. **(c)** *Ex vivo* near-infrared I (NIR-I) fluorescence imaging was performed to assess the tissue distribution of Cy5.5-labeled MnO_2 NFs-FGF21 and MnO_2 NFs-FGF21@VHPK-Liposomes (0.12 mg/kg, monthly). At 1, 3, 6, 9, and 12 months post-administration, blood and major organs (heart, liver, spleen, lung, and kidney) were collected and

imaged under identical acquisition settings. Radiant efficiency was recorded for quantitative analysis. **(d)** Kaplan–Meier survival curves of mice intravenously injected with MnO_2 NFs-FGF21@VHPK-Liposomes at 0.12, 1.2, 2.4, or 4.8 mg/kg and monitored for 1 month. **(e)** Long-term survival assessment of mice treated with MnO_2 NFs-FGF21 or MnO_2 NFs-FGF21@VHPK-Liposomes under a staged monthly dosing regimen: 0.12 mg/kg (month 1), 1.2 mg/kg (months 2–3), and 4.8 mg/kg (months 4–12). All formulations were administered via tail vein injection once per month. Survival status was recorded throughout the study. **(f)** Hematoxylin and eosin (H&E) staining of heart, liver, spleen, lung, and kidney tissues collected at 1, 3, 6, 9, and 12 months after intravenous administration of MnO_2 NFs-FGF21 or MnO_2 NFs-FGF21@VHPK-Liposomes. Tissue sections were prepared and analyzed under identical histological conditions to assess long-term morphological alterations. Scale bars, 100 μm. **(g)** Mice received intravenous MnO_2 NFs-FGF21 or MnO_2 NFs-FGF21@VHPK-Liposomes (0.12 mg/kg, once monthly). Blood samples were collected at 1, 3, 6, 9, and 12 months for serum biochemical assessment, including alanine aminotransferase (ALT), aspartate aminotransferase (AST), blood urea nitrogen (BUN), and brain natriuretic peptide (BNP). **(h)** Under the same dosing regimen (0.12 mg/kg, once monthly), serum samples collected at 1, 3, 6, 9, and 12 months were analyzed for complement activation markers, including C3a, C5a, and soluble C5b-9 (sC5b-9). **(i)** Representative images of the hemolysis assay performed by incubating red blood cells with various test groups: Group I (physiological saline, negative control), Group II (WE43 alloy), Group III (EGCG-Cys nanocoating), Group IV (EGCG-Cys-MFVL nanocoating), and Group V (deionized water, positive control). **(j)** Quantification of hemolysis ratio (%) by spectrophotometric analysis of hemoglobin release from the supernatant, used to evaluate the hemocompatibility of the tested materials. Data are shown as mean ± SD. Statistical significance was calculated by one-way ANOVA with Tukey's test. Significance: *, $P < 0.05$; **, $P < 0.01$; ***, $P < 0.001$; ****, $P < 0.0001$; ns, not significant.
