# Supplementary material for: Microenvironment-responsive magnesium stents for targeted atherosclerotic plaque removal and restenosis mitigation
Source: Cell Rep Med. 2026 Aug 6;7(8):102980. doi: 10.1016/j.xcrm.2026.102980 (PMC13522762; doi:10.1016/j.xcrm.2026.102980)
Supplement: Supplementary file 2 [file mmc3.pdf]

Cell Reports Medicine

Article

Microenvironment-responsive magnesium stents for targeted atherosclerotic plaque removal and restenosis mitigation

Graphical abstract

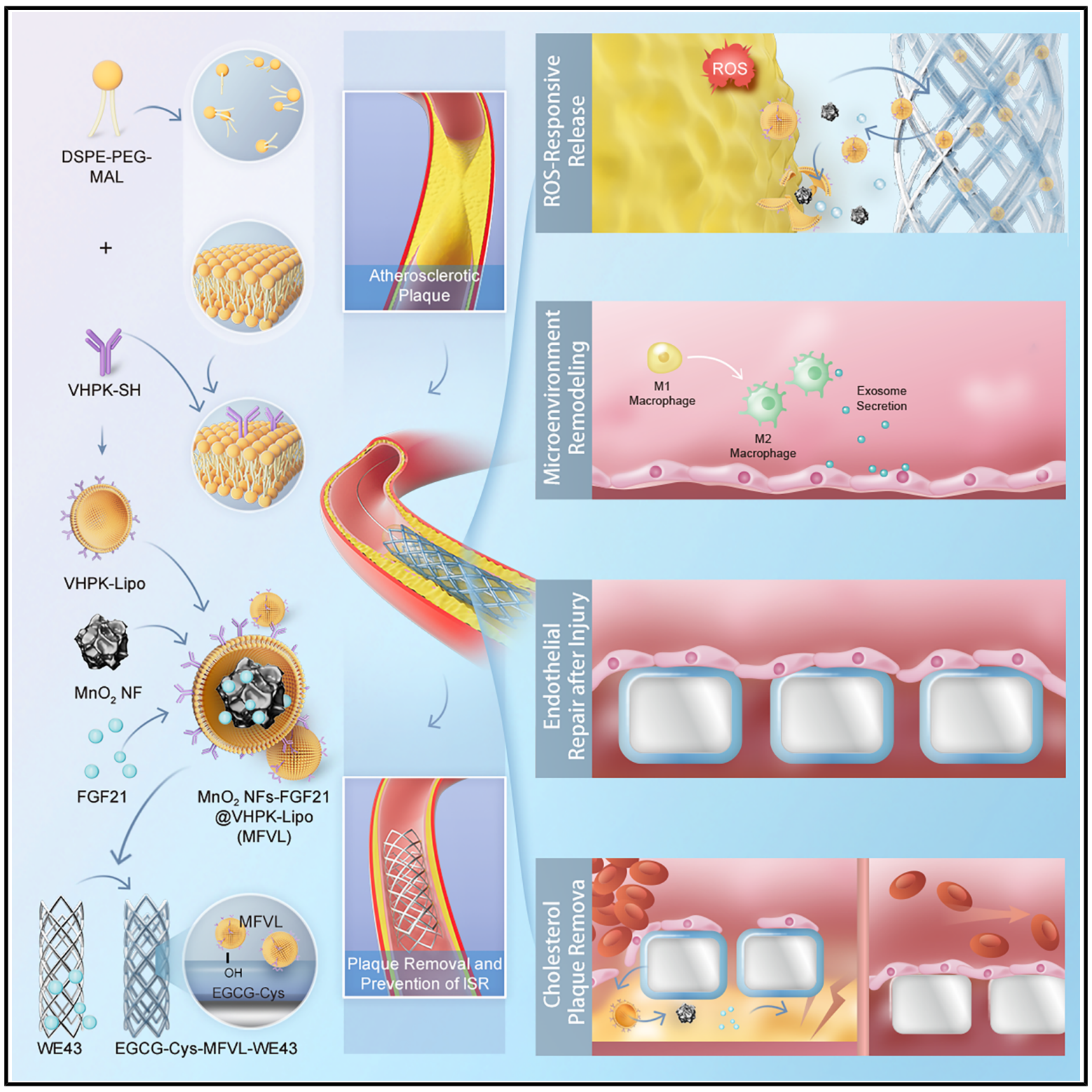

Authors

Lin Shen, Yi Zhong, Yanran Bi, ..., Zhouguang Wang, Weiqian Chen, Jiansong Ji

Correspondence

lixk1964@163.com (X.L.),
wzhouguang@gmail.com (Z.W.),
ls2119088@126.com (W.C.),
jjstcty@wmu.edu.cn (J.J.)

In brief

Shen et al. develop a microenvironment-responsive magnesium stent that integrates nanocoating and liposomal delivery to target atherosclerotic plaques. The system modulates cholesterol metabolism, oxidative stress, and immune responses, enabling coordinated vascular remodeling and reducing restenosis across multiple *in vivo* models.

Highlights

- Microenvironment-responsive Mg stents enable targeted plaque clearance
- Integrated platform modulates cholesterol, ROS, and immune responses
- Coordinated signaling reprograms EC-VSMC-macrophage interactions
- *In vivo* studies show reduced restenosis and sustained vascular patency

Shen et al., 2026, Cell Reports Medicine 7, 102980
August 18, 2026 © 2026 The Authors. Published by Elsevier Inc.
https://doi.org/10.1016/j.xcrm.2026.102980

CellPress

Cell Reports Medicine

CellPress OPEN ACCESS

Article

Microenvironment-responsive magnesium stents for targeted atherosclerotic plaque removal and restenosis mitigation

Lin Shen,[1,4,5,9] Yi Zhong,[1,4,8,9] Yanran Bi,[5,9] Ziyu Pang,[2] Youyu Wei,[1,4,8] Zhangyu Yang,[1,4,8] Xingru Ding,[1,4,8] Chengli Jiang,[1,4,8] Junchao Yu,[1,4,8] Zijian Fang,[1,4,8] Mengqian Zhai,[1,4,8] Jinhong Sun,[1,4,8] Junyan Li,[1,4,8] Jieli Zhu,[1,4,8] Zhongwei Zhao,[1,4] Gaofeng Shu,[1,4,8] Chenying Lu,[1,4,8] Lingchun Lv,[1,3,4] Jingjing Song,[1,4,8] Minjiang Chen,[1,4,8] Xiaokun Li,[5,*] Zhouguang Wang,[6,7,*] Weiqian Chen,[1,2,4,8,*] and Jiansong Ji[1,2,4,8,10,*]

[1]Zhejiang Key Laboratory of Imaging and Interventional Medicine, Zhejiang Engineering Research Center of Interventional Medicine Engineering and Biotechnology, Key Laboratory of Precision Medicine of Lishui City, the Fifth Affiliated Hospital of Wenzhou Medical University, Lishui 323000, China

[2]International Clinical School, Hangzhou Medical College (Lishui Central Hospital), Lishui, Zhejiang, China

[3]Department of Cardiology, the Fifth Affiliated Hospital of Wenzhou Medical University, Lishui 323000, China

[4]Clinical College of The Affiliated Central Hospital, School of Medcine, Lishui University, Lishui 323000, China

[5]School of Pharmaceutical Science, Wenzhou Medical University, Wenzhou 325035, P.R. China

[6]The First Affiliated Hospital of Wenzhou Medical University, Wenzhou, Zhejiang 325035, China

[7]Oujiang Laboratory (Zhejiang Lab for Regenerative Medicine, Vision, and Brain Health), National Key Laboratory of Macromolecular Drugs and Large-scale Preparation, School of Pharmaceutical Sciences, Wenzhou Medical University, Wenzhou, Zhejiang 325035, China

[8]Department of Radiology, Lishui Hospital of Zhejiang University, School of Medicine, Lishui 323000, China

[9]These authors contributed equally

[10]Lead contact

*Correspondence: lixk1964@163.com (X.L.), wzhouguang@gmail.com (Z.W.), ls2119088@126.com (W.C.), jjstcty@wmu.edu.cn (J.J.)

https://doi.org/10.1016/j.xcrm.2026.102980

SUMMARY

Atherosclerotic plaque persistence and maladaptive vascular remodeling drive in-stent restenosis despite effective revascularization. Here, we develop a microenvironment-responsive magnesium stent with an EGCG-Cys-MFVL nanocoating for targeted plaque clearance. The system enables cholesterol oxidation, reactive oxygen species (ROS) scavenging, and targeted delivery, reprogramming macrophage polarization and vascular homeostasis. Mechanistically, it activates PPARγ/PGC1α pathways in endothelial cells while suppressing proliferative signaling in smooth muscle cells. *In vivo*, the stent exhibits enhanced plaque targeting, prolonged retention, and sustained efficacy, reducing neointimal hyperplasia and improving luminal patency across multiple animal models. Long-term evaluation shows favorable biosafety and degradation. These findings establish a device-integrated strategy for coordinated plaque removal and microenvironmental remodeling to mitigate restenosis.

INTRODUCTION

Cardiovascular diseases (CVDs) are the leading cause of death worldwide; coronary artery disease (CAD), especially acute myocardial infarction, is the primary contributor.[1] Atherosclerosis (AS) underpins CVD, a leading cause of global mortality.[2] Percutaneous coronary intervention (PCI) effectively restores blood flow but is limited by in-stent restenosis (ISR).[3] In 2023, the number of PCI procedures in China exceeded 1.4 million, representing a 10% increase from 2022.[4] Stent implantation, which prevents vessel recoil better than balloon angioplasty alone, has become the dominant PCI method. However, ISR remains a significant challenge, compromising long-term PCI outcomes and contributing to the recurrence of CAD.[5] Current strategies remain insufficient, with ISR occurring in >10% of patients.[6] Alarmingly, 25% of ISR cases lead to acute myocardial infarction due to insufficient intervention, often resulting in death.[6] Thus, further research on ISR is needed to improve PCI outcomes in CAD patients.

Magnesium (Mg) stents offer degradability, biocompatibility, and transient vascular support.[7,8] These alloy systems highlight the broader potential applicability of magnesium-based stents as bioresorbable vascular platforms. Among them, the WE43 Mg alloy stent (Mg, yttrium 4.0%, rare earth elements 3.3%, zirconium 0.5%) is an emerging solution to address ISR, offering favorable biocompatibility and mechanical properties.[9,10] As a natural calcium channel blocker, Mg^{2+} not only inhibits thrombosis but also improves AS, ultimately providing cardiovascular protection.[11] A key feature of the WE43 stent is its ability to gradually degrade within the body and be replaced by newly formed tissue, allowing the vessel to return to its natural state, thus suggesting potential utility in vascular intervention.[9] While the WE43

© 2026 The Authors. Published by Elsevier Inc.
This is an open access article under the CC BY license (http://creativecommons.org/licenses/by/4.0/).

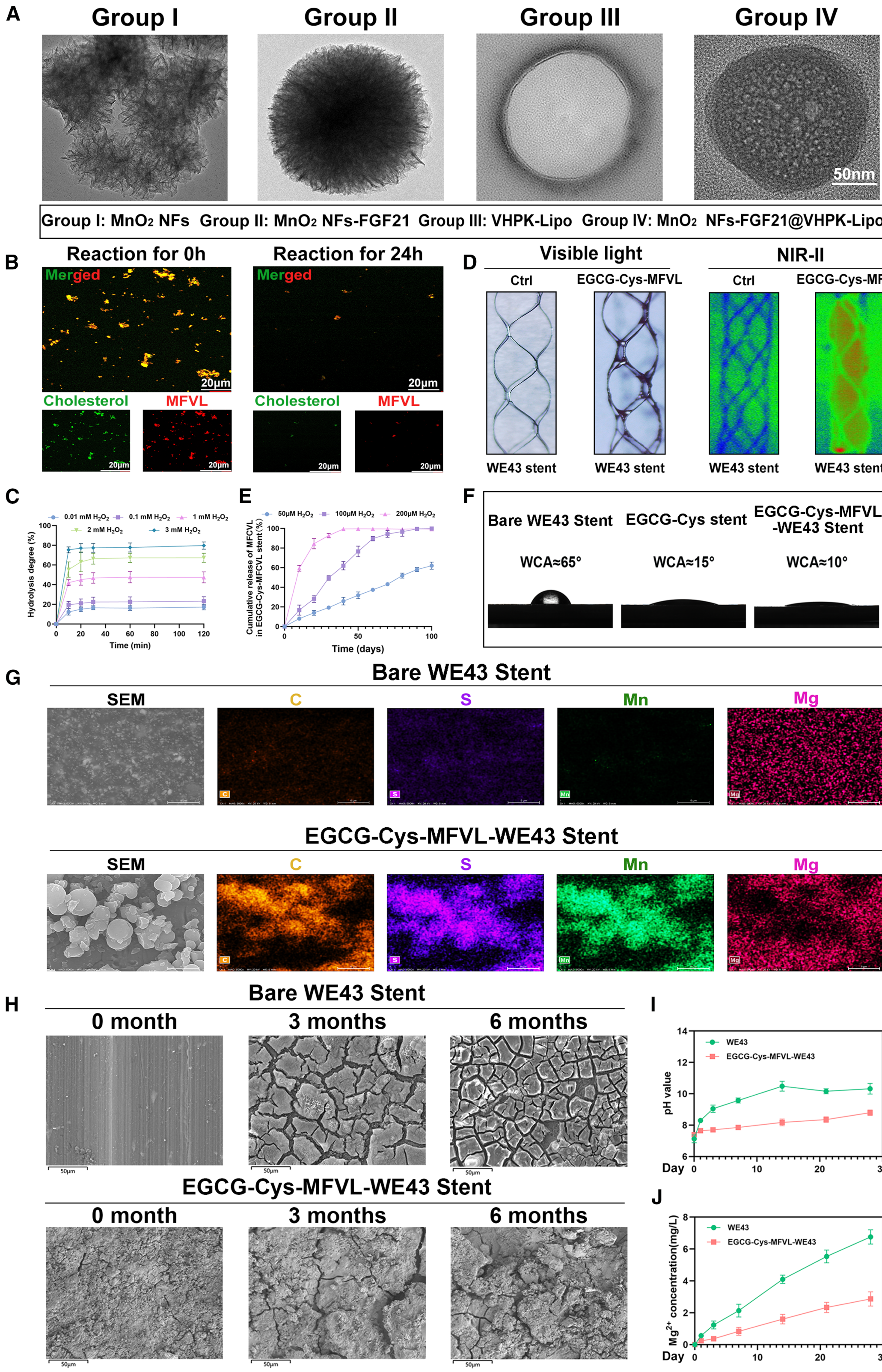

(legend on next page)

stent has improved positive remodeling and eliminated permanent implantation, its use alone still faces challenges including ISR, thrombosis, and inflammatory storms.[9,12]

Emerging evidence suggests that the accumulation of cholesterol crystals following WE43 stent implantation plays a critical role in triggering ISR by creating an inflammatory microenvironment rich in reactive oxygen species (ROS). Traditional stents focus on revascularization but often neglect the burden of atherosclerotic plaques, which, when coupled with stent degradation, can lead to ISR and late stent thrombosis.[13–16] In patients with AS, chronic conditions such as diabetes and hyperlipidemia exacerbate low-density lipoprotein (LDL)-cholesterol accumulation at the stent site, driving ROS production and the polarization of macrophages to the pro-inflammatory M1 phenotype.[17,18] This process forms a dense, inflammatory plaque environment rich in ROS and cholesterol crystals, which are prone to rupture and can lead to neoatherosclerosis following stent implantation.[19–21] Thus, beyond the structural and degradable benefits of WE43 stents, targeted removal of nearby atherosclerotic plaques is essential for mitigating ISR and enhancing the long-term success of cardiovascular interventions. To effectively enhance the targeted clearance of adhered plaques and the remodeling of their microenvironment by the WE43 stent, this study developed a nanodrug-loaded degradable stent. This stent features a crosslinked nanocoating (termed EGCG-Cys) of epigallocatechin gallate (EGCG) and cysteine (Cys), which encapsulates manganese dioxide (MnO_2) nanoflowers (NFs) and fibroblast growth factor 21 (FGF21) within liposomes modified with the VHPKQHRGGSKGC (VHPK) peptide. This design enables responsive interaction with and clearance of the ROS-rich, cholesterol-crystal-laden microenvironment in atherosclerotic plaques, resulting in the EGCG-Cys-MFVL-WE43 stent.

Functioning as nanozymes with peroxidase-like activity, the MnO_2 NFs scavenge ROS and cholesterol crystals, contributing to modulation of the plaque microenvironment within the stent.[22–24] In addition, by activating the nuclear-factor-erythroid-2-related factor 2 (NFE2L2/NRF2) signaling pathway, FGF21 not only repairs endothelial damage but also inhibits endothelial cell apoptosis, further contributing to therapeutic effects of the EGCG-Cys-MFVL-WE43 stent.[25,26] Moreover, the VHPK-peptide-modified liposomes are designed to preferentially bind to inflamed endothelial cells highly expressing vascular cell adhesion molecule 1 (VCAM-1), increasing the targeted delivery and internalization of the therapeutic agents at atherosclerotic sites.[27,28] Furthermore, EGCG, a natural polyphenol, exhibits antioxidant, adhesive, and antiplatelet properties due to its abundant hydroxyl and catechol groups.[29,30] The EGCG-Cys nanocoating, formed through spontaneous cross-linking, is responsive to ROS and releases antioxidant EGCG in oxidative environments, endowing the stent with adaptive ROS-scavenging capabilities.[31,32] Additionally, EGCG promotes the release of nitric oxide by stabilizing and catalyzing S-nitrosoglutathione, which further inhibits platelet adhesion and smooth muscle cell (SMC) proliferation, supporting endothelial repair.[33,34] In summary, the EGCG-Cys-MFVL-WE43 stent enables plaque clearance, endothelial repair, and SMC suppression, reducing ISR and LST while promoting vascular microenvironment remodeling in preclinical models.

RESULTS AND DISCUSSION

Design and characterization of the EGCG-Cys-MFVL-WE43 stent

We fabricated MnO_2 NFs (~96 nm) using a citric acid reduction method. FGF21 was co-loaded onto MnO_2 NFs via amide coupling, and the system was functionalized with the VHPK peptide to yield MFVL (~175 nm, Figures S1A–S1D). Transmission electron microscopy (TEM) revealed NF-like MnO2 structures and core-shell MFVL architecture. Significantly, group IV presented a core-shell architecture, encapsulating MnO_2 NFs within the MFVL liposome, forming a multifunctional design for targeted and therapeutic delivery (Figure 1A). Zeta potential analysis (Figure S1E) showed that MnO_2 NF-FGF21 exhibited the most negative charge (approximately −20 mV), followed by MnO_2 NFs and MFVLs (approximately −15 mV), with VHPK-Lipo being the least negative (approximately −10 mV). To evaluate the cholesterol-clearing efficiency of MFVL nanoparticles, an *in vitro* reaction system was constructed using fluorescein isothiocyanate (FITC)-labeled cholesterol crystals and Cy5.5-labeled MFVL nanoparticles. Confocal imaging confirmed cholesterol association and >70% clearance at 24 h (Figures 1B and S1F). MnO_2 nanozymes exhibit ROS scavenging and cholesterol oxidation (ChOx) capabilities.[35] The NF-like architecture of MnO_2 enhances catalytic surface area and enzymatic efficiency.[35,36]

Fourier transform infrared spectroscopy (FTIR) spectroscopy confirmed the enzymatic oxidation of cholesterol by MFVLs, as evidenced by the emergence of a characteristic carbonyl (C=O) stretch at 1,674 cm^{-1} and the attenuation of the C=C band at 1,636 cm^{-1} (Figure S1G). These spectral shifts, along with the preserved C–O signal, indicate the structural transformation of cholesterol into carbonyl-enriched oxidation products.

Figure 1. Characterization and surface engineering of MnO_2 nanocomposite and WE43 stent system

(A) TEM images of nanostructures: group I (MnO_2 NFs), group II (MnO_2 NFs-FGF21), group III (VHPK-modified liposomes), and group IV (MFVL nanocomposites with core-shell structure). Scale bars, 50 μm.

(B) CLSM imaging of MFVL-mediated cholesterol interaction at 0 and 24 h: green, FITC-cholesterol; red, Cy5.5-MFVL. Scale bars, 20 μm.

(C) Hydrolytic response of MFVL nanoparticles under different H_2O_2 concentrations (0.01, 0.1, 1, 2, and 3 mM).

(D) Visible light and NIR-II fluorescence imaging of EGCG-Cys-MFVL-coated WE43 stents.

(E) Cumulative release profiles of MFVL from EGCG-Cys-MFVL-coated stents under different oxidative conditions.

(F) WCA measurements of bare, EGCG-Cys, and EGCG-Cys-MFVL stent surfaces.

(G) SEM imaging and EDS elemental mapping (C, S, Mn, and Mg) of bare and EGCG-Cys-MFVL-coated stents. Scale bars, 8 μm.

(H) Time-dependent SEM imaging of stent surfaces during degradation (0, 3, and 6 months). Scale bars, 50 μm.

(I) Measurement of pH variation in degradation media over time.

(J) Quantification of Mg^{2+} release during degradation.

The observed peak evolution closely mirrors that reported for ChOx-catalyzed conversion to cholestenone, underscoring the ChOx-mimetic catalytic function of MFVLs.[37] This molecular-level evidence validates the MFVL as a bioinspired nanozyme with lipid-remodeling capability in pathological microenvironments. The MFVL nanoparticles also demonstrated a distinct H_2O_2-concentration-dependent hydrolytic response, with minimal degradation (<25%) under low oxidative stress (0.01–0.1 mM H_2O_2) and rapid breakdown (>75%) within 20 min under high H_2O_2 levels (3 mM), followed by a stable plateau (Figure 1C). This redox-responsive behavior highlights the intrinsic sensitivity of the MFVL system to pathological ROS signals, enabling selective activation within oxidative disease microenvironments. The design rationale is further supported by a growing body of literature on MnO_2-based nanozymes, which have been widely recognized for their ROS-scavenging activity, particularly through catalase-like decomposition of H_2O_2.[38,39] The NF-like morphology of MnO_2 adopted in this study imparts a hierarchically porous structure and enlarged surface area, facilitating catalytic activity and reactant diffusion efficiency.[40,41]

Electron spin resonance (ESR) analysis demonstrated effective scavenging of hydroxyl radicals (·OH) by both the EGCG-Cys and EGCG-Cys-MFVL nanocoatings, with the latter exhibiting enhanced antioxidant performance due to its incorporation of the MFVL. Complementary horseradish peroxidase (HRP)–2,2′-azino-bis(3-ethylbenzothiazoline-6-sulfonate) (ABTS) assays further confirmed that increasing MFVL content markedly enhanced H_2O_2 scavenging efficiency, highlighting a synergistic effect in mitigating oxidative stress through multifunctional surface engineering (Figures S1H–S1K). The reduction in ESR signal reflects the ROS-responsive behavior of the nanocoatings, attributable to the redox-active phenolic groups in EGCG and the synergistic antioxidative function of the MFVL matrix. EGCG provides antioxidant activity and strong interfacial adhesion, enabling ROS-responsive protection and biointerface stabilization.[42,43] This catechol moiety not only facilitates strong adhesion to metallic and polymeric substrates through hydrogen bonding and coordination interactions but also serves as an electron donor in redox reactions.[42,44,45] Upon exposure to ROS, EGCG can scavenge free radicals via electron transfer and hydrogen atom donation, thereby neutralizing ·OH and interrupting oxidative chain propagation.[43,44] Furthermore, EGCG exhibits the ability to chelate transition metal ions, such as iron (Fe^{2+}) and copper (Cu^{2+}), which are critical mediators of Fenton-like reactions in oxidative microenvironments.[29,46] In the context of stent nanocoatings, these multifunctional properties enable EGCG to act as both a redox buffer and a surface anchoring moiety.[29,45,46] The incorporation of EGCG into EGCG-Cys and EGCG-Cys-MFVL nanocoatings imparts intrinsic ROS-responsiveness and reinforces nanocoating-substrate adhesion and stability, while simultaneously providing antioxidative effects at the blood-stent interface. This dual role enables the design of bioinspired smart nanocoatings with enhanced resistance to oxidative stress and vascular compatibility post-implantation.

To further validate the successful fabrication of the nanocoated biodegradable EGCG-Cys-MFVL-WE43 stent and enable *in vivo* visualization of its delivery, the MFVL nanoparticles were surface-functionalized with the NIR-II fluorophore benzo [1, 2-c; 4, 5-c'] bis [1, 2, 5] thiadiazole-4, 7-bis (9, 9-dioctyl-9H-fluoren-2-yl) thiophene (BBT-2FT, Figure S1I). As a small-molecule NIR-II probe, BBT-2FT offers deep tissue penetration and low background noise.[47] Our previous study demonstrated that BBT-2FT can be efficiently nanoformulated and used for real-time tracking of atherosclerotic plaques, making it well suited for integration into multifunctional theranostic platforms.[48] Visible and NIR-II imaging confirmed the successful surface functionalization of the WE43 stents with EGCG-Cys-MFVL nanoparticles. The coated stents exhibited enhanced contrast under visible light and strong NIR-II fluorescence signals, indicating stable nanoparticle adherence and enabling targeted optical tracking (Figure 1D). Additionally, MnO_2 NFs and MFVL exhibited concentration-dependent signal enhancement in both T_1 and T_2 modes, whereas FGF21@VHPK-Lipo alone showed negligible contrast. Quantitative analysis of relaxation rates revealed that the $1/T_1$ and $1/T_2$ values increased linearly with nanoparticle concentration for the MnO_2-containing groups, indicating their dual-mode magnetic resonance imaging (MRI) contrast capability (Figures S1M–S1P). MnO_2 nanostructures can gradually release paramagnetic Mn^{2+} ions under physiological conditions, effectively modulating proton spin relaxation and enhancing both T_1- and T_2-weighted MRI signals.[49] This controlled transformation endows the material with dual-mode imaging performance and establishes its foundation as a responsive MRI contrast agent. MnO_2 nanostructures confer both imaging and therapeutic responsiveness, serving as a pivotal component for multimodal imaging-guided theranostics.

The cumulative release profiles of MFVL- from EGCG-Cys-MFVL-coated stents under varying oxidative conditions revealed a clear H_2O_2-responsive release behavior (Figure 1E). Higher concentrations of H_2O_2 (200 μM) markedly accelerated drug release, achieving nearly 100% within 20 days, while moderate (100 μM) and low (50 μM) H_2O_2 levels resulted in slower and more sustained release kinetics. These results demonstrate that the EGCG-Cys-based nanocoating exhibits redox-sensitive degradation, enabling environment-adaptive drug release in response to oxidative stress, which is advantageous for targeted vascular therapy of inflammatory lesions. The redox-responsiveness of the EGCG-Cys nanocoating arises from disulfide bonds formed via Michael addition and Schiff base crosslinking between EGCG and cystamine.[32,50,51] These dynamic S–S linkages remain inert under physiological conditions but undergo selective cleavage or oxidation in ROS-enriched pathological environments, such as inflamed vasculature.[32,50] This oxidative disruption initiates controlled matrix disassembly, triggering the release of nanoparticles.[32,50] Such redox-triggered degradation enables spatiotemporally targeted, redox-responsive drug delivery. By aligning material degradation with oxidative microenvironment cues, the EGCG-Cys interface offers a redox-responsive platform for next-generation vascular implants.

Surface modification with EGCG-Cys and EGCG-Cys-MFVL nanocoatings significantly reduced the water contact angle (WCA) of WE43 stents from ∼65° to ∼15° and ∼10°, respectively, indicating enhanced hydrophilicity (Figure 1F). Consistent with previous reports highlighting the biocompatibility of EGCG-based nanocoatings, the marked enhancement in

CellPress
OPEN ACCESS

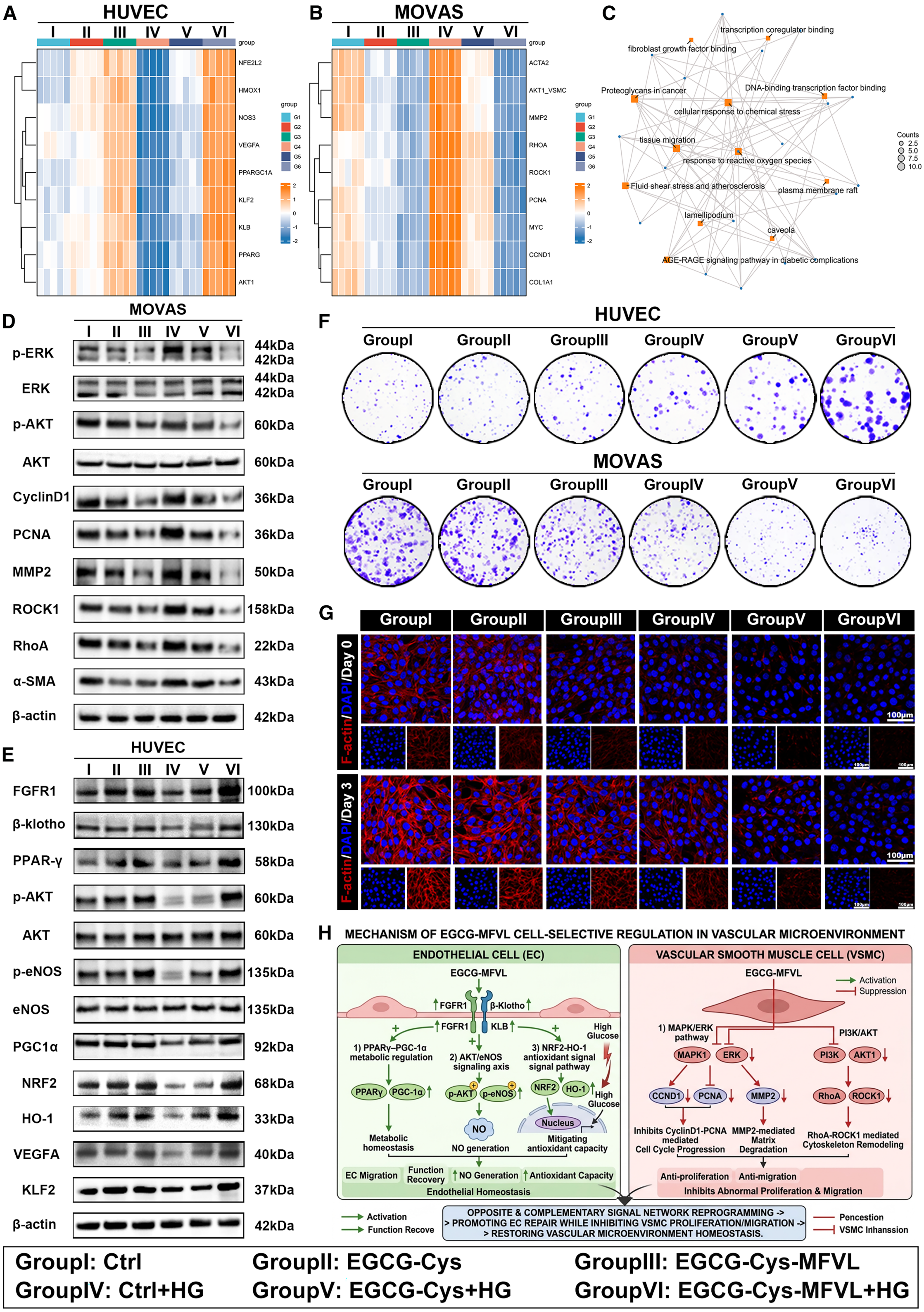

(legend on next page)

surface hydrophilicity observed here supports the growing application of EGCG-Cys functionalization in promoting vascular implant integration and biointerface optimization.[51,52] The EGCG-Cys-MFVL stent exhibited a uniformly coated nanostructured surface distinct from the smooth morphology of the bare stent, as evidenced by SEM imaging (Figure 1G, left). Energy-dispersive X-ray spectroscopy (EDS) elemental mapping confirmed the successful incorporation and homogeneous distribution of carbon (C), sulfur (S), and Mn elements, indicating the stable anchoring of the EGCG-Cys-MFVL nanocoating (Figure 1G, right). The persistent Mg signal reflects the underlying stent matrix, while the enhanced Mn signal highlights effective integration of MnO_2 nanodomains. These results validate the structural integrity and compositional precision of the nanocoating, underscoring its feasibility as a multifunctional vascular interface.

To systematically evaluate corrosion resistance and interfacial stability, time-resolved degradation analyses of EGCG-Cys-MFVL-coated WE43 stents were performed under both static and dynamic conditions. SEM revealed rapid corrosion of bare WE43, characterized by extensive crack formation and porous degradation at 3 and 6 months, whereas the coated stents maintained a compact surface morphology with suppressed crack propagation, indicating effective protection against Mg alloy degradation (Figure 1H). Consistently, degradation-induced alkalization was attenuated, as reflected by stabilized pH profiles, and accompanied by a reduced Mg^{2+} release rate, supporting modulation of corrosion kinetics and delayed bulk degradation (Figures 1I and 1J). Time-dependent EDS analysis further demonstrated sustained retention of coating-associated elements (C, S, and Mn) without evident redistribution, indicating stable interfacial integration (Figure S1Q). Under physiologically relevant shear stress (0.5–2 Pa), fluorescence imaging showed minimal signal attenuation over time, supporting the structural stability of the disulfide-crosslinked EGCG-Cys matrix under dynamic flow conditions (Figures S1R and S1S). In addition, stents with different diameters (2, 3, and 4 mm) were characterized to assess geometric adaptability and mechanical performance across clinically relevant vessel calibers. Uniform fluorescence distribution was observed across all sizes, indicating consistent coating coverage, while radial force-diameter analysis demonstrated stable mechanical responses (Figures S1T and S1U).

Multifaceted prevention and microenvironmental remodeling of ISR by the EGCG-Cys-MFVL-WE43 stent *in vitro*

To evaluate the *in vitro* efficacy of the EGCG-Cys-MFVL nanocoating in preventing ISR, functionalized disc models were initially fabricated to simulate stent surface conditions. Surface functionalization of the WE43 alloy with EGCG-Cys-MFVL nanocoatings resulted in a uniform, adherent film with distinct coloration, confirming successful surface modification (Figure S1V). Macrophages, SMCs, and endothelial cells are key immunoregulatory mediators in the pathogenesis of ISR.[53] The pathological cascade is characterized by pro-inflammatory macrophage infiltration, uncontrolled SMC proliferation, and endothelial dysfunction, collectively driving neointimal hyperplasia and vascular occlusion.[54]

To recapitulate the ISR-relevant immune microenvironment *in vitro*, RAW264.7 macrophages, MOVAS smooth muscle cells, and HUVECs were used to model macrophage, vascular smooth muscle, and endothelial compartments, respectively. Atomic force microscopy (AFM) analysis revealed that the incorporation of MFVL into the EGCG-Cys nanocoating significantly increased surface roughness, resulting in a more heterogeneous nanotopography conducive to cell-material interactions (Figures S1W and S1X). The CCK-8 assays demonstrated that both the MFVL and EGCG-Cys-MFVL formulations exhibited high cytocompatibility across MOVAS VSMCs, RAW264.7 macrophages, and HUVECs, with the EGCG-Cys-MFVL group maintaining enhanced cell viability at higher concentrations, indicating favorable biosafety and surface engineering potential (Figure S1Y). Given its enhanced biocompatibility and selective anti-hyperplastic properties, the EGCG-Cys (200 μg/mL)-MFVL (100 μg/mL) formulation was selected for downstream therapeutic experiments.

To elucidate cell-selective regulation by EGCG-Cys-MFVL under ISR-like conditions, we used HUVECs and MOVAS to model endothelial cells and VSMCs, respectively, followed by transcriptomic profiling. Distinct gene expression patterns were observed across treatment groups (Figures 2A and 2B), with functional enrichment analyses highlighting pathways associated with oxidative stress, cytoskeletal organization, and vascular remodeling (Figure 2C). Extended transcriptomic analyses further revealed coordinated modulation of inflammatory and profibrotic signaling networks, including cytokine-cytokine

Figure 2. Differential regulation of endothelial and smooth muscle cells by EGCG-Cys-MFVL-WE43 stent under ISR-like conditions *in vitro*

(A and B) Heatmaps of relative gene expression profiles in HUVECs (A) and MOVAS cells (B) across different treatment groups.

(C) Functional enrichment network of differentially regulated pathways associated with oxidative stress response, cytoskeletal organization, and vascular remodeling.

(D) Western blot analysis of proliferation- and migration-related signaling pathways in MOVAS cells, including MAPK/ERK and PI3K/AKT pathways and downstream effectors (Cyclin D1, PCNA, MMP2, ROCK1, and RhoA).

(E) Western blot analysis of endothelial-function-related signaling pathways in HUVECs, including FGFR1/β-klotho signaling, PPARγ/PGC1α metabolic regulation, and AKT/eNOS, and NRF2/HO-1 pathways.

(F) Colony formation assay of HUVECs and MOVAS cells across different treatment groups.

(G) Immunofluorescence staining of F-actin (red) and nuclei (DAPI, blue) in MOVAS cells at indicated time points, with day 0 (top) and day 3 (bottom). Scale bars, 100 μm.

(H) Schematic illustration of EGCG-Cys-MFVL-mediated differential regulation in the vascular microenvironment. Group I: Ctrl; group II: EGCG-Cys; group III: EGCG-Cys-MFVL; group IV: Ctrl + HG; group V: EGCG-Cys + HG; group VI: EGCG-Cys-MFVL + HG. HG, high-glucose condition mimicking a pathological ISR-like microenvironment.

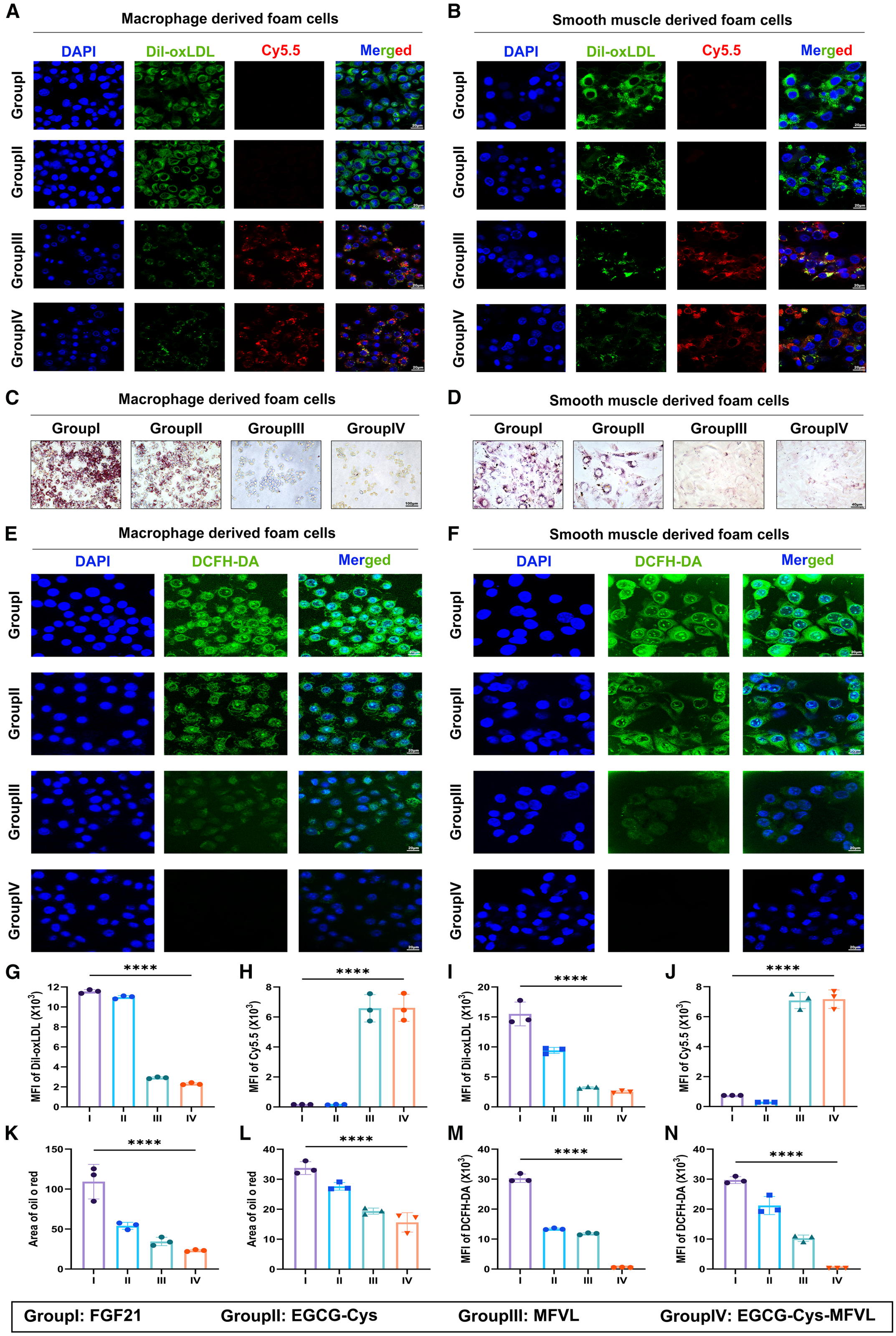

(legend on next page)

receptor interaction and chemokine pathways (Figures S2A–S2F). Consistent with these findings, qPCR validation demonstrated upregulation of endothelial-function-associated genes, including *FGFR1*, *PPARΓ*, *B-KLOTHO*, *PGC1A*, *AKT*, *ENOS*, *NRF2*, *HO-1*, *VEGFA*, and *KLF2*, alongside downregulation of genes related to VSMC proliferation and migration, such as *ERK*, *AKT*, *Cyclin D1*, *PCNA*, *MMP2*, *ROCK1*, *RhoA*, and *α-SMA* (Figures S2G and S2H). At the protein level, EGCG-Cys-MFVL differentially modulated key signaling pathways, with activation of FGFR1/β-klotho, PPARγ/PGC1α-mediated metabolic signaling, and AKT/eNOS and NRF2/HO-1 pathways in endothelial cells and suppression of MAPK/ERK and PI3K/AKT cascades together with downstream effectors including Cyclin D1, PCNA, MMP2, ROCK1, and RhoA in VSMCs (Figures 2D and 2E; Figures S2I and S2J). Functional assays demonstrated reduced proliferative capacity in VSMCs and enhanced proliferation in endothelial cells, as evidenced by colony formation and corresponding quantification (Figure 2F; Figure S2K), while immunofluorescence analysis revealed decreased F-actin organization in VSMCs over time (Figure 2G; Figure S2L). Under inflammatory ISR-like conditions induced by lipopolysaccharide (LPS), wound healing assays further showed suppressed migration in VSMCs and enhanced migratory capacity in endothelial cells (Figures S3A and S3B). Integrating these multi-level findings, a unified mechanistic framework emerges (Figure 2H), in which EGCG-Cys-MFVL orchestrates coordinated, cell-selective signaling reprogramming within the vascular microenvironment: activation of FGFR1/β-klotho, PPARγ/PGC1α, and AKT/eNOS-NRF2/HO-1 axes supports endothelial repair, metabolic homeostasis, and redox balance, whereas concurrent inhibition of MAPK/ERK and PI3K/AKT pathways with downstream cytoskeletal and proliferation regulators constrains VSMC activation. This bidirectional regulatory architecture underlies the restoration of vascular microenvironment homeostasis under ISR-like conditions.

The EGCG-Cys-MFVL nanocoating achieved cell-selective regulation by synergistically integrating redox-responsiveness, metabolic modulation, and biointerface engineering. The MnO_2 NFs impart ROS-scavenging capacity and catalyze the oxidative degradation of the nanocoating, enabling site-specific release of FGF21. This metabolic hormone enhances endothelial regeneration through peroxisome-proliferator-activated receptor gamma (PPARG/PPARγ)-PPARG coactivator 1 alpha (PPARGC1A/PGC-1α) signaling and mitochondrial activation, thereby promoting HUVEC proliferation and migration.[55] Simultaneously, EGCG inhibits SMC proliferation by modulating multiple pathways, including suppressing mitogen-activated protein kinase (MAPK), phosphoinositide 3-kinase (PI3K)/protein kinase B (AKT), and nuclear factor κB (NF-κB) signaling; inducing G_0/G_1 cell-cycle arrest; and downregulating matrix metallopeptidase 2 (MMP2) expression, thereby establishing a mechanistic basis for its anti-restenotic efficacy in the biofunctional nanocoating.[56] The observed attenuation of F-actin integrity and clonogenic potential in VSMCs reflects impaired mechanotransduction and motility.[57]

To further validate the anti-atherogenic potential of the EGCG-Cys-MFVL nanocoating *in vitro*, a biomimetic atherosclerotic microenvironment was established using macrophage- and smooth-muscle-derived foam cells as cellular surrogates for the immune and vascular components. Foam cells, primarily derived from macrophages and phenotypically modulated SMCs upon exposure to oxidized LDL (oxLDL), are central mediators of neointimal hyperplasia and structural remodeling in ISR lesions.[58] Their accumulation drives lipid deposition, oxidative stress, and chronic inflammation, reinforcing a pro-restenotic microenvironment.[58] The EGCG-Cys-MFVL nanocoating (group IV) markedly suppressed oxLDL uptake, intracellular lipid accumulation, and oxidative stress in both macrophage- and SMC-derived foam cells (Figures 3A–3F). This therapeutic effect is closely linked to the efficient targeting capacity of MFVL nanoparticles, which is enhanced by conjugation with the VHPK peptide. The VHPK peptide exhibits high affinity for VCAM-1, a key inflammatory marker that is upregulated on foam cells within atherosclerotic plaques.[59] The ligand-receptor interaction between the VHPK peptide and VCAM-1 facilitates selective nanoparticle accumulation and cellular internalization within inflamed, foam-cell-rich microenvironments, thereby amplifying the precision and efficacy of local biointerface modulation.[59,60]

Compared to FGF21 (group I), EGCG-Cys (group II), and MFVL (group III) alone, the EGCG-Cys-MFVL nanocoating exhibited markedly lower Dil-oxLDL fluorescence intensities (Figures 3G–3J), minimal ORO-positive lipid deposition (Figures 3K and 3L), and significantly reduced intracellular ROS levels (Figures 3M and 3N). These results highlight the enhanced capability of the multifunctional nanocoating in simultaneously regulating lipid accumulation and oxidative stress within foam-cell-enriched microenvironments. To further delineate the module-specific contributions underlying ROS regulation, intracellular ROS levels were systematically evaluated in macrophage- and smooth-muscle-cell-derived foam cells (Figures S3C–S3F).

Mechanistically, this therapeutic advantage arises from the rational integration of antioxidant, anti-inflammatory, and metabolic regulatory components within the nanocoating matrix.

Figure 3. The EGCG-Cys-MFVL nanocoating modulates the *in vitro* oxidized lipid microenvironment characteristic of atherosclerotic lesions

(A and B) Representative confocal images of macrophage-derived (A) and smooth muscle-derived (B) foam cells after co-culturing with different nanocoated substrates (group I: FGF21, group II: EGCG-Cys, group III: MFVL, group IV: EGCG-Cys-MFVL), stained with DAPI (blue), Dil-oxLDL (green), and Cy5.5-labeled MFVL (red). Scale bars, 20 μm.

(C and D) ORO staining of macrophage-derived (C) and smooth muscle-derived (D) foam cells to assess intracellular lipid accumulation. Scale bars, 100 μm.

(E and F) Intracellular ROS detection in macrophage-derived (E) and smooth-muscle-derived (F) foam cells using the DCFH-DA fluorescence probe. Scale bars, 20 μm.

(G–J) Quantitative analysis of MFI of Dil-oxLDL (G and I) and Cy5.5 (H and J) signals in macrophage- and smooth-muscle-derived foam cells, respectively.

(K and L) Quantification of the lipid accumulation area from ORO staining in macrophage- and smooth-muscle-derived foam cells.

(M and N) Quantification of intracellular ROS levels (MFI of DCFH-DA) in macrophage- and smooth-muscle-derived foam cells. Data are shown as mean ± SD. Statistical significance was calculated by one-way ANOVA with Tukey's test. Significance: *$p < 0.05$; **$p < 0.01$; ***$p < 0.001$; ****$p < 0.0001$.

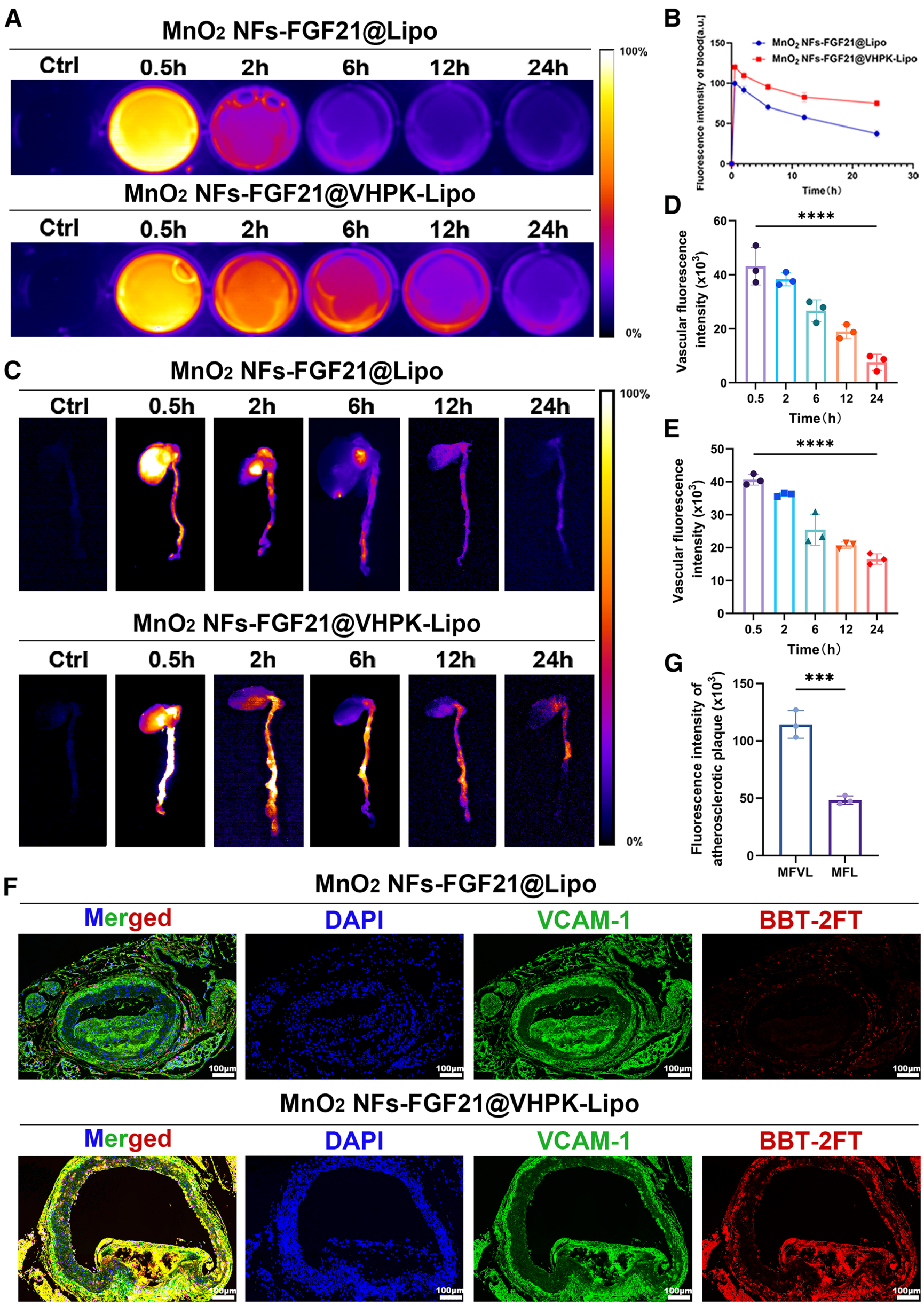

Figure 4. *In vivo* plaque-targeting performance of the MFVL nanoplatform

(A) Time-lapse NIR-II fluorescence imaging of blood collected from mice administered MnO_2 NF-FGF21@Liposomes (Lipo, top) or MFVL (bottom) at 0.5, 2, 6, 12, and 24 h.

(B) Quantitative fluorescence intensity of blood over time for both groups.

(C) *Ex vivo* NIR-II fluorescence imaging of aortas at different time points post-injection of Lipo (top) or MFVL (bottom).

(D and E) Quantification of vascular fluorescence intensity in the Lipo (D) and MFVL (E) groups.

(F) Immunofluorescence staining of aortic sections showing VCAM-1 (green) expression and colocalization with NIR-II fluorescence signal (BBT-2FT, red) in mice treated with MnO_2 NFs-FGF21@Lipo (top) or MnO_2 NFs-FGF21@VHPK-Lipo (MFVL, bottom). Nuclei are stained with DAPI (blue). Scale bars, 100 μm.

(G) Quantification of fluorescence intensity in atherosclerotic plaques, comparing MFVL and non-targeted MFL groups. Data are shown as mean ± SD. Statistical significance was calculated by one-way ANOVA with Tukey's test. Significance: $^{*}p < 0.05$; $^{**}p < 0.01$; $^{***}p < 0.001$; $^{****}p < 0.0001$.

EGCG downregulates scavenger receptors CD36 and LOX-1 to reduce oxLDL uptake, while activating NRF2 and inhibiting NF-κB to suppress ROS.[61,62] The embedded MnO_2 nanostructures function as redox-active nanozymes with catalase- and peroxidase-mimetic activity, efficiently neutralizing ROS and enabling redox-triggered release of FGF21. Once released, FGF21 promotes mitochondrial biogenesis and lipid catabolism through the PPARγ-PGC-1α axis, further contributing to microenvironmental remodeling.[63] In parallel, the cystamine-mediated disulfide crosslinking imparts redox-responsiveness to the polymeric matrix, allowing spatiotemporally controlled drug release in response to oxidative cues. Altogether, these synergistic effects at the nanocoating-cell interface facilitate targeted, environment-adaptive regulation of foam cell behavior and oxidized lipid homeostasis, providing a substantial materials-based strategy for atherosclerotic plaque intervention.

Targeted prevention and multifactorial microenvironmental modulation of ISR by the EGCG-Cys-MFVL-WE43 stent *in vivo*

To elucidate the *in vivo* plaque-targeting behavior of the MFVL nanoplatform, real-time and comprehensive tracking was conducted using both near-infrared I (NIR-I) and NIR-II fluorescence imaging. The *in vivo* pharmacokinetic and biodistribution profiles of the MFVL nanoplatform demonstrated markedly enhanced plaque-targeting performance compared to the non-targeted formulation (MnO_2 NF-FGF21@Liposomes). NIR-II fluorescence imaging of whole blood over a 24-h period revealed that the MFVL group maintained over 65% of its initial signal at 6 h and approximately 40% at 12 h, significantly higher than the rapid clearance observed in the MnO_2 NF-FGF21@Liposomes (Lipo) group (Figures 4A and 4B).

Ex vivo imaging of isolated aortas demonstrated consistently higher vascular accumulation of MFVL across all examined time points, with fluorescence intensity in the VHPK-modified group markedly exceeding that of the control group (Figures 4C–4E). Cross-sectional imaging confirmed enhanced plaque localization (Figure S3G), which was quantitatively confirmed by an approximately 3.6-fold increase in plaque-associated

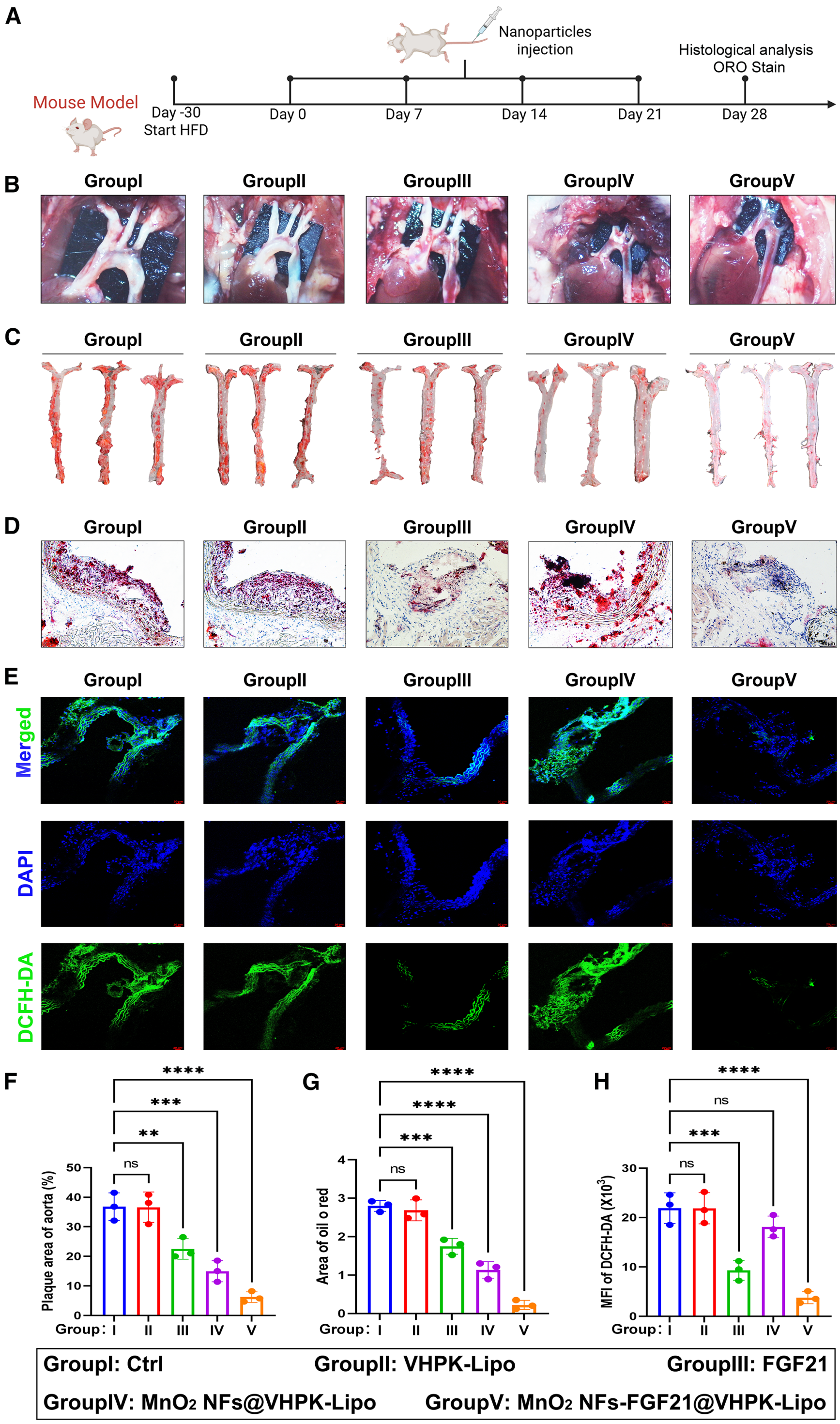

Figure 5. *In vivo* anti-atherosclerotic and antioxidative efficacy of the MFVL nanoplatform

(A) Schematic representation of the experimental timeline in *Apoe*$^{-/-}$ mice. The mice were pre-fed a high-fat diet for 30 days, followed by intravenous injection of different nanoparticle formulations on days 7 and 14. On day 28, the mice were sacrificed for histological analysis, including ORO staining and ROS detection.

(B) Stereomicroscopic images of aortic roots from *Apoe*$^{-/-}$ mice in five experimental groups post-treatment: Ctrl (group I), VHPK-Lipo (group II), FGF21 (group III), MnO_2 NF@VHPK-Lipo (group IV), and MFVL (group V).

(C) *En face* ORO staining of isolated aortas from *Apoe*$^{-/-}$ mice.

(D) Cross-sectional ORO staining of aortic roots. Scale bars, 40 μm.

(E) Immunofluorescence staining of aortic sections for ROS detection using DCFH-DA (green) and nuclear staining (DAPI, blue); merged images are shown in the top row. Scale bars, 50 μm.

(F) Quantification of plaque area in whole aortas (%).

(G) Quantification of ORO^{+} areas in aortic root sections.

(H) Quantification of the MFI of DCFH-DA staining for ROS analysis. The scale bars represent 20 μm.

Data are shown as mean ± SD. Statistical significance was calculated by one-way ANOVA with Tukey's test. Significance: *$p < 0.05$; **$p < 0.01$; ***$p < 0.001$; ****$p < 0.0001$.

fluorescence intensity compared with the Lipo group (Figure S3H). Immunofluorescence analysis provided additional mechanistic evidence for targeting specificity, showing pronounced colocalization of MFVL-derived NIR-II signals with VCAM-1-positive regions in atherosclerotic plaques, whereas minimal overlap was observed in non-targeted controls (Figure 4F**)**. Consistently, quantitative analysis demonstrated significantly enhanced plaque accumulation in the MFVL group relative to the non-targeted MFL group (Figure 4G**)**. Notably, fluorescence signals were spatially confined to VCAM-1-enriched regions, with negligible distribution in adjacent normal vascular endothelium, indicating receptor-dependent targeting precision and minimal off-target accumulation under conditions of low VCAM-1 expression.

To evaluate the *in vivo* therapeutic efficacy of the MFVL nanoplatform, a comprehensive assessment of atherosclerotic plaque burden and oxidative stress modulation was performed in *Apoe*$^{-/-}$ mice (Figure 5A). Stereomicroscopic examination of aortic roots revealed a markedly reduced plaque burden in the MFVL (group V) group compared with the Ctrl and other treatment groups (Figure 5B). Consistently, *en face* oil red O (ORO) staining of isolated aortas demonstrated a substantial reduction in lipid deposition following MFVL administration (Figure 5C), which was

further corroborated by histological analysis of aortic root cross-sections showing diminished lipid accumulation (Figure 5D). Quantification confirmed significantly reduced plaque burden (Figure 5F). Similarly, the ORO-positive area in aortic root sections was markedly reduced in group V (0.42 ± 0.13 mm^2) relative to groups I–III ($p < 0.001$; Figure 5G). To further assess oxidative stress within the vascular microenvironment, DCFH-DA fluorescence staining was performed. Representative images showed substantially attenuated ROS signals in group V (Figure 5E), which was quantitatively confirmed by a pronounced reduction in mean fluorescence intensity (MFI) ($6.3 \pm 1.0 \times 10^3$) compared with group I ($26.9 \pm 2.6 \times 10^3$, $p < 0.0001$), group II ($25.7 \pm 3.1 \times 10^3$, $p < 0.0001$), and group III ($18.8 \pm 2.3 \times 10^3$, $p < 0.001$; Figure 5H). Longitudinal MRI analysis further substantiated these findings, revealing progressive plaque development in the AS group, whereas treatment with MnO_2-FGF21@Liposomes (group II) and MFVL (group III) effectively attenuated lesion progression (Figures S4A–S4E). To further determine whether these therapeutic effects can be maintained over extended time scales, long-term *in vivo* evaluation was performed in *Apoe*$^{-/-}$ mice (Figures S4F–S4H). Serial macroscopic and histological analyses across 1 to 12 months demonstrated a progressive and sustained reduction in plaque burden, accompanied by preservation of vascular architecture and attenuation of neointimal remodeling. Quantitative measurements consistently revealed time-dependent decreases in lesion area and plaque occupancy, indicating sustained attenuation of disease progression without evidence of rebound or pathological reactivation. These findings establish the long-term stability of MFVL-mediated therapeutic efficacy within a chronically inflamed vascular microenvironment.

Building upon these results, we next evaluated the anti-ISR efficacy in preclinical models of the EGCG-Cys-MFVL-WE43 stent using multimodal imaging across rodent, rabbit, and porcine models of vascular intervention (Figure 6A; Figures S4I and S4J; Video S1). In the rat carotid artery stenting model, MRI revealed significantly reduced signal loss and preserved lumen continuity in the EGCG-Cys-MFVL-WE43 group compared to the WE43 and AS groups, indicating mitigated ISR (Figure 6B). In the rabbit model, serial computed tomography angiography (CTA)-based 3D reconstructions demonstrated progressive luminal occlusion over 28 days in the bare WE43 group, whereas the EGCG-Cys-MFVL-WE43 stents maintained vessel patency with minimal ISR development (Figure 6C). Quantitative analysis showed a significant reduction in restenotic volume at day 28 in the EGCG-Cys-MFVL-WE43 group compared to the WE43 group ($p < 0.01$, $n = 5$). In the porcine coronary stenting model, high-resolution CTA imaging following 3 months of high-fat diet feeding revealed notable neointimal hyperplasia in the WE43 group (red arrows), whereas the EGCG-Cys-MFVL-WE43 group preserved clear luminal architecture (Figure 6D). Corresponding intravascular ultrasound (IVUS) cross-sectional and longitudinal scans further confirmed these findings (Figure 6E). Quantitative IVUS analysis revealed a significantly greater minimal lumen area (MLA) in the EGCG-Cys-MFVL-WE43 group (6.42 ± 0.41 mm^2) compared to the WE43 group (3.87 ± 0.35 mm^2; $p < 0.001$, $n = 4$) and a smaller neointimal area ($p < 0.01$).

Longitudinal CT-based evaluation revealed that, in contrast to the progressive luminal collapse observed in the bare WE43 group, the EGCG-Cys-MFVL-WE43 stent maintained a stable minimal lumen area over 12 months (Figures S4K–S4N). Quantitative analysis further showed a markedly lower cumulative target lesion failure (TLF) incidence and reduced luminal area stenosis compared with both bare WE43 and drug-eluting stents (Figure S4O). Importantly, radial force-diameter profiling demonstrated gradual yet preserved mechanical support during degradation, indicating a temporally synchronized transition from structural support to bioactive modulation (Figure S4P). Notably, head-to-head comparisons establish its enhanced long-term performance over conventional WE43 and drug-eluting stents across key clinical surrogate endpoints, including TLF, lumen preservation, and neointimal suppression. This integrated functional-mechanical synergy underscores the potential applicability of the multifunctional nanocoating for long-term mitigation of ISR.

EGCG-Cys-MFVL-WE43-stent-induced macrophage polarization reprogramming for modulating the immune microenvironment in ISR

A comprehensive *in vivo* assessment was conducted to evaluate the immunomodulatory efficacy and hemocompatibility of the EGCG-Cys-MFVL nanoengineered stent nanocoating. Following a 1-month implantation in a rat carotid artery plaque model, high-throughput transcriptomic sequencing of explanted arteries was performed to assess the molecular impact of the EGCG-Cys-MFVL-coated and bare (Ctrl) WE43 stents. Weighted gene co-expression network analysis (WGCNA) revealed distinct gene module clustering and expression patterns between groups, with the EGCG-Cys-MFVL group exhibiting substantial transcriptomic reprogramming indicative of altered immune and inflammatory signaling (Figure 7A; Figures S5A–S5D).

Differential gene expression analysis demonstrated that EGCG-Cys-MFVL-coated stents significantly upregulated anti-inflammatory and M2-macrophage-polarization-related genes (e.g., arginase [*Arg1*], interleukin-10 receptor alpha [*Il10ra*], and interleukin-4-induced 1 [*Il4i1*]), whereas bare stents predominantly induced pro-inflammatory and M1-associated markers (e.g., *Cd86*, interleukin-1 alpha [*Il1a*], and interleukin-18 [*Il18*]), suggesting a pronounced immunoregulatory shift toward a reparative macrophage phenotype in the EGCG-Cys-MFVL group (Figure 7B). Consistently, metascape-based enrichment analysis further highlighted the activation of cytokine-mediated signaling cascades and immune regulatory pathways, reinforcing the anti-inflammatory remodeling of the local vascular microenvironment (Figure 7C**)**.

Immunofluorescence analysis of neointimal sections revealed a significant shift in macrophage polarization following stent implantation. Compared to the Ctrl group (group I), the EGCG-Cys-MFVL-coated stents (group IV) induced a ~90% reduction in $CD86^+$ M1 macrophage fluorescence intensity and a ~3-fold increase in $CD206^+$ M2 macrophage expression, indicating a substantial anti-inflammatory reprogramming effect (Figures 7D–7F). Notably, while the EGCG-Cys (group II) and MFVL (group III) nanocoatings individually showed partial immunomodulatory effects, the dual-functional EGCG-Cys-MFVL nanocoating

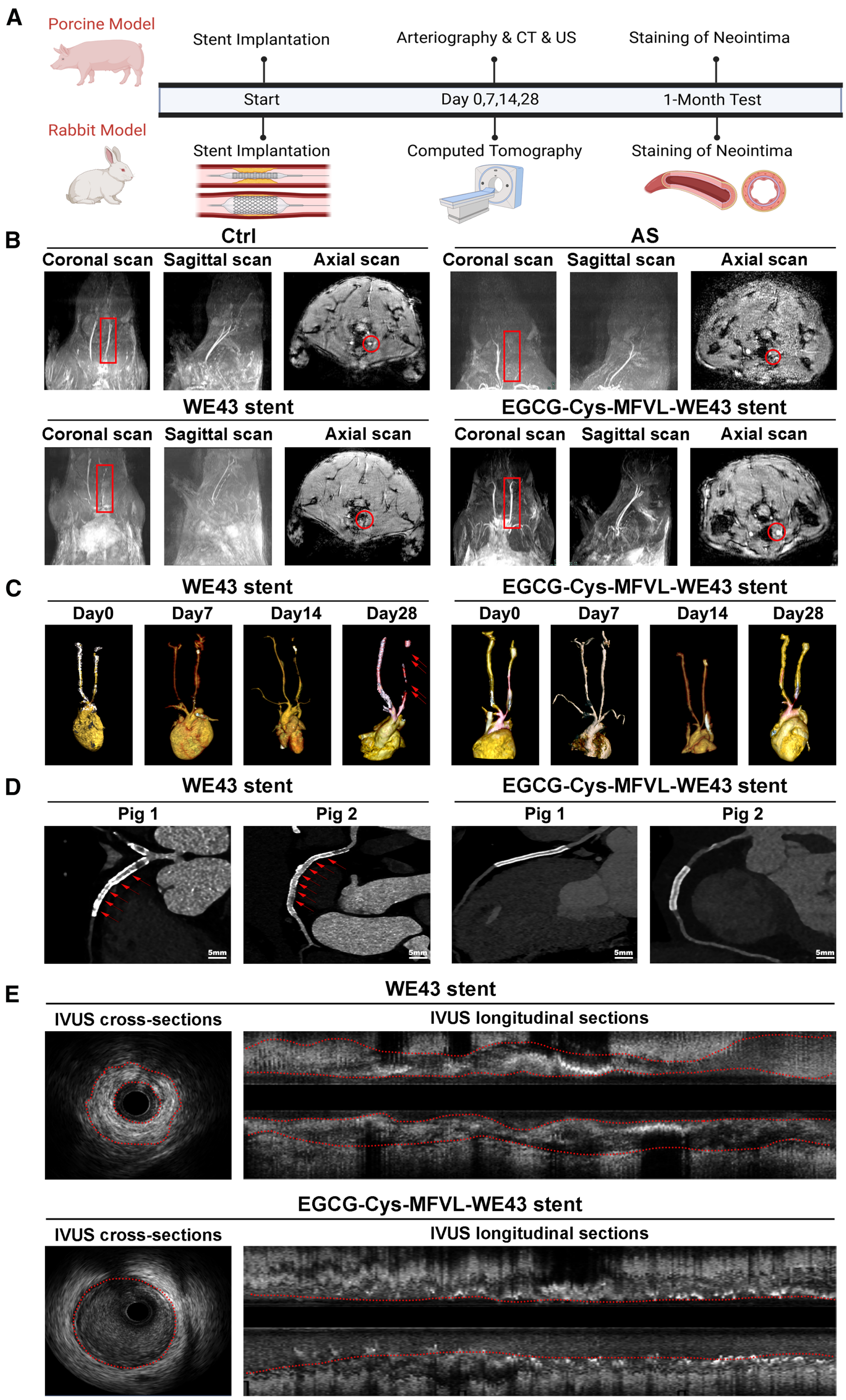

Figure 6. *In vivo* anti-ISR efficacy of the EGCG-Cys-MFVL-WE43 stent

(A) Evaluation timeline in porcine and rabbit models. The stents were implanted and monitored at days 0, 7, 14, and 28 via computed tomography (CT), IVUS, and CTA, followed by neointimal harvesting at 1 month for histological analysis.

(B) Representative MRI images of the left common carotid arteries from rats in four experimental groups post-stent implantation: Ctrl, AS, WE43 stent, and EGCG-Cys-MFVL-WE43 stent.

(C) CTA-based 3D reconstructions of the left common carotid arteries from rabbits implanted with WE43 or EGCG-Cys-MFVL-WE43 stents at days 0, 7, 14, and 28 post-implantation; red arrows indicate regions of ISR.

(D) Coronary CTA images of pigs implanted with WE43 or EGCG-Cys-MFVL-WE43 stents after 3 months of a high-fat diet; red arrows indicate regions of ISR in the WE43 stent group.

(E) IVUS cross-sectional and longitudinal images of coronary arteries from pigs implanted with WE43 or EGCG-Cys-MFVL-WE43 stents. Corresponding to the models in (D), red dotted lines delineate the vessel lumen and stent boundaries for ISR evaluation.

exhibited the most pronounced suppression of pro-inflammatory M1 markers and enhancement of reparative M2 markers. These trends were further validated by flow cytometry analysis of aortic single-cell suspensions (Figure S5E). Quantification revealed a stepwise decrease in $CD86^{+}$ $F4/80^{+}$ M1-like macrophages across groups II and III, with group IV showing the lowest M1 ratio compared to group I (11.4% vs. 36.5%). Conversely, the proportion of $CD206^{+}$ $F4/80^{+}$ M2-like macrophages was significantly elevated in group IV compared to group I (42.4% vs. 23.4%), confirming a biomaterial-driven polarization toward a tissue-reparative phenotype (Figure S5F). These findings were further corroborated at the protein level by western blot analysis of aortic single-cell suspensions (Figures S5G and S5H).

To further assess the long-term stability of macrophage polarization reprogramming *in vivo*, extended immunofluorescence analyses were performed in rabbit stenting models up to 12 months (Figures S5I–S5M). In the bare WE43 group, a progressive increase in $CD86^{+}$ M1-like macrophages accompanied by a concomitant decline in $CD206^{+}$ M2-like macrophages was observed over time, indicating sustained pro-inflammatory activation and potential polarization reversal under chronic vascular injury. In contrast, the EGCG-Cys-MFVL-WE43 stent maintained a stable and persistent M2-dominant phenotype throughout the entire observation period, with sustained CD206 expression and suppressed CD86 signals from 1 to 12 months. Quantitative analysis further confirmed a time-dependent divergence between groups, highlighting the durability of M2 polarization without evidence of phenotypic reversion. To further evaluate whether this immunomodulatory effect depends on host immune competence, a

CellPress
OPEN ACCESS

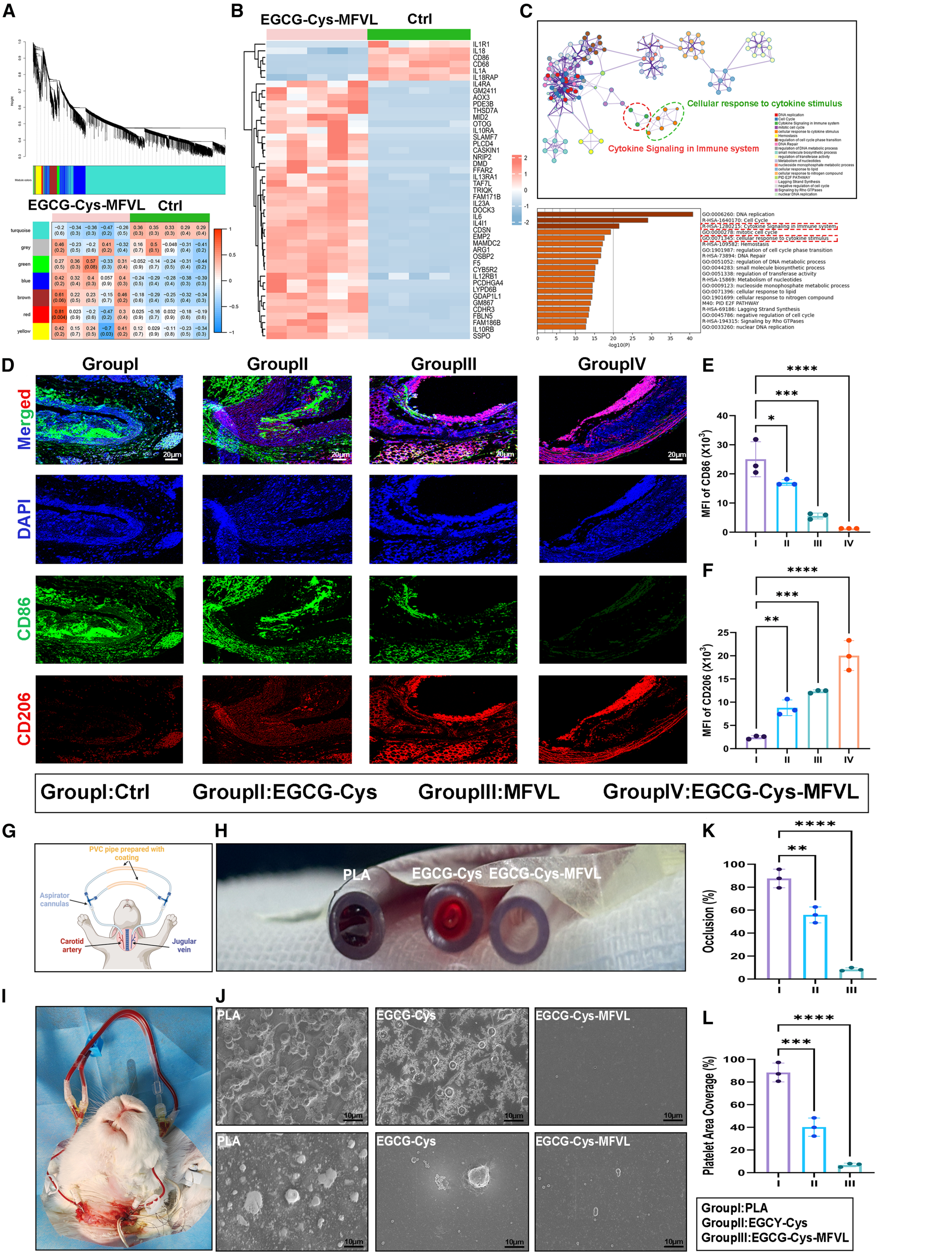

(legend on next page)

RAG2$^{-/-}$ IL2RG$^{-/-}$ severe combined immunodeficient (SCID-like) rabbit model was established (Figures S6A–S6E). Notably, despite the overall attenuated immune cell infiltration, the EGCG-Cys-MFVL-WE43 stent continued to promote a preferential shift toward CD206$^+$ macrophages while suppressing CD86 expression across early (7–15 days) and intermediate (1–3 months) time points. In contrast, the bare WE43 group exhibited minimal polarization control and a gradual predominance of M1-like signals.

To further bridge the gap between conventional preclinical models and the pathological complexity of human ISR—particularly the prevalence of calcified lesions in advanced coronary disease—we next evaluated whether the microenvironment-regulatory effects of the EGCG-Cys-MFVL-WE43 stent could be sustained under calcification-prone conditions. Progressive Ca^{2+} deposition was observed at the peri-stent vascular interface in the bare WE43 group over time, indicative of ongoing mineralization and adverse vascular remodeling. In contrast, the EGCG-Cys-MFVL-WE43 stent markedly attenuated Ca^{2+} accumulation and preserved interfacial structural integrity across all examined time points (Figure S6F). Quantitative analysis further confirmed a significantly reduced Ca^{2+} deposition burden in the EGCG-Cys-MFVL group, particularly at late stages (6–12 months), compared with the bare WE43 group (Figure S6G). Mechanistically, this suppression of calcification is likely attributable to the coordinated modulation of oxidative stress, inflammatory signaling, and lipid metabolism by the multifunctional nanocoating, thereby limiting osteogenic transdifferentiation and mineral deposition within the vascular wall. These findings demonstrate that the EGCG-Cys-MFVL platform retains substantial microenvironment-modulating capacity not only in lipid-rich lesions but also under calcification-associated pathological conditions. This expanded functional resilience addresses a key limitation of existing models and substantially strengthens the translational relevance of the platform for complex ISR phenotypes encountered in clinical settings.

The EGCG-Cys-MFVL nanocoating promotes M2 macrophage polarization through the integrated regulation of inflammatory, oxidative, and metabolic pathways. EGCG inhibits NF-κB and MAPK signaling to suppress M1-associated cytokines, while activating signal transducer and activator of transcription 6 (STAT6) and PPARγ to induce M2 markers such as ARG1 and interleukin-10 (IL-10).[64] MnO_2 nanozymes within the MFVL matrix efficiently scavenge ROS, alleviating oxidative stress and promoting a metabolic shift toward oxidative phosphorylation to stabilize M2 polarization.[65] The redox-triggered release of FGF21 further activates the PPARγ-PGC-1α axis, enhancing mitochondrial function and anti-inflammatory signaling.[63] The disulfide-crosslinked structure confers redox-responsiveness for on-demand degradation and targeted therapeutic release. These synergistic mechanisms converge at the material-immune interface to remodel the vascular immune microenvironment, establishing EGCG-Cys-MFVL as a scientifically rational and translationally promising nanoplatform for preventing ISR.

To evaluate hemocompatibility and antithrombotic performance *in vivo*, an extracorporeal arteriovenous circulation model was established in rabbits, in which coated stent segments were incorporated into a carotid jugular shunt loop (Figures 7G–7I). Macroscopic inspection revealed visible thrombus formation in the PLA (group I) and EGCG-Cys (group II) groups, whereas the EGCG-Cys-MFVL-coated stents (group III) remained nearly occlusion-free (Figure 7H). SEM further demonstrated dense red blood cell and platelet adhesion on PLA and EGCG-Cys surfaces, in stark contrast to the clean, non-adhesive morphology observed on the EGCG-Cys-MFVL nanocoating (Figure 7J). Quantitative analysis confirmed a ∼95% reduction in thrombotic occlusion rate and a >90% decrease in platelet-covered area in the EGCG-Cys-MFVL group compared to the PLA group, indicating a significant enhancement in antithrombotic performance (Figures 7K and 7L). Notably, while EGCG-Cys exhibited partial thrombosis mitigation, only the dual-functional EGCG-Cys-MFVL system achieved near-complete inhibition of both blood cell aggregation and platelet deposition. These findings highlight the synergistic hemocompatibility of the EGCG-Cys-MFVL nanocoating and underscore its potential as a multifunctional interventional platform that simultaneously prevents thrombus formation and mitigates ISR through integrated biochemical and surface-engineered strategies. Comprehensive *in vivo* safety

Figure 7. *In vivo* immunoregulatory modulation of the ISR plaque microenvironment by EGCG-Cys-MFVL nanoengineered functional stent nanocoatings

(A) WGCNA of transcriptomic data from rat carotid arteries implanted with EGCG-Cys-MFVL-coated stents (group: EGCG-Cys-MFVL) and bare stents (group: Ctrl).

(B) Heatmap of differentially expressed immune-related genes between EGCG-Cys-MFVL and Ctrl groups.

(C) Gene Ontology (GO) biological process and pathway enrichment analysis of differentially expressed genes performed using the Metascape platform, highlighting cytokine-mediated signaling cascades and immune response regulatory pathways.

(D) Immunofluorescence staining of neointimal sections from four groups—Ctrl (group I), EGCG-Cys (group II), MFVL (group III), and EGCG-Cys-MFVL (group IV)—detecting DAPI (nuclei), CD86 (M1 macrophage marker), and CD206 (M2 macrophage marker).

(E) Quantitative analysis of the MFI for CD86 in (D). Scale bars, 20 μm.

(F) Quantitative analysis of the MFI for CD206 in (D).

(G) Schematic illustration of the extracorporeal circulation model for *in vivo* thrombogenicity testing in rabbits, including carotid-jugular shunt with coated stent segments.

(H) Macroscopic images of stents from group I (PLA), group II (EGCG-Cys), and group III (EGCG-Cys-MFVL) after *in vivo* arteriovenous circulation, as shown in (g).

(I) Representative image of the rabbit extracorporeal circulation setup used for evaluating thrombogenicity and hemocompatibility.

(J) Representative SEM images of the stent luminal surfaces after blood exposure. The upper row shows red blood cell adhesion morphology; the lower row shows platelet adhesion morphology.

(K) Quantification of thrombotic occlusion percentages in (H).

(L) Quantification of platelet-covered area percentages in (J). Data are shown as mean ± SD. Statistical significance was calculated by one-way ANOVA with Tukey's test. Significance: *$p < 0.05$; **$p < 0.01$; ***$p < 0.001$; ****$p < 0.0001$.

evaluation was performed to systematically assess the systemic biocompatibility, immunogenicity, and metabolic stability of the multi-component EGCG-Cys-MFVL nanocoating platform and its MnO_2-nanozyme-based formulations. Short-term (1-month) analyses demonstrated preserved histological architecture in major organs—including the heart, liver, spleen, lung, and kidney—without evidence of necrosis, inflammatory infiltration, or tissue remodeling (Figure S7A). Hematological profiling further revealed no statistically significant differences in leukocyte counts, erythrocyte indices, or platelet parameters compared with controls (one-way ANOVA, $p > 0.05$; Figure S7B), confirming favorable acute systemic compatibility.

To rigorously address long-term safety concerns—including potential hypersensitivity to the VHPK peptide and metabolic perturbation associated with sustained FGF21 exposure—extended evaluations were conducted over a 12-month period. Longitudinal NIR-I imaging demonstrated controlled biodistribution kinetics without progressive accumulation in clearance-associated organs, particularly the liver and kidney, indicating absence of chronic organ burden (Figure S7C). Dose-escalation studies (0.12–4.8 mg/kg) defined a tolerable systemic exposure window, with no dose-dependent mortality or delayed adverse events observed under either acute or prolonged regimens (Figures S7D and S7E). Long-term evaluation showed no histological or biochemical abnormalities (Figure S7F). Correspondingly, longitudinal serum biochemical analyses demonstrated stable hepatic (alanine aminotransferase [ALT] and aspartate aminotransferase [AST]), renal (BUN), and cardiac (B-type natriuretic peptide [BNP]) function parameters across all time points ($p > 0.05$; Figure S7G), supporting preserved systemic metabolic homeostasis during prolonged administration. Complement activation markers (C3a, C5a, and sC5b-9) remained within physiological limits throughout the observation period, indicating absence of sustained complement activation or systemic immune hypersensitivity (Figure S7H). Notably, the VHPK peptide has been widely employed as a VCAM-1-targeting ligand in preclinical vascular delivery systems without reported immunogenicity or hypersensitivity reactions.[60,66] Similarly, FGF21 has demonstrated a favorable metabolic safety profile in both experimental and clinical studies, with minimal risk of sustained glucose dysregulation under therapeutic dosing.[67] These prior observations, together with our long-term complement and metabolic assessments, further support the systemic tolerability of the integrated multi-component platform.

Finally, hemocompatibility—an essential requirement for blood-contacting vascular devices—was confirmed by hemolysis assays. The WE43 alloy, EGCG-Cys, and EGCG-Cys-MFVL groups exhibited minimal red blood cell disruption, with clear supernatants comparable to the physiological saline control, whereas the deionized water group showed pronounced hemolysis (Figures S7I and S7J). Spectrophotometric quantification further demonstrated suppressed hemoglobin release in the EGCG-based nanocoatings, particularly the EGCG-Cys-MFVL formulation, confirming blood compatibility of the engineered surfaces.

Limitations of the study

(1) Animal models may not fully recapitulate the complexity of human atherosclerosis, including heterogeneous plaque composition and associated comorbidities. (2) The mechanistic interactions among the functional modules were not investigated at the single-cell or spatial transcriptomic level. (3) The degradation behavior and pharmacokinetics observed under controlled experimental conditions may differ from those in clinical settings. (4) Further large-animal studies and clinical investigations are required to establish the long-term efficacy and safety of this platform. (5) Only male animals were used in this study; therefore, the influence of sex as a biological variable on the therapeutic efficacy and biosafety of the EGCG-Cys-MFVL-WE43 stent was not systematically evaluated. These represent limitations of the present study. Gender-related analysis was not applicable because no human participants were included.

RESOURCE AVAILABILITY

Lead contact

Further information and requests for resources and reagents should be directed to and will be fulfilled by the lead contact, Jiansong Ji (jjstcty@wmu.edu.cn).

Materials availability

The unique materials generated in this study, including MFVL nanoparticles and EGCG-Cys-MFVL-coated WE43 magnesium stents, are available from the lead contact upon reasonable request, subject to completion of a Materials Transfer Agreement if required.

Data and code availability

- RNA sequencing raw and processed data generated in this study have been deposited in the Genome Sequence Archive (GSA) at the National Genomics Data Center (NGDC) under BioProject accession number PRJCA063571. These data are publicly available at the time of publication and can be accessed at https://ngdc.cncb.ac.cn/bioproject/browse/PRJCA063571
- This paper does not report original code.
- Any additional information required to reanalyze the data reported in this paper is available from the lead contact upon reasonable request.

ACKNOWLEDGMENTS

The authors appreciate financial support from the Key Laboratory of Imaging Diagnosis and Minimally Invasive Intervention Research for support and guidance. This work was supported by Zhejiang Natural Science Foundation (LTGY23H180006 to J.S., LLSSY24H020004 to L.S., and LLSQN25H020001 to Y.B.), Zhejiang Medical Science Project (2024KY560 to L.S. and 2025KY499 to Y.B.), National Natural Science Foundation of China (82502491 to L.S., 82500607 to Y.B., and 82272091 to C.L.), and National Science and Technology Major Project (2023ZD0513000 to Y.B.).

AUTHOR CONTRIBUTIONS

L.S. conceived the study, designed the experiments, performed most experiments together with Y.Z. and Y.B., analyzed the data, and wrote the manuscript. Z.P., Y.W., Z.Y., X.D., C.J., J.Y., and Z.F. performed experimental investigations and analyzed data. M.Z. performed visualization. J.S. performed mechanical characterization, animal experiments, and *in vivo* evaluation. J.L., J.Z., Z.Z., G.S., C.L., L.L., J.S., and M.C. provided resources. X.L., Z.W., W.C., and J.J. supervised the study, revised the manuscript, and secured funding. Equal contribution, L.S., Y.Z., and Y.B. Joint supervision, X.L., Z.W., W.C., and J.J.

DECLARATION OF INTERESTS

The authors declare no competing interests.

STAR★METHODS

Detailed methods are provided in the online version of this paper and include the following:

- KEY RESOURCES TABLE
- EXPERIMENTAL MODEL AND STUDY PARTICIPANT DETAILS
  - Cells
  - Animal models
  - Sex as a biological variable
- METHOD DETAILS
  - Preparation of the EGCG-Cys nanocoating
  - Preparation of the MnO_2 NFs
  - Preparation of the MnO_2 NF-FGF21@VHPK-Liposomes
  - Preparation of the EGCG-Cys-MFVL nanocoating
  - Characterization of the EGCG-Cys-MFVL nanocoating
  - Radial support testing
  - ROS response behavior
  - Concentration-dependent H_2O_2 hydrolysis
  - *In vitro* functional evaluation of HUVECs, macrophages and SMCs
  - ROS scavenging
  - Evaluation of endothelial cell and VSMC migration behavior
  - Cellular uptake analysis
  - Flow cytometry analysis of M1 and M2 polarization
  - Western blotting
  - Quantitative real-time PCR (qPCR)
  - Oil Red O (ORO) staining
  - Mice *in vivo* treatment
  - High-throughput sequencing and bioinformatics analysis of the atherosclerotic microenvironment *in vivo* and *in vitro*
  - Blood fluorescence
  - Aortic fluorescence
  - Aortic cross-section confocal microscopy
  - Immunohistochemical and immunofluorescence staining analysis
  - Hemolysis rate assay
  - Vascular stent implantation in the rabbit model
  - *Ex vivo* evaluation of anti-thrombogenicity in an arteriovenous shunt assay
  - *In vitro* platelet adhesion test
  - Quantification of target lesion failure (TLF)
  - The cumulative TLF incidence was calculated as
  - Quantification of minimum lumen area (MLA)
  - Calculation of luminal area stenosis
  - Calcium deposition
  - Stent implantation in the porcine model
  - Safety evaluation
- QUANTIFICATION AND STATISTICAL ANALYSIS

SUPPLEMENTAL INFORMATION

Supplemental information can be found online at https://doi.org/10.1016/j.xcrm.2026.102980.

Received: October 24, 2025
Revised: March 23, 2026
Accepted: July 15, 2026
Published: August 6, 2026

REFERENCES

1. Barbato, E., McEntegart, M., and Gori, T. (2023). The year in cardiovascular medicine 2022: the top 10 papers in interventional cardiology. Eur. Heart J. *44*, 259–261. https://doi.org/10.1093/eurheartj/ehac778.

2. Baaten, C.C.F.M.J., Nagy, M., Bergmeier, W., Spronk, H.M.H., and van der Meijden, P.E.J. (2024). Platelet biology and function: plaque erosion vs. rupture. Eur. Heart J. *45*, 18–31. https://doi.org/10.1093/eurheartj/ehad720.

3. Nedeljkovic, I. (2018). Assessment of depression and anxiety in patients before and after percutaneous coronary intervention: A step forward in cardiac rehabilitation? Eur. J. Prev. Cardiol. *25*, 1015–1016. https://doi.org/10.1177/2047487318774094.

4. Di Biase, L., Lakkireddy, D.J., Marazzato, J., Velasco, A., Diaz, J.C., Navara, R., Chrispin, J., Rajagopalan, B., Natale, A., Mohanty, S., et al. (2024). Antithrombotic Therapy for Patients Undergoing Cardiac Electrophysiological and Interventional Procedures: JACC State-of-the-Art Review. J. Am. Coll. Cardiol. *83*, 82–108. https://doi.org/10.1016/j.jacc.2023.09.831.

5. Murphy, G., Durand, R., McCaughey, C., Naughton, A., Heron, E., and Pearson, I. (2022). Drug-eluting balloons and drug-eluting stents in the treatment of small vessel coronary artery disease: a systematic review and metanalysis of long-term clinical and angiographic outcomes. Eur. Heart J. *43*, ehac544.2037. https://doi.org/10.1093/eurheartj/ehac544.2037.

6. Moussa, I.D., Mohananey, D., Saucedo, J., Stone, G.W., Yeh, R.W., Kennedy, K.F., Waksman, R., Teirstein, P., Moses, J.W., and Simonton, C. (2020). Trends and Outcomes of Restenosis After Coronary Stent Implantation in the United States. J. Am. Coll. Cardiol. *76*, 1521–1531. https://doi.org/10.1016/j.jacc.2020.08.002.

7. Qi, L.-J., Zhang, Z.-Q., Iqbal, M., Wang, F., Li, J.-A., and Guan, S.-K. (2025). Spraying-assisted layer-by-layer assembled coatings with dual self-healing ability to resist degradation and enhance endothelialization of ZE21B alloys for vascular stents. Rare Met. *44*, 3405–3427. https://doi.org/10.1007/s12598-024-03159-w.

8. Xue, Z., Sun, X., Li, H., Iqbal, M., Qi, L., Wang, F., Hou, Y., Li, J., and Guan, S. (2024). Composite coatings of S-HA nanoparticles and Schiff base on ZE21B alloy for stronger corrosion resistance and biological performance. J. Magnes. Alloy. *12*, 4547–4560. https://doi.org/10.1016/j.jma.2023.11.009.

9. Kang, M.-H., Cheon, K.-H., Jo, K.-I., Ahn, J.-H., Kim, H.-E., Jung, H.-D., and Jang, T.-S. (2020). An asymmetric surface coating strategy for improved corrosion resistance and vascular compatibility of magnesium alloy stents. Mater. Des. *196*, 109182. https://doi.org/10.1016/j.matdes.2020.109182.

10. Pang, T.Y., Kwok, J.S., Nguyen, C.T., and Fox, K. (2021). Evaluating magnesium alloy WE43 for bioresorbable coronary stent applications. MRS Adv. *6*, 54–60. https://doi.org/10.1557/s43580-021-00012-5.

11. Maier, J.A.M. (2003). Low magnesium and atherosclerosis: an evidence-based link. Mol. Aspects Med. *24*, 137–146. https://doi.org/10.1016/s0098-2997(02)00095-x.

12. Wang, C., Liu, J., Min, S., Liu, Y., Liu, B., Hu, Y., Wang, Z., Mao, F., Wang, C., Ma, X., et al. (2023). The effect of pore size on the mechanical properties, biodegradation and osteogenic effects of additively manufactured magnesium scaffolds after high temperature oxidation: An in vitro and in vivo study. Bioact. Mater. *28*, 537–548. https://doi.org/10.1016/j.bioactmat.2023.06.009.

13. Liu, W., Huang, J., He, S., Du, R., Shi, W., Wang, Y., Du, D., Du, Y., Liu, Q., Wang, Y., et al. (2023). Senescent endothelial cells' response to the degradation of bioresorbable scaffold induces intimal dysfunction accelerating in-stent restenosis. Acta Biomater. *166*, 266–277. https://doi.org/10.1016/j.actbio.2023.05.028.

14. Maino, A., and Jaffer, F.A. (2022). Outcomes Following Plaque Erosion-Based Acute Coronary Syndromes Treated Without Stenting: The Plaque Matters. J. Am. Heart Assoc. *11*, e028184. https://doi.org/10.1161/jaha.122.028184.

15. Räber, L., and Ueki, Y. (2019). Bioresorbable Scaffolds: Unfulfilled Prophecies. Circulation *140*, 1917–1920. https://doi.org/10.1161/circulationaha.119.043773.

16. Kandzari, D.E., Angiolillo, D.J., Price, M.J., and Teirstein, P.S. (2009). Identifying the "optimal" duration of dual antiplatelet therapy after drug-eluting

stent revascularization. JACC Cardiovasc. Interv. *2*, 1279–1285. https://doi.org/10.1016/j.jcin.2009.09.012.

17. Baumer, Y., Mehta, N.N., Dey, A.K., Powell-Wiley, T.M., and Boisvert, W.A. (2020). Cholesterol crystals and atherosclerosis. Eur. Heart J. *41*, 2236–2239. https://doi.org/10.1093/eurheartj/ehaa505.
18. Cui, Y., Liu, Y., Zhao, F., Shi, D., and Chen, K. (2016). Neoatherosclerosis after Drug-Eluting Stent Implantation: Roles and Mechanisms. Oxid. Med. Cell. Longev. *2016*, 5924234. https://doi.org/10.1155/2016/5924234.
19. Guagliumi, G., Shimamura, K., Sirbu, V., Garbo, R., Boccuzzi, G., Vassileva, A., Valsecchi, O., Fiocca, L., Canova, P., Colombo, F., et al. (2018). Temporal course of vascular healing and neoatherosclerosis after implantation of durable- or biodegradable-polymer drug-eluting stents. Eur. Heart J. *39*, 2448–2456. https://doi.org/10.1093/eurheartj/ehy273.
20. Sumino, Y., Yonetsu, T., Ueno, H., Nogami, K., Misawa, T., Hada, M., Yamaguchi, M., Hoshino, M., Kanaji, Y., Sugiyama, T., and Kakuta, T. (2020). Impact of neoatherosclerosis observed at very late phase after coronary stent implantation on subsequent adverse events. Eur. Heart J. *41*, ehaa946.0310. https://doi.org/10.1093/ehjci/ehaa946.0310.
21. Huynh, K. (2020). Distinct immune microenvironments in atherosclerotic plaques. Nat. Rev. Cardiol. *17*, 7. https://doi.org/10.1038/s41569-019-0304-0.
22. Jiang, Y., Zhao, J., and Zhang, D. (2024). Manganese Dioxide-Based Nanomaterials for Medical Applications. ACS Biomater. Sci. Eng. *10*, 2680–2702. https://doi.org/10.1021/acsbiomaterials.3c01852.
23. Chiam, S.-L., Pung, S.-Y., Yeoh, F.Y., and Ahmadipour, M. (2022). Highly efficient oxidative degradation of organic dyes by manganese dioxide nanoflowers. Mater. Chem. Phys. *280*, 125848. https://doi.org/10.1016/j.matchemphys.2022.125848.
24. Su, Q., Gan, L., Zhu, Y., and Yang, X. (2021). Dual-emissive fluorescence and phosphorescence detection of cholesterol and glucose based on carbon dots-cyanuric acid complex quenched by MnO2 nanosheets. Sensor. Actuator. B Chem. *335*, 129715. https://doi.org/10.1016/j.snb.2021.129715.
25. Zecchini, S., Giovarelli, M., Perrotta, C., Morisi, F., Touvier, T., Di Renzo, I., Moscheni, C., Bassi, M.T., Cervia, D., Sandri, M., et al. (2019). Autophagy controls neonatal myogenesis by regulating the GH-IGF1 system through a NFE2L2- and DDIT3-mediated mechanism. Autophagy *15*, 58–77. https://doi.org/10.1080/15548627.2018.1507439.
26. Huang, W.P., Chen, C.Y., Lin, T.W., Kuo, C.S., Huang, H.L., Huang, P.H., and Lin, S.J. (2022). Fibroblast growth factor 21 reverses high-fat diet-induced impairment of vascular function via the anti-oxidative pathway in ApoE knockout mice. J. Cell Mol. Med. *26*, 2451–2461. https://doi.org/10.1111/jcmm.17273.
27. Kheirolomoom, A., Kim, C.W., Seo, J.W., Kumar, S., Son, D.J., Gagnon, M.K.J., Ingham, E.S., Ferrara, K.W., and Jo, H. (2015). Multifunctional Nanoparticles Facilitate Molecular Targeting and miRNA Delivery to Inhibit Atherosclerosis in ApoE(-/-) Mice. ACS Nano *9*, 8885–8897. https://doi.org/10.1021/acsnano.5b02611.
28. Dosta, P., Tamargo, I., Ramos, V., Kumar, S., Kang, D.W., Borrós, S., and Jo, H. (2021). Delivery of Anti-microRNA-712 to Inflamed Endothelial Cells Using Poly(β-amino ester) Nanoparticles Conjugated with VCAM-1 Targeting Peptide. Adv. Healthc. Mater. *10*, e2001894. https://doi.org/10.1002/adhm.202001894.
29. Ma, L., Tan, Y., Tong, Q., Cao, X., Liu, D., Ma, X., Jiang, X., and Li, X. (2024). Collagen Scaffolds Functionalized by Cu(2+)-Chelated EGCG Nanoparticles with Anti-Inflammatory, Anti-Oxidation, Vascularization, and Anti-Bacterial Activities for Accelerating Wound Healing. Adv. Healthc. Mater. *13*, e2303297. https://doi.org/10.1002/adhm.202303297.
30. He, Z., Luo, H., Wang, Z., Chen, D., Feng, Q., and Cao, X. (2023). Injectable and tissue adhesive EGCG-laden hyaluronic acid hydrogel depot for treating oxidative stress and inflammation. Carbohydr. Polym. *299*, 120180. https://doi.org/10.1016/j.carbpol.2022.120180.
31. Li, X., Wang, C., Zhou, L., Zhang, L., Li, J., Lin, S., Yu, X., Hou, J., Xiong, S., Zhao, Y., et al. (2023). Versatile dual responsive EGCG/Cys nano delivery system for atherosclerosis. Colloid Interface Sci. Commun. *55*, 100725. https://doi.org/10.1016/j.colcom.2023.100725.
32. Wang, K., Shang, T., Zhao, J., Zhang, L., Zhou, L., Deng, J., Li, X., and Wang, J. (2022). A redox-sensitive coating with disulfide bonds is a promising candidate for surface-modified interventional devices. Mater. Today Commun. *31*, 103380. https://doi.org/10.1016/j.mtcomm.2022.103380.
33. Bertozzi-Matheus, M., Bueno-Pereira, T.O., Nunes, P.R., and Sandrim, V.C. (2024). EGCG, a Green Tea Compound, Increases NO Production and Has Antioxidant Action in a Static and Shear Stress In Vitro Model of Preeclampsia. Antioxidants *13*, 158. https://doi.org/10.3390/antiox13020158.
34. Liu, S., Sun, Z., Chu, P., Li, H., Ahsan, A., Zhou, Z., Zhang, Z., Sun, B., Wu, J., Xi, Y., et al. (2017). EGCG protects against homocysteine-induced human umbilical vein endothelial cells apoptosis by modulating mitochondrial-dependent apoptotic signaling and PI3K/Akt/eNOS signaling pathways. Apoptosis *22*, 672–680. https://doi.org/10.1007/s10495-017-1360-8.
35. Xu, D., Li, C., Zi, Y., Jiang, D., Qu, F., and Zhao, X.E. (2021). MOF@MnO(2)-nanocomposites prepared usingin situmethod and recyclable cholesterol oxidase-inorganic hybrid nanoflowers for cholesterol determination. Nanotechnology *32*, 315502. https://doi.org/10.1088/1361-6528/abf692.
36. Yang, D.-P., Guo, W., Cai, Z., Chen, Y., He, X., Huang, C., Zhuang, J., and Jia, N. (2018). Highly sensitive electrochemiluminescence biosensor for cholesterol detection based on AgNPs-BSA-MnO2 nanosheets with superior biocompatibility and synergistic catalytic activity. Sensor. Actuator. B Chem. *260*, 642–649. https://doi.org/10.1016/j.snb.2018.01.096.
37. Alam, A.A., Goda, D.A., Soliman, N.A., Abdel-Meguid, D.I., El-Sharouny, E.E., and Sabry, S.A. (2022). Production and statistical optimization of cholesterol-oxidase generated by Streptomyces sp. AN strain. J. Genet. Eng. Biotechnol. *20*, 156. https://doi.org/10.1186/s43141-022-00433-1.
38. Li, X., Ji, Q., Yan, C., Zhu, Z., Yan, Z., Chen, P., Wang, Y., and Song, L. (2022). H(2)O(2)/pH Dual-Responsive Biomimetic Nanoenzyme Drugs Delivery System for Enhanced Tumor Photodynamic Therapy. Nanoscale Res. Lett. *17*, 103. https://doi.org/10.1186/s11671-022-03738-9.
39. Chen, J., An, X., Xu, L., Gao, Y., Zhou, M., and Liu, Z. (2024). Adhesive Nanoparticle-in-Microgel System with ROS Scavenging Capability and Hemostatic Activity for Postoperative Adhesion Prevention. Small *20*, e2306598. https://doi.org/10.1002/smll.202306598.
40. Wu, A., Li, M., Chen, Y., Zhang, W., Li, H., Chen, J., Gu, K., and Wang, X. (2024). Multienzyme Active Manganese Oxide Alleviates Acute Liver Injury by Mimicking Redox Regulatory System and Inhibiting Ferroptosis. Adv. Healthc. Mater. *13*, e2302556. https://doi.org/10.1002/adhm.202302556.
41. Jiang, N., Liu, X., Sui, B., Wang, J., Liu, X., and Zhang, Z. (2024). Using Hybrid MnO(2)-Au Nanoflowers to Accelerate ROS Scavenging and Wound Healing in Diabetes. Pharmaceutics *16*, 1244. https://doi.org/10.3390/pharmaceutics16101244.
42. Wu, X., Wang, Y., Wang, D., Wang, Z., Yang, M., Yang, L., Wang, F., Wang, W., and Zhang, X. (2024). Formation of EGCG oxidation self-assembled nanoparticles and their antioxidant activity in vitro and hepatic REDOX regulation activity in vivo. Food Funct. *15*, 2181–2196. https://doi.org/10.1039/d3fo05309a.
43. Yuan, L., Liu, J., Xiao, S., Wei, J., Liu, H., Li, Y., Zuo, Y., Li, Y., Wang, J., and Li, J. (2025). EGCG-Modified Bioactive Core-Shell Fibers Modulate Oxidative Stress to Synergistically Promote Vascularized Bone Regeneration. ACS Biomater. Sci. Eng. *11*, 543–555. https://doi.org/10.1021/acsbiomaterials.4c01906.
44. Yokotani, K., and Umegaki, K. (2017). Evaluation of plasma antioxidant activity in rats given excess EGCg with reference to endogenous antioxidants concentrations and assay methods. Free Radic. Res. *51*, 193–199. https://doi.org/10.1080/10715762.2017.1291941.
45. Wan, H., Li, Y., Qin, Y., An, Y., Yan, H., Liu, X., Zhang, H., Hu, C., Li, L., Fu, D., et al. (2023). Polyphenol-mediated sandwich-like coating promotes

endothelialization and vascular healing. Biomaterials *302*, 122346. https://doi.org/10.1016/j.biomaterials.2023.122346.

46. Wang, K., Shang, T., Zhang, L., Zhou, L., Liu, C., Fu, Y., Zhao, Y., Li, X., and Wang, J. (2021). Application of a Reactive Oxygen Species-Responsive Drug-Eluting Coating for Surface Modification of Vascular Stents. ACS Appl. Mater. Interfaces *13*, 35431–35443. https://doi.org/10.1021/acsami.1c08880.
47. Huang, S., Upputuri, P.K., Liu, H., Pramanik, M., and Wang, M. (2016). A dual-functional benzobisthiadiazole derivative as an effective theranostic agent for near-infrared photoacoustic imaging and photothermal therapy. J. Mater. Chem. B *4*, 1696–1703. https://doi.org/10.1039/c5tb02367j.
48. Shen, L., Chen, M., Su, Y., Bi, Y., Shu, G., Chen, W., Lu, C., Zhao, Z., Lv, L., Zou, J., et al. (2024). NIR-II Imaging for Tracking the Spatiotemporal Immune Microenvironment in Atherosclerotic Plaques. ACS Nano *18*, 34171–34185. https://doi.org/10.1021/acsnano.4c10739.
49. Bañobre-López, M., García-Hevia, L., Cerqueira, M.F., Rivadulla, F., and Gallo, J. (2018). Tunable Performance of Manganese Oxide Nanostructures as MRI Contrast Agents. Chemistry *24*, 1295–1303. https://doi.org/10.1002/chem.201704861.
50. Wang, K., Geng, S., Wang, F., Fang, B., Qian, H., Li, Y., Zhou, Y., Chen, Y., and Yu, Z. (2024). Natural epigallocatechin-3-gallocarboxylate nanoformulation loaded doxorubicin to construct a novel and low cardiotoxicity chemotherapeutic drug for high-efficiency breast cancer therapy. J. Nanobiotechnology *22*, 793. https://doi.org/10.1186/s12951-024-03069-0.
51. Lee, S., Lee, J., Byun, H., Kim, S.-j., Joo, J., Park, H.H., and shin, H. (2021). Evaluation of the anti-oxidative and ROS scavenging properties of biomaterials coated with epigallocatechin gallate for tissue engineering. Acta Biomater. *124*, 166–178. https://doi.org/10.1016/j.actbio.2021.02.005.
52. Wan, H., Yang, X., Zhang, Y., Liu, X., Li, Y., Qin, Y., Yan, H., Gui, L., Li, K., Zhang, L., et al. (2024). Polyphenol-Reinforced Glycocalyx-Like Hydrogel Coating Induced Myocardial Regeneration and Immunomodulation. ACS Nano *18*, 21512–21522. https://doi.org/10.1021/acsnano.4c06332.
53. Fan, Y., Zhang, Y., Zhao, Q., Xie, Y., Luo, R., Yang, P., and Weng, Y. (2019). Immobilization of nano Cu-MOFs with polydopamine coating for adaptable gasotransmitter generation and copper ion delivery on cardiovascular stents. Biomaterials *204*, 36–45. https://doi.org/10.1016/j.biomaterials.2019.03.007.
54. Wang, Y., Zou, H., Dong, Z., Shi, W., Huang, J., Yang, M., Xiang, X., Xiaotong, L., Zhifeng, L., Wang, G., et al. (2025). Clearance of senescent vascular smooth muscle cells retards aging-related restenosis following bioresorbable scaffolds implantation. Biomaterials *321*, 123312. https://doi.org/10.1016/j.biomaterials.2025.123312.
55. Yao, D., He, Q., Sun, J., Cai, L., Wei, J., Cai, G., Liu, J., Lin, Y., Wang, L., and Huang, X. (2021). FGF21 attenuates hypoxia-induced dysfunction and inflammation in HPAECs via the microRNA-27b-mediated PPARγ pathway. Int. J. Mol. Med. *47*, 116. https://doi.org/10.3892/ijmm.2021.4949.
56. Han, D.W., Jung, D.Y., Park, J.C., Cho, H.H., Hyon, S.H., and Han, D.K. (2010). Underlying mechanism for suppression of vascular smooth muscle cells by green tea polyphenol EGCG released from biodegradable polymers for stent application. J. Biomed. Mater. Res. *95*, 424–433. https://doi.org/10.1002/jbm.a.32870.
57. Zhang, B., Yao, R., Li, L., Wang, Y., Luo, R., Yang, L., and Wang, Y. (2019). Green Tea Polyphenol Induced Mg(2+)-rich Multilayer Conversion Coating: Toward Enhanced Corrosion Resistance and Promoted in Situ Endothelialization of AZ31 for Potential Cardiovascular Applications. ACS Appl. Mater. Interfaces *11*, 41165–41177. https://doi.org/10.1021/acsami.9b17221.
58. Di Pietro, N., Formoso, G., and Pandolfi, A. (2016). Physiology and pathophysiology of oxLDL uptake by vascular wall cells in atherosclerosis. Vascul. Pharmacol. *84*, 1–7. https://doi.org/10.1016/j.vph.2016.05.013.
59. Natarajan, N., Florentin, J., O'Neil, S.P., Ohayon, L.L., and Dutta, P. (2022). The enigmatic role of VCAM-1 expressed by macrophages in mitochondrial metabolism and atherosclerosis. FASEB J. *36*, R6141. https://doi.org/10.1096/fasebj.2022.36.S1.R6141.
60. Li, M., Zhang, Y., Xiang, K., Su, Z., Li, X., Song, H., Wu, X., Mo, D., Ren, M., and Yang, S. (2025). Ant Colony-Inspired Adaptive Peptide Nanoregulators Remodeling the Endothelial Barrier to Alleviate Inflammatory Responses. ACS Nano *19*, 16829–16849. https://doi.org/10.1021/acsnano.5c01730.
61. Chen, S.J., Kao, Y.H., Jing, L., Chuang, Y.P., Wu, W.L., Liu, S.T., Huang, S.M., Lai, J.H., Ho, L.J., Tsai, M.C., and Lin, C.S. (2017). Epigallocatechin-3-gallate Reduces Scavenger Receptor A Expression and Foam Cell Formation in Human Macrophages. J. Agric. Food Chem. *65*, 3141–3150. https://doi.org/10.1021/acs.jafc.6b05832.
62. Zhang, H.S., Wu, T.C., Sang, W.W., and Ruan, Z. (2012). EGCG inhibits Tat-induced LTR transactivation: role of Nrf2, AKT, AMPK signaling pathway. Life Sci. *90*, 747–754. https://doi.org/10.1016/j.lfs.2012.03.013.
63. Zhang, Y., Li, L., Wang, Q., Zhan, S., Wang, L., Zhong, T., Guo, J., and Zhang, H. (2018). Fibroblast growth factor 21 induces lipolysis more efficiently than it suppresses lipogenesis in goat adipocytes. Cytotechnology *70*, 1423–1433. https://doi.org/10.1007/s10616-018-0237-1.
64. Hu, Y., Gu, J., Lin, J., Wang, Y., Yang, F., Yin, J., Yu, Z., Wu, S., Lv, H., Ji, X., and Wang, S. (2021). (-)-Epigallocatechin-3-gallate (EGCG) modulates polarized macrophages to suppress M1 phenotype and promote M2 polarization in vitro and in vivo. J. Funct.Foods *87*, 104743. https://doi.org/10.1016/j.jff.2021.104743.
65. Guo, D., Liu, H., Zhao, S., Lu, X., Wan, H., Zhao, Y., Liang, X., Zhang, A., Wu, M., Xiao, Z., et al. (2024). Synergistic rheumatoid arthritis therapy by interrupting the detrimental feedback loop to orchestrate hypoxia M1 macrophage polarization using an enzyme-catalyzed nanoplatform. Bioact. Mater. *41*, 221–238. https://doi.org/10.1016/j.bioactmat.2024.07.026.
66. Liu, Z., Liu, Z., Shan, Y., Xu, C., Yu, B., and Xu, F.-J. (2023). ROS-scavenging anti-inflammatory nucleic acid delivery system for targeted treatment of atherosclerosis. Nano Today *53*, 102005. https://doi.org/10.1016/j.nantod.2023.102005.
67. Xiang, L., Wang, G., Zhuang, Y., Luo, L., Yan, J., Zhang, H., Li, X., Xie, C., He, Q., Peng, Y., et al. (2025). Safety and efficacy of GLP-1/FGF21 dual agonist HEC88473 in MASLD and T2DM: A randomized, double-blind, placebo-controlled study. J. Hepatol. *82*, 967–978. https://doi.org/10.1016/j.jhep.2024.12.006.

STAR★METHODS

KEY RESOURCES TABLE

REAGENT or RESOURCE	SOURCE	IDENTIFIER
Antibodies		
PE anti-mouse CD86 Antibody	BioLegend	Cat # 105008; RRID: AB_313162
APC anti-mouse CD206 (MMR) Antibody	BioLegend	Cat # 141708; RRID: AB_2562341
PerCP/Cyanine5.5 anti-mouse/human CD11b Antibody	BioLegend	Cat # 101228; RRID: AB_2129537
Alexa Fluor® 488 anti-mouse F4/80 Antibody	BioLegend	Cat # 123120; RRID: AB_893506
TruStain FcX^{TM} (anti-mouse CD16/32) Antibody	BioLegend	Cat # 101320; RRID: AB_1574973
Alexa Fluor® 488 anti-mouse F4/80 Antibody	BioLegend	Cat # 123119; RRID: AB_893495
CD31 Polyclonal antibody	Proteintech	Cat # 11265-1-AP; RRID: AB_2299349
CD68 Polyclonal antibody	Proteintech	Cat # 25747-1-AP RRID: AB_2881384
Alpha smooth muscle actin Polyclonal antibody	Proteintech	Cat # 14395-1-AP; RRID: AB_2252190
Phospho-ERK1/2 (Thr202/Tyr204) Recombinant monoclonal antibody	Proteintech	Cat # 80031-1-RR; RRID: AB_2881385
ERK1/2 Polyclonal antibody	Proteintech	Cat # 11257-1-AP; RRID: AB_2139685
Phospho-AKT (Ser473) Monoclonal antibody	Proteintech	Cat # 66444-1-Ig; RRID: AB_2881386
AKT Polyclonal antibody	Proteintech	Cat # 10176-2-AP; RRID: AB_2107855
Cyclin D1 Monoclonal antibody	Proteintech	Cat # 60186-1-Ig; RRID: AB_10733222
PCNA Monoclonal antibody	Proteintech	Cat # 60097-1-Ig; RRID: AB_2169916
MMP2 Polyclonal antibody	Proteintech	Cat # 10373-2-AP; RRID: AB_2144367
ROCK1 Polyclonal antibody	Proteintech	Cat # 21850-1-AP; RRID: AB_2881387
RHOA Polyclonal antibody	Proteintech	Cat # 10749-1-AP RRID: AB_2285104
Alpha smooth muscle actin Polyclonal antibody	Proteintech	Cat # 14395-1-AP; RRID: AB_2252190
Beta Actin Polyclonal antibody	Proteintech	Cat # 20536-1-AP; RRID: AB_10733223
PPAR Gamma Polyclonal antibody	Proteintech	Cat # 16643-1-AP; RRID: AB_2881389
eNOS Polyclonal antibody	Proteintech	Cat # 27120-1-AP RRID: AB_2880764
PGC1a Monoclonal antibody	Proteintech	Cat # 66369-1-Ig RRID: AB_2919318
NRF2/NFE2L2 Polyclonal antibody	Proteintech	Cat # 16396-1-AP; RRID: AB_2881393
P21 Polyclonal antibody	Proteintech	Cat # 27296-1-AP; RRID: AB_2881394
VEGFA Polyclonal antibody	Proteintech	Cat # 19003-1-AP; RRID: AB_2212607
Anti-FGFR1 antibody	Abcam	Cat # ab10646; RRID: AB_297221
Chemicals, peptides, and recombinant proteins		
$KMnO_4$	Sigma-Aldrich	Cat # 238511
Epigallocatechin gallate (EGCG)	Yuanye	Cat # B20106
Cysteine (Cys)	Yuanye	Cat # S31097
1-ethyl-(3-dimethylaminopropyl) carbodiimide hydrochloride (EDC)	Sigma-Aldrich	Cat # E7750
N-hydroxysuccinimide (NHS)	Sigma-Aldrich	Cat # 130672

(*Continued on next page*)

Continued		
REAGENT or RESOURCE	SOURCE	IDENTIFIER
Fibroblast growth factor 21 (FGF21), recombinant mouse-derived renin (HEK293, His)	MedChemExpress	Cat # HY-P72651
Cholesterol	Aladdin	Cat # C104028
Dipalmitoylphosphatidylcholine (DPPC)	Aladdin	Cat # D130424
1,2-distearoyl-*sn*-glycero-3-phosphoethanolamine-N-[amino(polyethylene glycol)-2000] (DSPE-PEG2K)	MedChemExpress	Cat # HY-142979
DSPE-PEG2K-maleimide (MAL)	MedChemExpress	Cat # HY-140739
VHPK peptide	ChinaPeptides (QYAOBIO)	N/A
Tris(2-carboxyethyl)phosphine (TCEP)	Aladdin	Cat # T107252
Dulbecco's modified Eagle's medium (DMEM)	Gibco	Cat # 10566016
Roswell Park Memorial Institute (RPMI) 1640	Gibco	Cat # 31870082
Fetal bovine serum (FBS)	Gibco	Cat # A3160901
Penicillin-streptomycin	Gibco	Cat # 15640055
Rhodamine 123 staining	beyotime	Cat # C2007
S-nitroso-N-acetyl-penicillamine	MedChemExpress	Cat # HY-121526
Lipopolysaccharide (LPS)	MedChemExpress	Cat # HY-D1056
Paraformaldehyde (PFA)	MedChemExpress	Cat # HY-DY3003
DCFH-DA	beyotime	Cat # S1105S
Interleukin 4 (IL4)	procell	Cat # PCK343
Oil Red O Staining Kit	beyotime	Cat # C0157S
Ox-LDL	yeasen	Cat # 20605ES05
EDTA	Gibco	Cat # 25300054
Critical commercial assays		
Mouse TNF-α ELISA kit	BioLegend	Cat # 430904
Mouse IL-6 ELISA kit	BioLegend	Cat # 431304
Mouse IL-1β ELISA kit	BioLegend	Cat # 432604
cGMP ELISA kit	Abcam	Cat # ab285321
Cell Counting Kit-8 (CCK-8) assay kit	Beyotime	Cat # C0038
GAPDH qPCR primer kit	ChemicalBook	Cat # AP4048
RevertAid First Strand cDNA Synthesis Kit	Thermo Scientific	Cat # K1622
H_2O_2 assay kit	Beyotime	Cat # S0038
Deposited data		
RNA-sequencing	This paper	NGDC BioProject (BIG Sub): PRJCA063571
Experimental models: Cell lines		
RAW 264.7 Cells	American Type Culture Collection	Cat # TIB-71
Mouse aortic smooth muscle cells (MOVAS)	American Type Culture Collection	Cat # CRL-2797
Experimental models: Organisms/strains		
Apolipoprotein E (*Apoe*)$^{-/-}$ mice	Hangzhou Hangsi Biotechnology	N/A
New Zealand white rabbits	Hangzhou Hangsi Biotechnology	N/A
Rag1/Il2rg Double KO rabbits	GemPharmatech	N/A
Sprague–Dawley rats	Hangzhou Hangsi Biotechnology	N/A
Miniature potbellied pigs	Hangzhou Hangsi Biotechnology	N/A

(*Continued on next page*)

CellPress
OPEN ACCESS

Continued

REAGENT or RESOURCE	SOURCE	IDENTIFIER
Oligonucleotides		
ABCA1 Forward 5′ ACCCACCCTATGAACAACATGA	This paper	N/A
ABCA1 Reverse 5′ GAGTCGGGTAACGGAAACAGG	This paper	N/A
ABCG1 Forward 5′ ATTCAGGGACCTTTCCTATTCGG	This paper	N/A
ABCG1 Reverse 5′ CTCACCACTATTGAACTTCCCG	This paper	N/A
ARG1 Forward 5′ GTGGAAACTTGCATGGACAAC	This paper	N/A
ARG1 Reverse 5′ AATCCTGGCACATCGGGAATC	This paper	N/A
INOS Forward 5′ CTCGCTGCATCGGCAGGATTC	This paper	N/A
INOS Reverse 5′ AGTCATGCTTCCCATCGCTCC	This paper	N/A
ERK/MAPK1 Forward 5′ TACACCAACCTCTCGTACATCG	This paper	N/A
ERK/MAPK1 Reverse 5′ CATGTCTGAAGCGCAGTAAGATT	This paper	N/A
AKT1 Forward 5′ AGCGACGTGGCTATTGTGAAG	This paper	N/A
AKT1 Reverse 5′ GCCATCATTCTTGAGGAGGAAGT	This paper	N/A
CyclinD1 Forward 5′ GCTGCGAAGTGGAAACCATC	This paper	N/A
CyclinD1 Reverse 5′ CCTCCTTCTGCACACATTTGAA	This paper	N/A
PCNA Forward 5′ CCTGCTGGGATATTAGCTCCA	This paper	N/A
PCNA Reverse 5′ CAGCGGTAGGTGTCGAAGC	This paper	N/A
MMP2 Forward 5′ TACAGGATCATTGGCTACACACC	This paper	N/A
MMP2 Reverse 5′ GGTCACATCGCTCCAGACT	This paper	N/A
ROCK1 Forward 5′ AACATGCTGCTGGATAAATCTGG	This paper	N/A
ROCK1 Reverse 5′ TGTATCACATCGTACCATGCCT	This paper	N/A
RHOA Forward 5′ AGCCTGTGGAAAGACATGCTT	This paper	N/A
RHOA Reverse 5′ TCAAACACTGTGGGCACATAC	This paper	N/A
ACTA2 Forward 5′ AAAAGACAGCTACGTGGGTGA	This paper	N/A
ACTA2 Reverse 5′ GCCATGTTCTATCGGGTACTTC	This paper	N/A
FGFR1 Forward 5′ TAATACCACCGACAAGGAAATGG	This paper	N/A
FGFR1 Reverse 5′ TGATGGGAGAGTCCGATAGAGT	This paper	N/A

(*Continued on next page*)

Continued

REAGENT or RESOURCE	SOURCE	IDENTIFIER
PPARγ Forward 5′ AGAGCCCCATCTGTCCTCTC	This paper	N/A
PPARγ Reverse 5′ ACTGGTAGTCTGCAAAACCAAA	This paper	N/A
eNOS Forward 5′ GGCTGGGTTTAGGGCTGTG	This paper	N/A
eNOS Reverse 5′ CTGAGGGTGTCGTAGGTGATG	This paper	N/A
PGC1α Forward 5′ TATGGAGTGACATAGAGTGTGCT	This paper	N/A
PGC1α Reverse 5′ CCACTTCAATCCACCCAGAAAG	This paper	N/A
Nrf2 Forward 5′ TCTTGGAGTAAGTCGAGAAGTGT	This paper	N/A
Nrf2 Reverse 5′ GTTGAAACTGAGCGAAAAAGGC	This paper	N/A
HO-1 Forward 5′ AAGCCGAGAATGCTGAGTTCA	This paper	N/A
HO-1 Reverse 5′ GCCGTGTAGATATGGTACAAGGA	This paper	N/A
VEGFA Forward 5′ CTGCCGTCCGATTGAGACC	This paper	N/A
VEGFA Reverse 5′ CCCCTCCTTGTACCACTGTC	This paper	N/A
KLF2 Forward 5′ CTCAGCGAGCCTATCTTGCC	This paper	N/A
KLF2 Reverse 5′ CACGTTGTTTAGGTCCTCATCC	This paper	N/A
Software and algorithms		
GraphPad Prism 9.0	GraphPad Software	https://www.graphpad.com/
BioRender	BioRender	https://www.biorender.com/
ImageJ 1.8.0	ImageJ	https://imagej.net/ij/
FlowJo	FlowJo	https://www.flowjo.cn/
RadiAnt DICOM Viewer 2020.2.3	RadiAnt DICOM Viewer	https://www.radiantviewer.com/
Living Image 4.3.1	PerkinElmer	https://www.perkinelmer.com.cn/
Origin8.5	originlab	https://www.originlab.com/
SPSS 11.5	IBM Corp	https://www.ibm.com/cn-zh/products/spss

EXPERIMENTAL MODEL AND STUDY PARTICIPANT DETAILS

Cells

Mouse aortic vascular smooth muscle cells (MOVAS) and primary human umbilical vein endothelial cells (HUVECs) were cultured in DMEM supplemented with 10% fetal bovine serum (FBS), 100 U/mL penicillin, and 100 μg/mL streptomycin. RAW264.7 murine macrophages were cultured in RPMI-1640 medium supplemented with 10% FBS, 100 U/mL penicillin, and 100 μg/mL streptomycin. All cells were maintained at 37°C in a humidified incubator containing 5% CO_2.

MOVAS and RAW264.7 cells were obtained from authenticated commercial cell repositories. Cell identity was verified by the supplier, and all cultures were routinely tested for mycoplasma contamination before use, with negative results. HUVECs were purchased from the American Type Culture Collection (ATCC, CRL-1730) as primary endothelial cells and were authenticated by the supplier. All HUVEC cultures were routinely tested for mycoplasma contamination before use, with negative results.

Foam cell models were established from MOVAS and RAW264.7 cells by incubation with 50 μg/mL oxidized low-density lipoprotein (oxLDL) for 48 h. Briefly, cells in the logarithmic growth phase were seeded into six-well plates at a density of 5×10^4 cells/mL per

well before oxLDL treatment. This protocol successfully established vascular smooth muscle cell-derived and macrophage-derived foam cell models.

Animal models

All animal experiments and *in vivo* procedures were conducted in accordance with the Guide for the Care and Use of Laboratory Animals and were approved by the Medical Ethics Committee of Zhejiang Province (approval no. xmsq2024-0005) and the Experimental Animal Center of Lishui Central Hospital (approval no. 2025D042). Apoe$^{-/-}$ mice (male, 6–8 weeks old, 15–20 g), Sprague–Dawley rats (male, 6–8 weeks old, 250–300 g), New Zealand White rabbits (male, 3–4 months old, 2.5–3.0 kg), miniature potbellied pigs (male, 5–6 months old, approximately 35 kg), and Rag1$^{-/-}$Il2rg$^{-/-}$ double-knockout rabbits (male, 3–4 months old, 2.5–3.0 kg) were purchased from Hangzhou Hangsi Biotechnology Co., Ltd. (Apoe$^{-/-}$ mice, Sprague–Dawley rats, New Zealand White rabbits, and miniature potbellied pigs) or GemPharmatech Co., Ltd. (Rag1$^{-/-}$Il2rg$^{-/-}$ double-knockout rabbits). All animals were maintained under specific pathogen-free (SPF) conditions at controlled temperature and humidity under a 12 h light/12 h dark cycle. Mice and rats were group-housed (up to five animals per cage), whereas rabbits and pigs were housed individually. Food and water were provided *ad libitum*.

Sex as a biological variable

Only male animals were used in the *in vivo* experiments to reduce biological variability associated with hormonal cycling and to maintain consistency across atherosclerosis and stent-implantation models. Therefore, the influence of sex on the therapeutic efficacy and biosafety of the EGCG-Cys-MFVL-WE43 stent was not systematically evaluated. Gender was not applicable because no human participants were included in this study. The lack of sex-based comparative analysis is acknowledged as a limitation of this study.

METHOD DETAILS

Preparation of the EGCG-Cys nanocoating

Given the different variability in data collection methods, polylactic acid (PLA) sheets, silicon wafers, WE43 sheets, and WE43 stents were selected for the surface modification study. Notably, the construction procedure for the nanocoating was identical on all substrates. For example, before fabricating the EGCG-Cys nanocoatings, PLA sheets were ultrasonically cleaned with acetone and ultrapure (UP) water in sequence (10 min × 3 times) and then dried with N_2 for 2 h before use. Next, the PLA sheets were immersed in Dopa solution (2 mg/mL in a Tris buffer solution [pH 8.5]) for 2 h, followed by cleaning with UP water (3 min × 3 times). Then, the Tris buffer solution (10 mM, pH 8.5) was prepared, and EGCG and Cys were added (final concentration of 0.2 mg/mL). The prepared solution was placed in an ultrasonic shaker for 3 min to ensure that all solutes were evenly dispersed and fully dissolved. After 12 h of magnetic stirring at room temperature, the nanoparticles were collected using a high-speed centrifuge at 13,000 rpm. The supernatant was removed, the precipitate was washed with reverse osmosis (RO) water using ultrasound, and the nanoparticles were collected using a high-speed centrifuge; this process was repeated three times. Then, the collected nanoparticles were dried in a freeze-drying machine to obtain EGCG-Cys nanoparticles. Briefly, the EGCG-Cys nanoparticles (0.2 mg/mL) were suspended in a Tris buffer solution (10 mM, pH 8.5), and the EGCG-Cys-treated samples were then immersed in this solution for 1 h.

Preparation of the MnO_2 NFs

First, 80 mg of potassium permanganate ($KMnO_4$) was dissolved in 40 mL of hydrochloric acid (0.1 mol/L) to obtain a uniform wine-colored solution. Next, 1 mL of citric acid (100 mM) was added to the solution, and it was stirred at room temperature for 30 min, causing a brown suspension to appear. Then, the product (MnO_2 NFs) was centrifuged, washed several times by PBS, and dried in a vacuum at −50°C. Next, a certain amount of MnO_2 NFs (0.5 mg/mL, 1 mL) was weighed and adjusted to pH 6.0 using potassium carbonate (K_2CO_3) under vigorous ultrasonic treatment for 1 h. Then, the MnO_2 NFs were activated by gentle shaking with EDC (2 mg/mL) and NHS (4 mg/mL) for 30 min. Next, the mixture was centrifuged and washed three times before use. Then, the activated MnO_2 NFs were incubated with FGF21 (100 μg/mL, 0.5 mL) in phosphate-buffered saline (PBS) at 37°C for 1 h and then centrifuged three times to remove unbound components. Finally, the product was inactivated by incubation with 1% bovine serum albumin (BSA) for 30 min, dispersed in 1 mL of PBS, and then stored at 4°C until needed.

Preparation of the MnO_2 NF-FGF21@VHPK-Liposomes

To prepare the MnO_2 NF-FGF21@VHPK-Liposomes (MFVLs), MnO_2 NF-FGF21 nanoparticles were first synthesized by dissolving MnO_2 NFs and FGF21 in double-distilled water (ddH_2O, 0.2 mg/mL each), followed by ultrasonic dispersion, magnetic stirring for 12 h, and high-speed centrifugation to collect and wash the MnO_2 NF-FGF21 nanoparticles, which were then dried using a freeze-dryer.

To prepare the liposomes, cholesterol, dipalmitoylphosphatidylcholine (DPPC), 1,2-distearoyl-*sn*-glycero-3-phosphoethanolamine-N-[amino(polyethylene glycol)-2000] (DSPE-PEG2K), and DSPE-PEG2K-maleimide (MAL) were first dissolved in chloroform. Next, a thin film was formed via rotary evaporation, hydrated with ddH_2O, and dispersed using ultrasound. Then, the MnO_2 NF-FGF21 nanoparticles were incorporated into the liposomes by adding them during the hydration step. Finally, the VHPK peptide was activated using tris(2-carboxyethyl)phosphine (TCEP) in PBS, mixed with the liposome solution, and left to stand to form MFVLs.

The morphology, size distribution, and zeta potential of the nanoparticles were examined using transmission electron microscopy (TEM) and a laser particle size analyzer. The nanoparticles' cholesterol and ROS scavenging efficiency were assessed using a microplate reader. The reaction process and key functional groups of the nanoparticles were analyzed using Fourier transform infrared (FTIR) spectroscopy.

Preparation of the EGCG-Cys-MFVL nanocoating

The EGCG-Cys-MFVL nanocoating was prepared by immobilizing MFVL liposomes onto the preformed EGCG-Cys nanocoating through a combination of chemical infiltration and electrostatic adsorption. Briefly, the MFVL liposomes were dispersed in Tris buffer (10 mM, pH 8.5), and the EC-coated substrate was immersed in the liposomal suspension under constant agitation for 12 h. After incubation, the substrate was thoroughly rinsed to remove unbound liposomes and subsequently lyophilized to obtain the drug-loaded sample.

Characterization of the EGCG-Cys-MFVL nanocoating

The surface morphology of the resulting nanocoating was characterized using SEM and AFM. AFM was also employed to analyze the surface roughness of the nanocoating. Given that the hydrophilicity/hydrophobicity of implantable devices significantly influences cell adhesion and proliferation during service, the wettability of the coated surface was evaluated by measuring the WCA at room temperature using a contact angle/surface tension analyzer (Dataphysics OCA20).

To evaluate the stability of the drug-loaded stent coating under dynamic flow conditions, an *in vitro* flow erosion model was established and the residual coating/drug signal on the stent surface was assessed under different wall shear stress levels. Stent samples were fixed inside a flow circuit with an inner diameter of 2.5 mm, and PBS (pH 7.4) was used as the circulating medium at 37°C. The wall shear stress was calculated according to the laminar flow equation for a cylindrical tube:

$$\tau = \frac{4\mu Q}{\pi R^3}$$

Where τ is the wall shear stress, $\mu$is the dynamic viscosity of the perfusion medium, $Q$is the volumetric flow rate, and $R$is the inner radius of the flow channel. The dynamic viscosity of PBS at 37°C was set as 0.001 Pa·s, and the inner radius of the tubing was 1.25 mm. Based on this equation, the flow rates were adjusted to 37.56, 75.13, and 150.26 mL/min, corresponding to wall shear stresses of 0.5, 1.0, and 2.0 Pa, respectively.

Stents were continuously perfused under the designated shear stress conditions, and samples were collected at predetermined time points. The residual fluorescence intensity on the stent surface was quantified using a fluorescence imaging system. The coating/drug retention was normalized to the initial fluorescence intensity at day 0 and expressed as:

$$\text{Residual fluorescence intensity } (\%) = \frac{I_t}{I_0} \times 100\%$$

Where I_0represents the initial fluorescence intensity and $I_t$represents the fluorescence intensity at each time point. At least three independent samples were included in each group. Data were presented as mean ± SD.

Radial support testing

To reproduce realistic compression conditions, smooth hard contact was defined between the stent and rigid plates with a penalty factor of 0.2. The loading protocol consisted of two stages.

(1) Planar compression: Radial displacement was applied using 12 rigid planar plates. For stents of different nominal diameters, the diameter was reduced by 50% of the initial value at a constant compression rate of 0.2 mm/min. All translational and rotational degrees of freedom of the plates were fixed except in the radial direction, allowing only inward motion. Circumferential constraints were imposed at both stent ends to suppress rotation, while an axial constraint at the mid-length prevented rigid-body axial displacement.
(2) Unloading: The applied radial displacement was removed (r = 0), permitting elastic recovery of the stent as the plates returned to their initial positions. The load–displacement response was recorded continuously to obtain the radial support force. To enable comparison among stents of different lengths, radial support force was normalized to the radial support strength, and the expression is

$$P_{Load} = F/L_{crimp} \quad \text{(Equation 1)}$$

Where: P_{Load}–radial support strength, F-radial support force, L_{crimp}– radial compression distance.

ROS response behavior

The WE43 samples coated with varying MFVL:EGCG-Cys ratios (3:1, 2:2, and 1:3) were immersed in an H_2O_2 solution (400 μM) and allowed to react for 4 h at room temperature. During incubation, the nanocoatings catalytically decomposed H_2O_2 in the supernatant.

Next, the residual H_2O_2 concentration was quantified using a commercial H_2O_2 assay kit (Beyotime Biotechnology) according to the manufacturer's protocol. The absorbance was measured at the recommended wavelength, and the H_2O_2 consumption efficiency was calculated based on the standard curve.

Concentration-dependent H_2O_2 hydrolysis

Different concentrations of H_2O_2 (0.01, 0.1, 1, 2, 3 mM; 450 μL) were placed in a 1.5 mL Eppendorf tube, and then a EGCG-Cys-MFVL-WE43 stent was added and left in the solution for 0, 2, 5, 8, 10, 15, 20, 25, and 30 min. Next, 50 μL of each reaction mixture was transferred to a 96-well plate ($n = 3$), and a mixture of ABTS (0.09 mg/mL) and HRP (0.02 mg/mL) was added to each well (150 μL). After 5 min, the absorbance at 800 nm was measured.

In vitro functional evaluation of HUVECs, macrophages and SMCs

The growth behavior of endothelial cells on different surfaces was assessed using human umbilical vein endothelial cells (HUVECs) from ATCC. EGCG-Cys and EGCG-Cys-MFVL nanocoatings were deposited on PLA substrates and in 96-well plates. Next, sterile samples ($n = 6$) were cultured with HUVECs (2.5×10^4 cells/mL) to a stable state for 1 and 3 days. To simulate the effect of ROS on HUVEC growth post-implantation, 300 μM hydrogen peroxide (H_2O_2) was added to the sample medium ($n = 3$) after 12 or 60 h of culturing and further cultured for 12 h. The activity, adhesion, and morphology of adherent HUVECs secreted into the medium were assessed using the CCK-8 assay, rhodamine 123 staining, 4′,6-diamidino-2-phenylindole (DAPI) staining, tetramethylrhodamine isothiocyanate (TRITC)-phalloidin staining, and Griess reagent.

Murine RAW264.7 macrophages were used to evaluate the inflammatory regulation ability of the samples *in vitro*. The effects of nonspecific protein adsorption and ROS post-implantation were simulated by pre-incubation in whole-plasma murine protein for 24 h or by adding 50 μM of H_2O_2 to the medium. The control (Ctrl) samples received no plasma or H_2O_2. All samples were cultured with macrophages at a density of 5×10^4 cells/mL for 1 or 3 days. The absorbance (optical density at 450 nm [OD_{450}]) and activation state of adherent macrophages were evaluated using the CCK-8 assay and TRITC-phalloidin staining. Enzyme-linked immunosorbent assay (ELISA) kits were used to detect the concentrations of the inflammatory factors tumor necrosis factor-alpha (TNF-α), interleukin 6 (IL6), and interleukin 1 beta (IL1B) in the medium. In addition, adherent macrophages were collected, and their RNA was extracted and reverse transcribed. The gene expression of *Il6* and *Tnf-α* was detected using real-time quantitative polymerase chain reactions, with their relative expression levels normalized to glyceraldehyde-3-phosphate dehydrogenase (*GAPDH*).

Umbilical artery SMCs were used to evaluate the ability of the nanocoatings to inhibit SMC hyperproliferation. Steady-state SMCs were cultured with the samples after aseptic treatment. A cell density of 5×10^4 cells/mL was used for the PLA substrates and 48-well plates, and a cell density of 5×10^5 cells/mL was used for the six-well plates. S-nitroso-N-acetyl-penicillamine and glutathione were added at certain times according to the experimental design. The viability and cGMP contents of the SMCs were measured using CCK-8 and cGMP ELISA kits, respectively. The morphology of SMCs was observed using TRITC-phalloidin staining under a confocal laser scanning microscope (CLSM).

ROS scavenging

After successfully inducing various types of foam cells, the cells were fixed with 4% PFA for 15 min, followed by washing twice with PBS. Next, the cells were stained with DCFH-DA (0.01 μM, 1 mL) for 20 min at 37°C in a cell culture incubator. Then, the cells were washed three times with serum-free medium to completely remove unbound DCFH-DA. Finally, the cells were mounted with a DAPI-containing mounting medium, and the fluorescence signals were recorded under a CSLM. Arterial slices from the mice, prepared using the above method, were stained with 1 mL of DCFH-DA (10 μM) at a 1:1000 dilution for 20 min. The slices were medium containing DAPI. The CLSM pictures were recorded on a CLSM (TCS SP8, Leica, Germany).

Evaluation of endothelial cell and VSMC migration behavior

The migratory capacities of HUVECs and MOVAS were evaluated using a standardized scratch wound healing assay. Briefly, cells were cultured to form confluent monolayers in culture dishes. Upon reaching >90% confluence, a uniform linear scratch was created in the monolayer using a sterile 200 μL pipette tip. The dislodged cells were gently removed by washing with culture medium to maintain a clear wound boundary. The dishes were then replenished with fresh medium containing the test extracts and incubated at 37°C under 5% CO_2. Cell migration into the wound area was monitored and digitally documented at 24 and 72 h post-scratching using phase-contrast microscopy. Their migratory capability was quantitatively analyzed by measuring the relative wound closure percentage through comparison of the denuded area at different time points. The clonogenic potential of HUVECs and MOVAS VSMCs was evaluated by seeding 10^3 cells/well in six-well plates, culturing them with test extract-containing media (replaced every 3 days) for 14 days, followed by methanol fixation and 0.5% crystal violet staining to quantify colony formation (>50 cells/colony) as a measure of long-term proliferative capacity under experimental conditions.

Cellular uptake analysis

RAW264.7 macrophages and MOVAS were seeded in a six-well plate at a density of 5×10^4 cells/well and induced with the polarization agent oxLDL (100 ng/mL) for 24 h. Next, the supernatant was aspirated, and the cells were washed three times with PBS.

Then, the cells were fixed with 4% PFA for 15 min and washed twice with PBS. Finally, the cells were mounted using a mounting medium containing DAPI. The images were recorded on a CLSM (TCS SP8, Leica).

Flow cytometry analysis of M1 and M2 polarization

To investigate the effect of various components of the coated stent on the proportion of immune cells in atherosclerotic plaques, murine RAW264.7 macrophages were cultured with each component. Briefly, RAW264.7 murine macrophages were seeded in a six-well plate and incubated for 24 h before each component was added. After 24 h, the cells were collected and incubated with the TruStain FcX (anti-mouse CD16/32) antibody (1 μg/test; BioLegend), which blocks non-specific binding to Fc receptors, for 5–10 min at 4°C. Next, the cells were stained with AF488-conjugated anti-mouse F4/80 (1 μg/test; cat. no. 123119, BioLegend), PerCP-Cy5.5 anti-mouse CD11b (0.25 μg/test; cat. no. 101227, BioLegend), and PE anti-mouse CD86 (1 μg/test; cat. no. 105007, BioLegend) antibodies at 4°C for 30 min. Then, the cells were washed 2–3 times with PBS and fixed with cell fixation buffer (500 μL, BioLegend) for 30 min at room temperature in the dark. Next, the fixative was discarded after centrifugation at 50*g* for 5 min, and the cells were resuspended in 2 mL of 10× intracellular staining permeabilization wash buffer (BioLegend) diluted 1:10 with ddH_2O. Then, the supernatant was discarded after centrifugation at 150*g* for 5 min, and the above steps were repeated 2–3 times. Next, the cells were resuspended in 100 μL of 1× intracellular staining permeabilization wash buffer. Then, the CD206 antibody (0.5 μg/test; cat. no. 141707, BioLegend) was added and incubated in the dark at room temperature for 30 min. After incubation, the cells were washed 2–3 times with 2 mL of 1× intracellular staining permeabilization wash buffer, resuspended in 500 μL of cell staining buffer, and placed on the flow cytometer. Finally, the data were analyzed using FlowJo software (Tree Star Inc.).

Western blotting

RIPA lysis buffer (RIPA: PMSF: phosphatase inhibitor = 100:1:1) was added to the centrifuged cell pellet. Next, the mixture was placed on ice and vortexed for 30 s every 10 min; this process was repeated three times to ensure complete cell lysis. Then, the lysate was centrifuged at 4°C and 12,000 rpm for 10 min in a low-temperature centrifuge to collect the protein supernatant. Next, the protein concentration in the samples was determined using the bicinchoninic acid method, and the protein loading amount was normalized to 50–100 μg in a total volume of 20 μL. The 10% separating gel consisted of H_2O (8.0 mL), 30% acrylamide (6.6 mL), 1.5 M Tris buffer (pH 8.8, 5 mL), 10% ammonium persulfate (200 μL), 10% sodium dodecyl sulfate (SDS, 200 μL), and N,N,N′,N′-tetramethylethylenediamine (TEMED, 8 μL). The 5% stacking gel consisted of H_2O (4.1 mL), 30% acrylamide (1 mL), 1 M Tris buffer (pH 6.8, 750 μL), 10% ammonium persulfate (60 μL), 10% SDS (60 μL), and TEMED (6 μL). Protein samples (50–100 μg, 20 μL) were loaded into the wells. The gel was initially run at 80 V for the stacking gel, and once the marker was separated, the voltage was increased to 120 V until the tracking dye reached the bottom of the gel, at which point electrophoresis was stopped.

The proteins in the gel were transferred to a polyvinylidene difluoride (PVDF) membrane (0.22 μm) using a wet transfer system at 300 mA for 1.5 h. Next, the PVDF membrane was blocked with a 5% skim milk solution (2.5 g in 50 mL of Tris-buffered saline containing Tween 20 [TBST]) to reduce non-specific binding. Then, the PVDF membrane was incubated at room temperature on a shaker (60 rpm) for 1∼1.5 h. Next, the blocking solution was removed, and the PVDF membrane was washed three times with TBST. The membrane was then incubated with primary antibodies (target protein antibody, diluted 1:1000) overnight at 4°C. Next, the primary antibody was removed, and the PVDF membrane was washed three times with TBST. Then, it was incubated with a secondary antibody specific to the primary antibody species (mouse, rat, or rabbit, 1:10,000 dilution) at room temperature on a shaker (60 rpm) for 30–60 min. Next, the PVDF membrane was washed three times with TBST for 5 min to remove unbound secondary antibodies. Finally, a highly sensitive enhanced chemiluminescence detection reagent was prepared and applied uniformly to the membrane. The results were semi-quantitatively analyzed using ImageJ software (version 1.46r).

Quantitative real-time PCR (qPCR)

Total RNA was extracted from cells using RNA-Quick Purification kit (ES Science, Cat. RN001). Genomic DNA was eliminated by DNase I (Thermo Scientific, EN0521) treatment. RNA concentration and purity were determined using a NanoDrop 2000 spectrophotometer, and samples with an A260/A280 ratio between 1.9 and 2.1 were used for subsequent experiments. Complementary DNA (cDNA) was synthesized from 1 μg of total RNA using the RevertAid First Strand cDNA Synthesis Kit (Thermo Scientific, K1622) and then diluted 5-fold with nuclease-free water and stored at −20°C. Primers were synthesized by Sangon Biotech (Shanghai, China). Primer specificity and amplification efficiency (95%–105%) were validated by melting curve analysis (single peak) and standard curves generated from serial dilutions of cDNA. qPCR was performed on a CFX96 Real-Time PCR Detection System (Bio-Rad) using SYBR Green Master Mix (Vazyme, Q711-02). The 20 μL reaction mixture contained 10 μL of 2× Master Mix, 0.8 μL each of forward and reverse primers (10 μM), 2 μL of cDNA template, and 6.4 μL of ddH_2O. The thermal cycling conditions were as follows: initial denaturation at 95°C for 3 min, followed by 40 cycles of denaturation at 95°C for 15 s and annealing/extension at 60°C for 30 s. A melting curve analysis was performed from 60°C to 95°C after amplification. Each sample was run in triplicate, and GAPDH was used as an internal reference gene. Relative gene expression levels were calculated using the $2^{-\Delta\Delta CT}$ method.

Oil Red O (ORO) staining

The cells were seeded at a density of 5×10^5 cells/well in six-well plates. Once adhered, they were incubated for 24 h with the polarization inducers lipopolysaccharide (LPS, 100 ng/mL) for M1 polarization or interleukin 4 (IL4, 20 ng/mL) for M2 polarization. Next,

CellPress
OPEN ACCESS

the cells were fixed with 4% paraformaldehyde (PFA) for 15 min, stained with ORO solution for 10–20 min, and then briefly washed with PBS for 20 s. Then, sufficient PBS was added to cover the cells completely. Finally, the stained cells were observed and imaged using an inverted fluorescence microscope.

After one month of treatment, mice were sacrificed and fixed in the supine position. Then, their thoracic cavity was opened immediately and perfused with saline through the left ventricle for 2 min. Next, the whole aorta from the heart to the branch of the inferior iliac artery was quickly separated and stored in 10% neutral buffered formalin before detection. The atherosclerotic lesions were observed by removing the aorta from the 4% PFA fixation solution, rinsing it three times with PBS, and then cutting and longitudinally opening it or preparing 5-μm-thick slices. The aortas were stained with ORO to quantify atherosclerotic lesions. The percentage of the ORO^+ area was measured using ImageJ software (version 1.8.0, US National Institutes of Health).

Mice *in vivo* treatment

Following 1 month of high-fat diet induction, the mice were administered weekly tail vein injections (0.1 mL, 160 μg/mL) of either PBS (negative control), VHPK-Liposomes, FGF21, MnO_2 NFs@VHPK-Liposomes, or MFVL for 4 consecutive weeks. Thereafter, the mice received the same treatments via tail vein injection once per month for an additional 12 months. After the treatment period, the mice were euthanized, and their aortas were isolated for *ex vivo* evaluation of early atherosclerotic lesions. Plaque burden was quantitatively assessed by measuring lesion size, frequency, and spatial distribution along the arterial tree to determine the therapeutic efficacy of MFVLs in attenuating AS progression.

High-throughput sequencing and bioinformatics analysis of the atherosclerotic microenvironment *in vivo* and *in vitro*

To investigate the effects of EGCG-Cys-MnO_2 NF-FGF21@VHPK-Liposomes on the signaling pathways involved in the remodeling of the inflammatory immune microenvironment in AS, next-generation RNA sequencing was used to identify differentially expressed genes related to macrophage polarization. Transcriptomic analyses were performed on rat carotid artery tissues following stent implantation, comparing arteries treated with EGCG-Cys-MFVL-coated stents (EGCG-Cys-MFVL group) against those receiving bare metal stents (Ctrl group) or non-stented controls. WGCNA was employed to identify key gene modules associated with vascular responses. Differentially expressed immune-related genes were visualized using a hierarchical clustering heatmap. Functional enrichment analysis using Metascape was used to assess the involvement of cytokine-mediated signaling pathways and immune regulatory processes, providing mechanistic insights into the host response to EGCG-Cys-MFVL-coated stents.

Blood fluorescence

The mice were administered indocyanine green (ICG)-conjugated MnO_2 NF-FGF21@Liposomes (MFL) or ICG-conjugated MFVLs via their tail vein at different time points to establish a time gradient of 0.5, 2, 6, 12, and 24 h. Then, orbital blood was collected, placed in a 12-well plate, and fluorescence images were acquired in the near-infrared (NIR)-II range.

Aortic fluorescence

The mice were administered ICG-conjugated MnO_2 NF-FGF21@Liposomes or ICG-conjugated MFVLs via their tail vein at different time points to create a time gradient of 0.5, 2, 6, 12, and 24 h. Next, the mice were euthanized and fixed in the supine position. Then, their thoracic cavity was immediately opened, and the entire aorta was rapidly isolated from the heart to the branches of the iliac artery and kept in 10% neutral-buffered formalin until needed. Fluorescence images were acquired in the NIR-II range.

Aortic cross-section confocal microscopy

The mice were administered ICG-conjugated MnO_2 NF-FGF21@Liposomes or ICG-conjugated MFVLs via their tail vein. Next, 2 h later, they were euthanized and fixed in the supine position. Then, their thoracic cavity was immediately opened, and the entire aorta was rapidly isolated from the heart to the branches of the iliac artery and kept in 10% neutral buffered formalin until needed. Next, a cryostat was used to prepare sections, which were then mounted in mounting medium containing DAPI and observed and imaged under a CSLM.

Immunohistochemical and immunofluorescence staining analysis

Aortic root cryosections were blocked using a solution containing 1% BSA and 0.1% Triton X-100 for 1 h at room temperature. Next, the sections were incubated overnight at 4°C with antibodies against CD86 (1:200) and CD206 (1:200). Then, the sections were observed and imaged under a CLSM (TCS SP8, Leica).

Hemolysis rate assay

As a blood-contacting medical device, vascular stents require thorough evaluation of their hemocompatibility. This study employed both *in vitro* platelet adhesion tests and a semi-*in vivo* blood circulation model to assess the hemocompatibility of the nanocoating. Additionally, the hemolysis test is a critical indicator for evaluating the blood compatibility of implantable devices.

The hemolysis assay used fresh whole blood drawn from healthy rabbits. First, a diluted blood solution was prepared by mixing 4 mL of fresh whole blood with 5 mL of 9% saline. Next, each test sample was immersed in 9.8 mL of 9% saline and incubated at 37°C for 30 min. The negative control group was immersed in 9.8 mL of 9% saline, while the positive control group was immersed

in 9.8 mL of distilled water; both were subjected to the same incubation conditions. Then, 0.2 mL of the diluted blood solution was added to each group and incubated at 37°C for 1 h. After incubation, all mixtures were centrifuged at 3000 rpm for 5 min. Then, the supernatant from each group was collected, and its absorbance at 540 nm was measured. Since the osmotic pressure of the saline in the negative control group is equivalent to that of whole blood, no hemolysis occurs, and its absorbance is defined as 0. In contrast, the distilled water in the positive control group has a lower osmotic pressure, causing complete hemolysis, and its absorbance is defined as 1.

Vascular stent implantation in the rabbit model

Two surgical approaches were used for the *in vivo* vascular stent implantation experiments: the external carotid artery approach and the femoral artery approach. The *in vivo* vascular stent implantation experiments used 12 healthy male New Zealand white rabbits (2.5–3.0 kg, ~3 months old) or Rag1/Il2rg double-knockout rabbits (male, 2.5–3.0 kg), which were randomly divided into three groups (n = 6/group): WE43 stent, EC-MFVL-coated stent, and WE43 bare stent (control). Before the experiment, all stents were sterilized with ethylene oxide at 37°C for 12 h. The rabbits were placed under general anesthesia using intravenous injection of pentobarbital sodium (25 mg/mL, 0.7 mL/kg) via the ear vein. Next, the common carotid artery (CCA) and its branches (internal and external carotid [ECA] arteries) were surgically exposed. Then, a balloon catheter was used to deliver the stent via the ECA into the CCA. Next, the stent was expanded and secured in place at 8 atm balloon pressure for 40 s. After catheter withdrawal, the ECA was ligated, and the rabbits received an intramuscular injection of ampicillin sodium solution (10 mg/mL, 1 mL/kg). Postoperatively, antiplatelet therapy consisting of aspirin (100 mg/kg) and clopidogrel (3 mg/kg) was administered for 3 consecutive days. For the femoral artery approach, under general anesthesia, the right femoral artery of the rabbit was surgically exposed via a longitudinal groin incision. A 4F arterial sheath was introduced into the femoral artery using the Seldinger technique, and heparin (100–150 IU/kg) was administered for anticoagulation. Under fluoroscopic guidance, a 0.014-inch guidewire and a 2.7F microcatheter were advanced through the femoral artery, abdominal aorta, and thoracic aorta into the common carotid artery. Angiography was performed to delineate the vessel diameter and target segment. A balloon-expandable stent mounted on a delivery catheter was then advanced over the guidewire to the target position and deployed by inflating the balloon at 8–12 atm. Post-deployment angiography confirmed adequate stent expansion and distal flow patency. The catheter and sheath were subsequently removed, the femoral artery was ligated, and the incision was closed. During the 1-year post-implantation period, serial carotid artery CTA scans were performed on the rabbits at regular intervals, with subsequent image analysis conducted using RadiAnt DICOM Viewer (version 2020.2.3). At predetermined time points, the rabbits were euthanized, and the stented arterial segments were explanted. The harvested stent-artery complexes were divided equally into three segments for immunofluorescence staining, immunohistochemical staining, and scanning electron microscopy (SEM) analysis. The extent of vascular healing in pathological tissue sections was analyzed by H&E staining and immunostaining with antibodies against CD206 and CD86.

Ex vivo evaluation of anti-thrombogenicity in an arteriovenous shunt assay

This experiment used three adult New Zealand white rabbits (2.5–3.0 kg, ~3 months old) obtained from Chengdu Dossy Experimental Animals Co., Ltd. Before the experiment, EGCG-Cys and EGCG-Cys-MFVL nanocoatings were prepared on polyvinyl chloride (PVC) tubes (F16, Guilong Medical Equipment Co., Ltd.), and then connected in series using aspirator cannulas (Φ 1.2 mm, Guilong Medical Equipment Co., Ltd.). Next, the rabbits were anesthetized via injection with pentobarbital sodium (25 mg/mL, 0.7 mL/kg) through the ear vein. Then, their right carotid artery and left external jugular vein were carefully separated and pierced with indwelling needles (Φ 1.3 mm, B. Braun Melsungen AG). Next, the tandem uncoated, EGCG-Cys nanocoated, and EGCG-Cys-MFVL nanocoated PVC tubes were connected to the vessels with indwelling needles to form a closed-loop circuit. After 2 h of blood circulation, the samples were quickly removed and washed carefully with PBS. The thrombus bound to the sample surfaces was collected, weighed, and imaged. The cross-section of the harvested tubes was imaged to analyze the occlusion rates of the samples.

In vitro platelet adhesion test

Blood was drawn from a healthy New Zealand white rabbit and anticoagulated with sodium citrate. Next, nanocoated PLA sheets (1 × 1 cm) were placed in 24-well cell culture plates and incubated with 500 μL of platelet-rich plasma (PRP) for 2 h at 37°C. Then, the samples were carefully washed with PBS to remove nonadherent platelets. Next, the samples were fixed with 2.5% glutaraldehyde overnight. Then, the *in vitro* blood compatibility of the nanocoatings was evaluated by observing the morphology of the adhered platelets using a scanning electron microscope (SEM).

Quantification of target lesion failure (TLF)

Target lesion failure (TLF) was defined as the occurrence of complete in-stent occlusion or severe luminal restenosis within the stented segment during follow-up, as assessed by computed tomography angiography (CTA). CTA imaging was performed at 1, 3, 6, and 12 months after stent implantation. For each time point, the stented segment and the adjacent reference vessel were reconstructed using multiplanar reformation (MPR) and cross-sectional imaging. TLF events were identified when the stented vessel exhibited complete luminal occlusion or loss of distal contrast perfusion, indicating thrombotic occlusion or severe restenosis.

The cumulative TLF incidence was calculated as

$$TLF(\%) = \frac{\text{number of vessels with TLF events}}{\text{total number of implanted vessels}} \times 100$$

Quantification of minimum lumen area (MLA)

The minimum lumen area (MLA) within the stented segment was measured from CTA cross-sectional images. For each stented artery, serial cross-sectional images along the stent axis were reconstructed using MPR. The luminal area was measured at multiple points along the stent, and the smallest cross-sectional luminal area was defined as the MLA. To minimize measurement bias, the lumen contours were semi-automatically segmented using imaging analysis software, followed by manual verification by two independent observers blinded to the experimental groups.

MLA values were expressed in square millimeters (mm^2).

Calculation of luminal area stenosis

The degree of luminal stenosis was quantified by comparing the MLA within the stented segment to the reference vessel area (RVA) obtained from the adjacent non-stented arterial segment. Luminal area stenosis was calculated as:

$$\text{Luminal Area Stenosis}\,(\%) = \left(1 - \frac{MLA}{RVA}\right) \times 100$$

Where: MLA represents the minimum lumen area within the stented segment;RVA represents the reference vessel area measured from the proximal or distal non-stented vessel segment. All measurements were performed at 1, 3, 6, and 12 months post-implantation.

Calcium deposition

After euthanasia, the hearts, aortas, and carotid arteries were carefully harvested from rabbits in the WE43 stent group and the EGCG-MFVL-WE43 stent group. The collected tissues were gently rinsed three times with pre-cooled phosphate-buffered saline (PBS, pH 7.4, 4°C) for 1 min each to remove residual blood and surface impurities. The tricuspid valve region of the heart and the vascular wall tissues were then precisely excised into small specimens (approximately 2 mm × 2 mm × 1 mm). After labeling, the samples were immediately stored in a −80°C ultra-low temperature freezer for 1–2 days to ensure complete freezing and preserve the native distribution of calcium ions. Frozen samples were subsequently transferred to a freeze dryer and dehydrated under vacuum (≤10 Pa) at −50°C for 48 h until completely dried. Care was taken to avoid mechanical compression or contamination during sample handling. The dried specimens were mounted onto conductive sample stubs using conductive adhesive and sputter-coated with a thin platinum layer using an ion sputter coater (15 mA, 60 s, coating thickness 5–10 nm) to improve surface conductivity.

The samples were then examined using a scanning electron microscopy–energy dispersive spectroscopy (SEM-EDS) system under the following conditions: accelerating voltage, 15 kV; working distance, 8 mm; magnification, 500×. For each specimen, three representative regions of interest were selected for analysis. EDS acquisition was performed for each selected region with a collection time of 60 s and a count rate of 1000–2000 cps. Calcium and magnesium were specifically analyzed. After background correction, each region was measured three times, and the weight percentage (wt %) and atomic percentage (at%) of the detected elements were recorded. Elemental data were processed using the accompanying EDS analysis software, and calcium distribution mapping images were generated to characterize the spatial distribution of calcium deposition. To minimize instrumental error, three blank control measurements were included for calibration. Each experimental group contained three biological samples, and all procedures were performed under sterile and low-temperature conditions. The experiment was independently repeated three times, and the mean values were used for analysis.

Stent implantation in the porcine model

The experimental male miniature potbellied pigs (~35 kg, ~6 months old) were quarantined for 8 days prior to the trials to confirm that they were free from infectious diseases. Next, 12 h before stent implantation, they were administered oral antiplatelet pretreatment with aspirin (75 mg/kg) and clopidogrel (100 mg/kg). Then, under sterile conditions, systemic anticoagulation was achieved via auricular vein injection of heparin (200 IU/kg), followed by puncture of the right femoral artery to establish vascular access. After contrast agent injection (200 μg/animal) for coronary angiography (CTA)-guided positioning, WE43 and EGCG-Cys-MFVL-WE43 nanocoated stents were randomly implanted into major coronary branches (right coronary artery and left anterior descending artery) through a 6F guiding catheter over a 0.014-inch guidewire. Then, the stents were expanded using high-pressure balloon inflation at 12 atm for 30 s to ensure proper apposition, followed by repeat CTA to assess stent patency and positioning. Postoperatively, the pigs were administered intramuscular penicillin (20,000 units/mL, 1 mL/kg) daily for 7 days along with continued oral antiplatelet therapy. At 1 and 3 months post-implantation, vascular patency was evaluated using optical coherence tomography, with images analyzed using

RadiAnt DICOM Viewer (version 2020.2.3). Stented arterial segments (n = 3 animals/group) were harvested for histological analysis (H&E staining), after which the animals were euthanized using a pentobarbital sodium overdose.

Safety evaluation

The safety of the nanoparticles and nanocoatings was investigated in 12 healthy female mice randomly divided into four groups (n = 3/group). Whole blood was collected from the orbit at 0, 12, 72, and 168 h post-catalytic hydrogel injection, and the routine blood indices were determined: white blood cell count (WBC), red blood cell count (RBC), platelet count (PLT), hemoglobin (HGB), mean corpuscular volume (MCV), mean corpuscular hemoglobin (MCH), MCH concentration (MCHC), hematocrit (HCT), monocyte percentage (MON%), eosinophil percentage (EOS%), neutrophil percentage (NEU%), and lymphocyte percentage (LYM%).

At different time points (1, 3, 6, 9, and 12 months), mice treated with MnO_2NFS-FGF21 or MnO_2NFS-FGF21@VHPK-Lipo (injection dose: 0.12 mg/kg, once per month) were anesthetized and fixed on an operating platform. The thumb and index finger of the left hand were used to gently compress both sides of the neck to induce ocular congestion and protrusion. Using ophthalmic forceps held in the right hand, the eyeball was rapidly grasped and gently pulled outward to enucleate the eye, and blood was collected using an anticoagulant tube.

After blood collection, sterile cotton was immediately applied to the orbital cavity to achieve hemostasis, and the mice were subsequently euthanized. The collected blood samples were subjected to biochemical and immunological analyses, including cardiac function markers (BNP), liver function indicators (AST, ALT), renal function marker (BUN), and complement activation markers (C3a, C5a, and sC5b-9).

QUANTIFICATION AND STATISTICAL ANALYSIS

Parallel experiments were designed for all blood, cell, *ex vivo*, and *in vivo* assays, and were repeated three times. The data are presented as the mean ± standard deviation (SD), with detailed information on sample size (n) and experimental conditions provided in the corresponding figure legends. The data were statistically analyzed using SPSS (version 11.5, IBM Corp.). Comparisons between two groups were conducted using unpaired two-tailed Student's t test. For multiple group comparisons, one-way or two-way analysis of variance (ANOVA) followed by Tukey's multiple comparisons test was applied. Survival data were analyzed using the log rank test. A p value < 0.05 was considered statistically significant. Exact p values are provided in the figures, with significance levels denoted as follows: *, $p < 0.05$; **, $p < 0.01$; ***, $p < 0.001$; ****, $p < 0.0001$. All statistical analyses were performed in GraphPad Prism 9.0.

Cell Reports Medicine, Volume *7*

Supplemental information

Microenvironment-responsive magnesium stents for targeted atherosclerotic plaque removal and restenosis mitigation

Lin Shen, Yi Zhong, Yanran Bi, Ziyu Pang, Youyu Wei, Zhangyu Yang, Xingru Ding, Chengli Jiang, Junchao Yu, Zijian Fang, Mengqian Zhai, Jinhong Sun, Junyan Li, Jieli Zhu, Zhongwei Zhao, Gaofeng Shu, Chenying Lu, Lingchun Lv, Jingjing Song, Minjiang Chen, Xiaokun Li, Zhouguang Wang, Weiqian Chen, and Jiansong Ji

Supplementary Information

Supplemental Figures

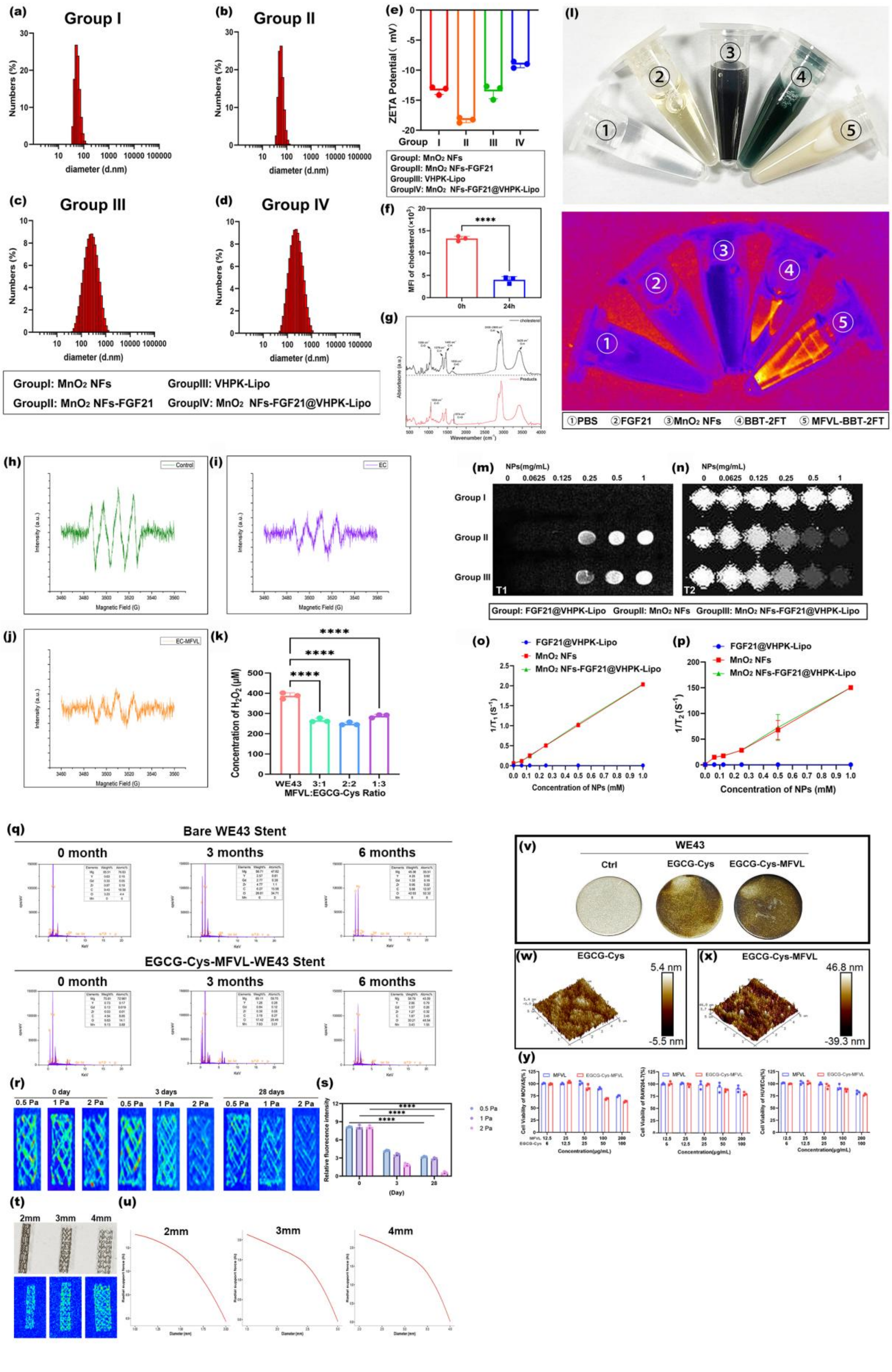

Fig. S1.Characterization of nanoparticle formulations, ROS scavenging capacity, MRI contrast properties, stent coating performance, and cytocompatibility (related to Fig. 1). (a) Group I: MnO_2

NFs, **(b)** Group II: MnO_2 NF-FGF21, **(c)** Group III: VHPK-Lipo, and **(d)** Group IV: MFVL. **(e)** Zeta potential measurements of each nanoparticle formulation. Group I: MnO_2 NFs, Group II: MnO_2 NF-FGF21, Group III: VHPK-Lipo, and Group IV: MFVL. **(f)** Quantitative bar graph analysis corresponding to Fig. 1b. **(g)** FTIR spectra of cholesterol (black) and its enzymatic oxidation products (red). **(h-j)** ESR spectra of different sample groups—(h) Ctrl, (i) EC-coated, and (j) EGCG-Cys-MFVL-coated substrates—used to evaluate the scavenging capacity of ROS, including •OH and/or superoxide radical ($O_2•^-$). **(k)** Determination of H_2O_2 scavenging efficiency of nanocoatings with varying MFVL:EGCG-Cys mass ratios (3:1, 2:2, and 1:3) using the HRP-catalyzed ABTS assay. **(l)** Visible light (top) and NIR-II fluorescence (bottom) images of PBS, FGF21, MnO_2 NFs, BBT-2FT, and MFVL-BBT-2FT. **(m, n)** T_1-weighted (m) and T_2-weighted (n) MRI images of FGF21@VHPK-Lipo (Group I), MnO_2 NFs (Group II), and MFVL (Group III) at varying concentrations (0~1 mg/mL). **(o, p)** Plots of $1/T_1$ (o) and $1/T_2$ (p) relaxation rates as a function of nanoparticle concentration for each group. **(q)** Time-dependent EDS spectra of bare and EGCG-Cys-MFVL coated WE43 stents during degradation (0, 3, and 6 months). **(r)** Fluorescence imaging of EGCG-Cys-MFVL coated stents under different shear stress conditions (0.5, 1, and 2 Pa) at indicated time points (0, 3, and 28 days). **(s)** Quantification of fluorescence intensity under different shear stress conditions corresponding to (r). **(t)** Representative optical images and corresponding fluorescence distribution maps of EGCG-Cys-MFVL coated WE43 stents with different diameters (2, 3, and 4 mm). **(u)** Radial force–diameter curves of EGCG-Cys-MFVL coated WE43 stents with different diameters (2, 3, and 4 mm). **(v)** Images of WE43 alloy discs with different surface modifications: uncoated (Ctrl) and coated with EGCG-Cys or EGCG-Cys-MFVL. **(w, x)** AFM images of EC (w) and EGCG-Cys-MFVL (x) nanocoatings, showing differences in nanoscale surface roughness. **(y)** Cell viability of MOVAS VSMCs, RAW264.7 macrophages, and HUVECs after incubation with varying concentrations of MFVL and EGCG-Cys-MFVL formulations, evaluated using the CCK-8 assay to assess cytocompatibility. Data are shown as mean ± SD. Statistical significance was calculated by one-way ANOVA with Tukey's test. Significance: *, $P < 0.05$; **, $P < 0.01$; ***, $P < 0.001$; ****, $P < 0.0001$.

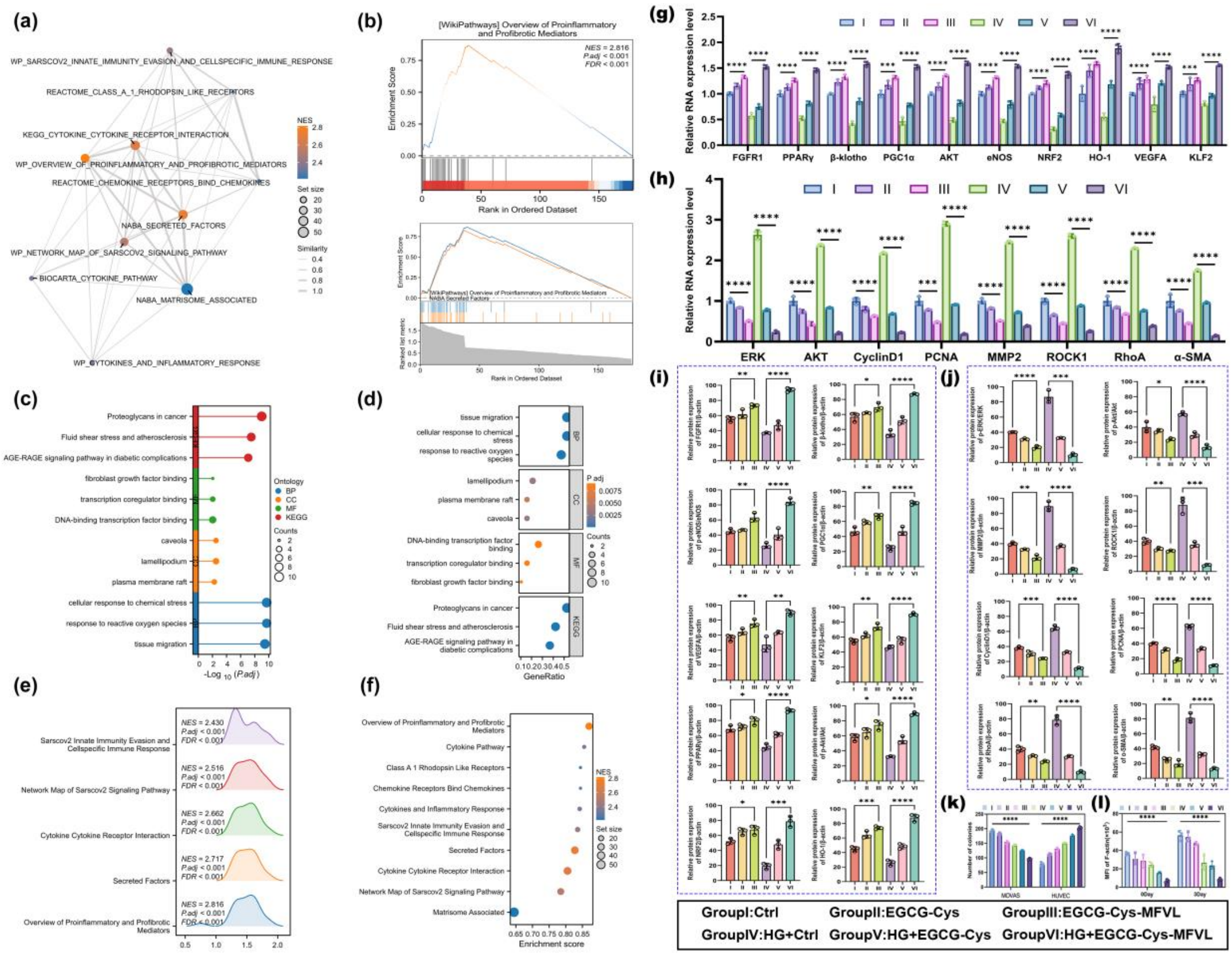

Fig. S2.Transcriptomic pathway enrichment analysis and validation of endothelial and smooth muscle cell functional gene expression under ISR-like pathological conditions (related to Fig. 2). **(a)** Network visualization of enriched pathways based on transcriptomic profiling, highlighting interactions among cytokine signaling, chemokine activity, and extracellular matrix–related processes. **(b)** Gene set enrichment analysis (GSEA) plots of representative pathways associated with proinflammatory and profibrotic mediators. **(c)** Functional enrichment analysis of differentially expressed genes based on Gene Ontology (GO) and KEGG pathways. **(d)** Bubble plot showing enriched GO terms (BP, CC, MF) and KEGG pathways ranked by gene ratio and adjusted *P* value. **(e)** GSEA enrichment plots of selected pathways involved in immune regulation and inflammatory signaling. **(f)** Summary of enriched pathways with corresponding normalized enrichment scores (NES) and gene set sizes. The data were derived from transcriptomic profiling under ISR-like pathological conditions. Statistical significance was determined based on adjusted *P* value and false discovery rate (FDR). **(g)** Relative mRNA expression levels of endothelial function–related genes, including FGFR1, PPARγ, β-klotho, PGC1α, AKT, eNOS, NRF2, HO-1, VEGFA, and KLF2. **(h)** Relative mRNA expression levels of genes associated with smooth muscle cell proliferation and migration, including ERK, AKT, Cyclin D1, PCNA, MMP2, ROCK1, RhoA, and α-SMA. **(i)** Quantification of Western blot analysis corresponding to endothelial function–related signaling pathways in HUVECs shown in Fig. 2e. **(j)** Quantification of Western blot analysis corresponding to proliferation- and migration-related signaling pathways in MOVAS cells shown in Fig. 2d. **(k)** Quantification of colony formation in MOVAS and HUVECs. **(l)** Quantification of F-actin fluorescence intensity at indicated time points.Group I: Ctrl; Group II: EGCG-Cys; Group III: EGCG-Cys-MFVL; Group IV: Ctrl + HG; Group V: EGCG-Cys + HG; Group VI: EGCG-Cys-MFVL + HG. HG, high-glucose condition mimicking a pathological ISR-like microenvironment. Data are shown as mean ± SD. Statistical significance was calculated by one-way ANOVA with Tukey's test. Significance: *, $P < 0.05$; **, $P <$

0.01; ***, $P < 0.001$; ****, $P < 0.0001$.

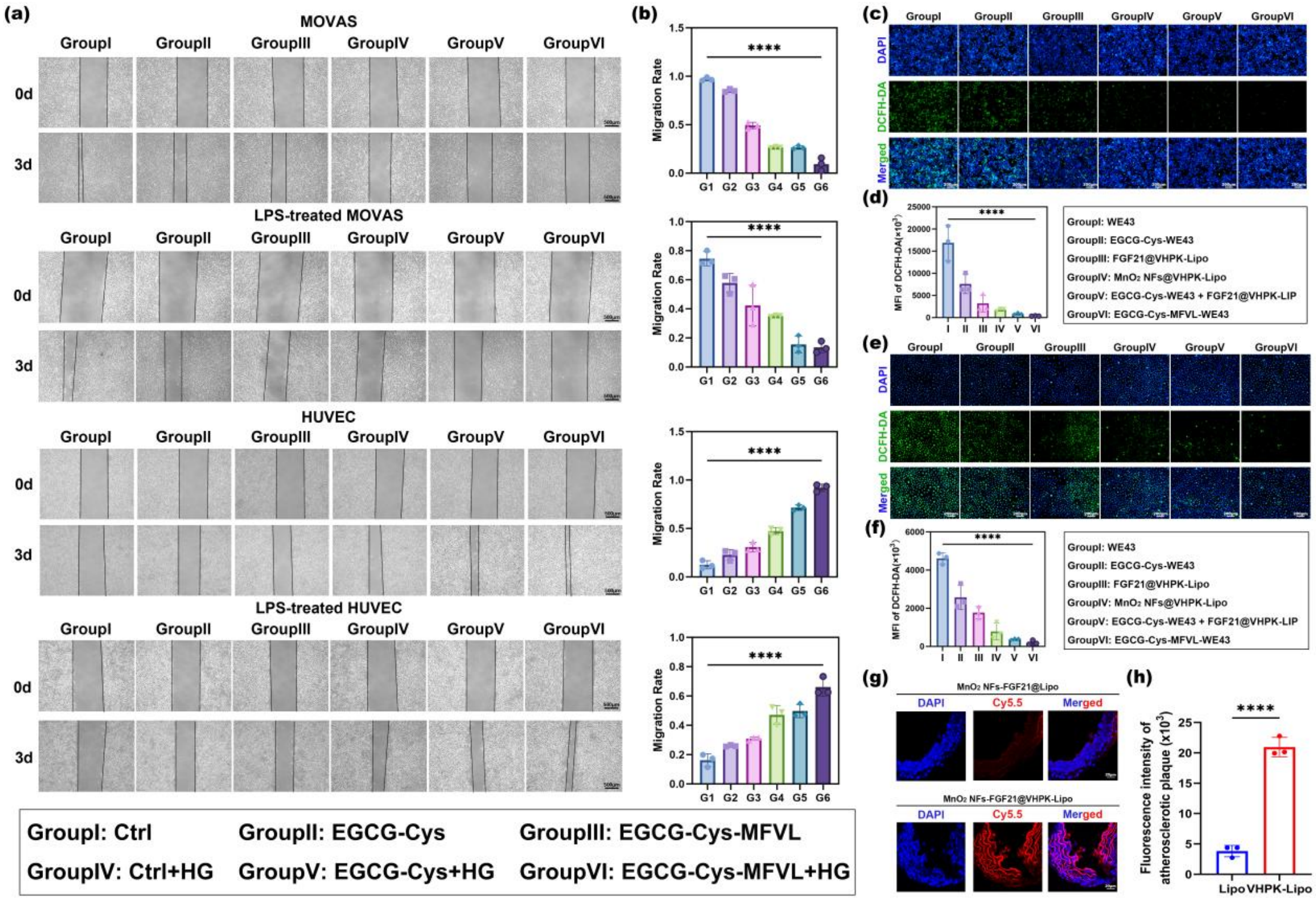

Fig. S3.Evaluation of cell migration, intracellular ROS scavenging in foam cells, and plaque targeting of nanoparticle formulations under inflammatory and metabolic stress conditions (related to Fig. 2-4). (a) Wound healing assay of MOVAS cells and HUVECs under normal and LPS-treated conditions across different treatment groups at 0 and 3 days. Scale bars, 500 μm. **(b)** Quantification of migration rates corresponding to panel (a). Group I: Ctrl; Group II: EGCG-Cys; Group III: EGCG-Cys-MFVL; Group IV: Ctrl + HG; Group V: EGCG-Cys + HG; Group VI: EGCG-Cys-MFVL + HG. LPS, lipopolysaccharide used to induce an inflammatory ISR-like microenvironment; HG, high-glucose condition mimicking metabolic pathological conditions. **(c)** Representative fluorescence images of intracellular ROS levels detected by DCFH-DA staining in macrophage-derived foam cells across different treatment groups. Nuclei are stained with DAPI (blue), and ROS signals are shown in green. Scale bars, 200 μm. **(d)** Quantification of mean fluorescence intensity (MFI) of DCFH-DA corresponding to panel (c). Group I: WE43; Group II: EGCG-Cys-WE43; Group III: FGF21@VHPK-Lipo; Group IV: MnO_2 NFs@VHPK-Lipo; Group V: EGCG-Cys-WE43 + FGF21@VHPK-Lipo; Group VI: EGCG-Cys-MFVL-WE43. **(e)** Representative fluorescence images of intracellular ROS levels detected by DCFH-DA staining in smooth muscle cell–derived foam cells across different treatment groups. Nuclei are stained with DAPI (blue), and ROS signals are shown in green. Scale bars, 200 μm. **(f)** Quantification of mean fluorescence intensity (MFI) of DCFH-DA corresponding to panel (e). Group I: WE43; Group II: EGCG-Cys-WE43; Group III: FGF21@VHPK-Lipo; Group IV: MnO_2 NFs@VHPK-Lipo; Group V: EGCG-Cys-WE43 + FGF21@VHPK-Lipo; Group VI: EGCG-Cys-MFVL-WE43. **(g)** Representative fluorescence microscopy images of atherosclerotic plaques from each group showing DAPI (blue) and Cy5.5-labeled Lipo or MFVL (red) signals. Scale bars, 20 μm. **(h)** Quantitative analysis of Cy5.5 fluorescence intensity in plaques from Lipo and MFVL groups. Data are shown as mean ± SD.

Statistical significance was calculated by one-way ANOVA with Tukey's test. Significance: *, $P < 0.05$; **, $P < 0.01$; ***, $P < 0.001$; ****, $P < 0.0001$.

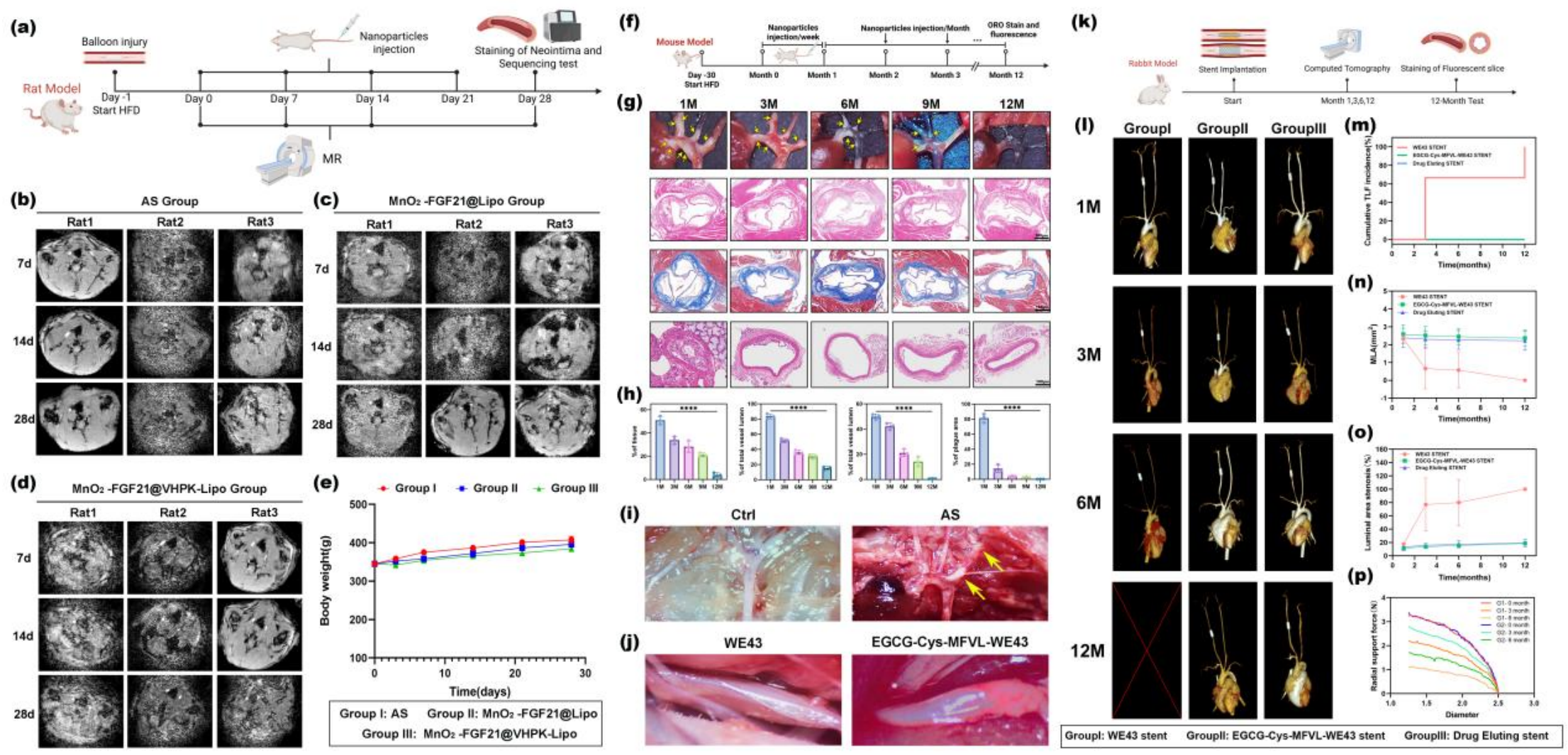

Fig. S4.Longitudinal *in vivo* evaluation of nanoparticle treatment and stent performance in rat, mouse, and rabbit models of atherosclerosis and vascular injury (related to Fig. 5, 6). (a) Schematic timeline of the rat model experiment. Following balloon-induced vascular injury and initiation of a high-fat diet, the rats received nanoparticle treatment on day 7, followed by serial MRI scans at days 7, 14, and 28. Neointimal tissue was harvested on day 28 for histological staining and sequencing analysis. **(b-d)** Representative MRI images of aortic roots from rats in Group I (AS), Group II (MnO_2-FGF21@Lipo), and Group III (MFVL) at 7, 14, and 28 days post-treatment. **(e)** Body weight changes of rats over 28 days in all groups. **(f)** Schematic illustration of the experimental design for long-term treatment in *Apoe*$^{-/-}$ mice, including high-fat diet (HFD) induction and periodic nanoparticle administration. **(g)** Representative stereomicroscopic images of aortic roots at different time points (1, 3, 6, 9, and 12 months) following MFVL treatment. Yellow arrows indicate atherosclerotic lesions. Hematoxylin and eosin (H&E) staining of aortic root sections at indicated time points. Masson's trichrome staining of aortic root sections for evaluation of collagen deposition and plaque remodeling. Histological staining of vascular sections showing morphological changes during long-term treatment. Scale bars, 100 μm. **(h)** Quantitative analysis of plaque burden, including percentage of lesion area, vessel lumen area, and plaque area at different time points. **(i)** Stereomicroscopic observation of aortic arches in the Ctrl (C57BL/6J) and AS (*Apoe*$^{-/-}$ + high-fat diet) mouse models. **(j)** Stereomicroscopic images of carotid arteries in AS rabbits implanted with WE43 or EGCG-Cys-MFVL-WE43 stents. **(k)** Schematic illustration of the experimental design for long-term evaluation in a rabbit stenting model, including stent implantation, serial computed tomography (CT) imaging, and histological analysis up to 12 months. **(l)** Representative three-dimensional CT reconstructions of stented vessels at 1, 3, 6, and 12 months post-implantation in Group I (bare WE43 stent), Group II (EGCG-Cys-MFVL-WE43 stent), and Group III (drug-eluting stent). **(m)** Cumulative target lesion failure (TLF) incidence over time across different treatment groups. **(n)** Longitudinal quantification of minimal lumen area (MLA) derived from CT imaging. **(o)** Luminal area stenosis (%) over time in different groups. **(p)** Radial support force–diameter curves of stents at different time points, reflecting the evolution of mechanical integrity during degradation. Data are shown as mean ± SD. Statistical significance was calculated by

one-way ANOVA with Tukey's test. Significance: *, $P < 0.05$; **, $P < 0.01$; ***, $P < 0.001$; ****, $P < 0.0001$.

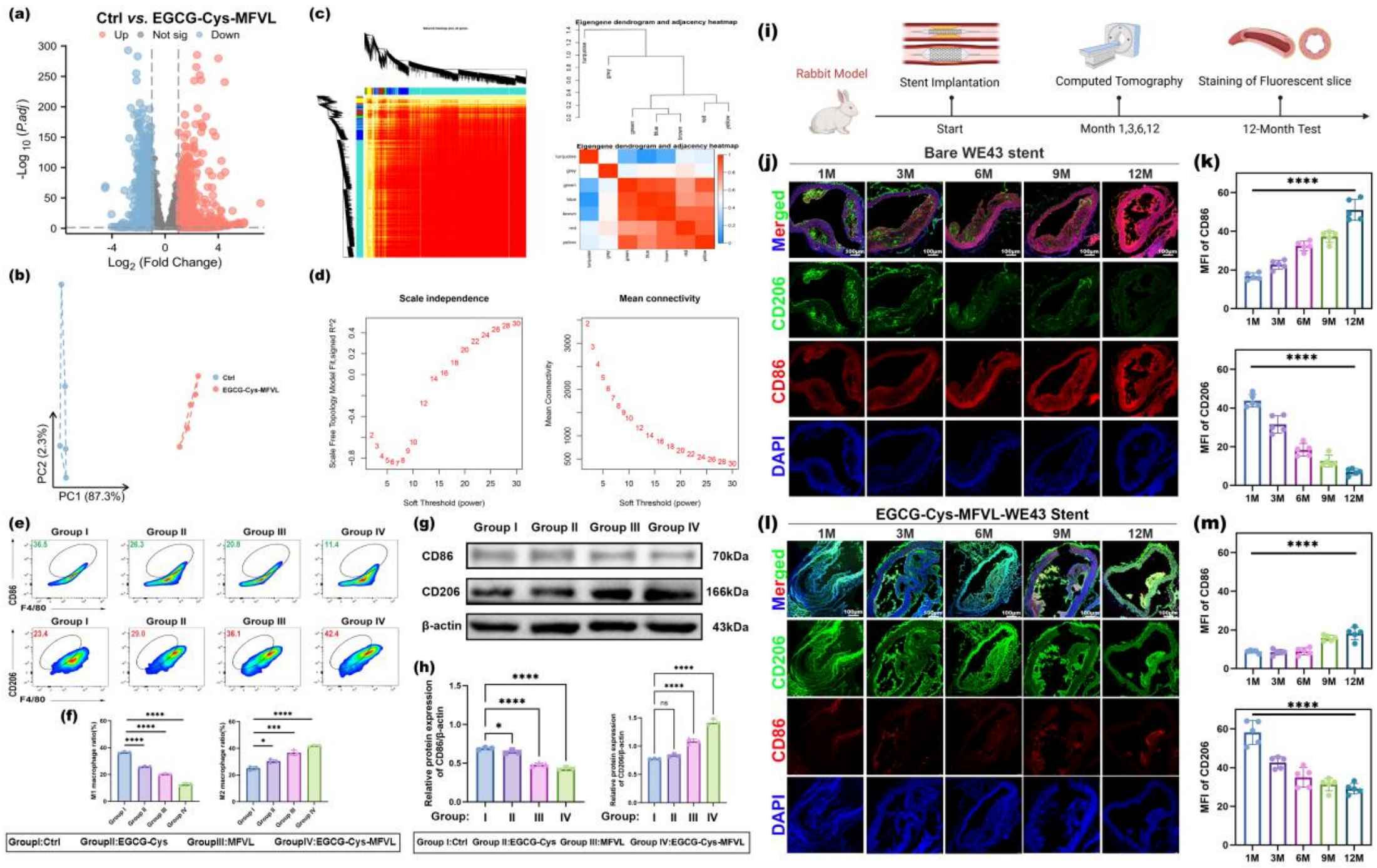

Fig. S5.Transcriptomic profiling, WGCNA, and long-term macrophage polarization analysis following EGCG-Cys-MFVL-coated stent implantation (related to Fig. 7). (a) Volcano plot of genes differentially expressed between EGCG-Cys-MFVL-coated stents and bare WE43 stents, generated based on $\log_2$ fold change ($\log_2$FC) and adjusted *p*-values. Red and blue dots represent genes with significant upregulation and downregulation, respectively ($P < 0.05$, $|\log_2FC| > 1$). **(b)** Principal component analysis (PCA) of transcriptomic profiles from the Ctrl and EGCG-Cys-MFVL groups, with dimensionality reduction plotted across the first two principal components. **(c)** WGCNA of transcriptomic data obtained from rat carotid arteries 1 month after implantation of EGCG-Cys-MFVL-coated and bare WE43 stents. A topological overlap matrix (TOM) was constructed, and gene modules were identified using hierarchical clustering with dynamic tree cutting. Module eigengene relationships were visualized through dendrograms and adjacency heatmaps. **(d)** Soft-thresholding power (β) was determined based on the scale-free topology criterion. The relationship between soft threshold and scale independence (left) and mean connectivity (right) was assessed to ensure optimal network construction. Flow cytometry analysis of aortic single-cell suspensions was performed 1 month after stent implantation to assess macrophage polarization. **(e)** Cells were stained with F4/80 and CD86 (M1) or CD206 (M2) antibodies. Representative density plots are shown for CD86 and CD206. **(f)** Quantification of M1 and M2 macrophage ratios. Group I: bare stents (Ctrl), Group II: EC-coated stents, Group III: MFVL-coated stents, and Group IV: EGCG-Cys-MFVL-coated stents. **(g)** Representative immunoblots. **(h)** Quantitative analysis of relative protein expression for CD86 and CD206. Group I: bare stents (Ctrl), Group II: EC-coated stents, Group III: MFVL-coated stents, and Group IV: EGCG-Cys-MFVL-coated stents. **(i)** Schematic illustration of the long-term experimental design, including stent implantation and serial histological assessment at 1, 3, 6, 9, and 12 months. **(j)** Representative immunofluorescence staining of aortic sections from rabbits implanted with bare WE43 stents, showing CD206$^+$ (M2 macrophages, green), CD86$^+$ (M1

macrophages, red), and nuclei (DAPI, blue) at indicated time points. Scale bars, 100 μm. **(k)** Quantitative analysis of mean fluorescence intensity (MFI) of CD86^{+} and CD206^{+} in the bare WE43 group over time. **(l)** Representative immunofluorescence staining of aortic sections from rabbits implanted with EGCG-Cys-MFVL-WE43 stents at corresponding time points. Scale bars, 100 μm. **(m)** Quantitative analysis of MFI of CD86^{+} and CD206^{+} in the EGCG-Cys-MFVL-WE43 group. Data are shown as mean ± SD. Statistical significance was calculated by one-way ANOVA with Tukey's test. Significance: *, $P < 0.05$; **, $P < 0.01$; ***, $P < 0.001$; ****, $P < 0.0001$.

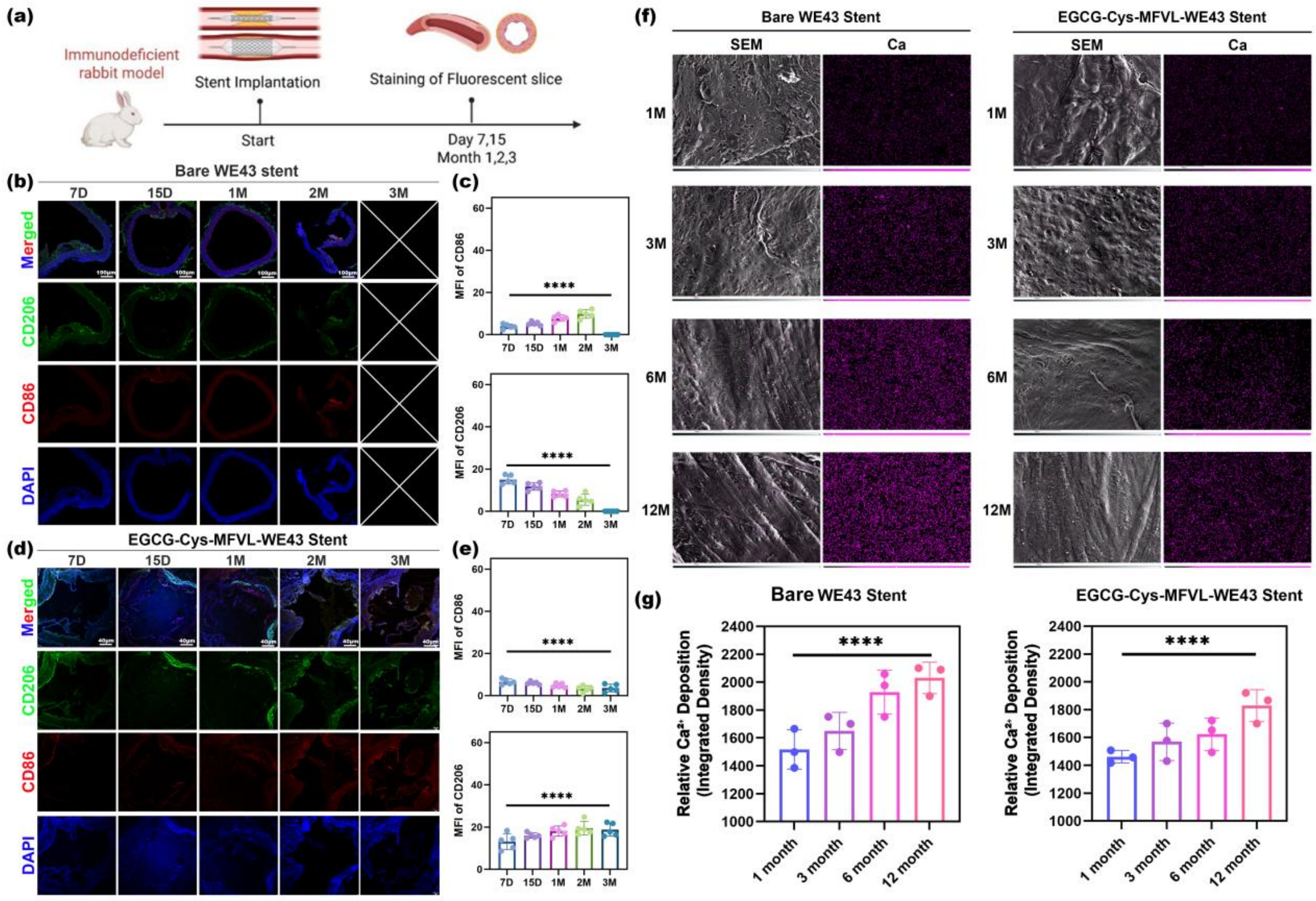

Fig. S6.Time-dependent macrophage polarization and peri-stent calcium deposition analysis in immunodeficient rabbits following bare and EGCG-Cys-MFVL-coated WE43 stent implantation (related to Fig. 7). (a) Schematic illustration of the experimental design in immunodeficient rabbits, including stent implantation and serial histological analysis at 7 days, 15 days, and 1–3 months. **(b)** Representative immunofluorescence staining of vascular sections from rabbits implanted with bare WE43 stents, showing CD206^{+} (green), CD86^{+} (red), and nuclei (DAPI, blue) at indicated time points. Scale bars, 100 μm. **(c)** Quantitative analysis of MFI of CD86 and CD206 in the bare WE43 group. **(d)** Representative immunofluorescence staining of vascular sections from rabbits implanted with EGCG-Cys-MFVL-WE43 stents. Scale bars, 100 μm. **(e)** Quantitative analysis of MFI of CD86^{+} and CD206^{+} in the EGCG-Cys-MFVL-WE43 group. **(f)** Representative scanning electron microscopy (SEM) images and corresponding Ca elemental mapping of peri-stent vascular surfaces adjacent to bare WE43 and EGCG-Cys-MFVL-coated WE43 stents at indicated time points (1, 3, 6, and 12 months) after implantation. **(g)** Quantitative analysis of Ca^{2+} deposition based on integrated Ca signal intensity corresponding to (f). Data are shown as mean ± SD. Statistical significance was calculated by one-way ANOVA with Tukey's test. Significance: *, $P < 0.05$; **, $P < 0.01$; ***, $P < 0.001$; ****, $P < 0.0001$.

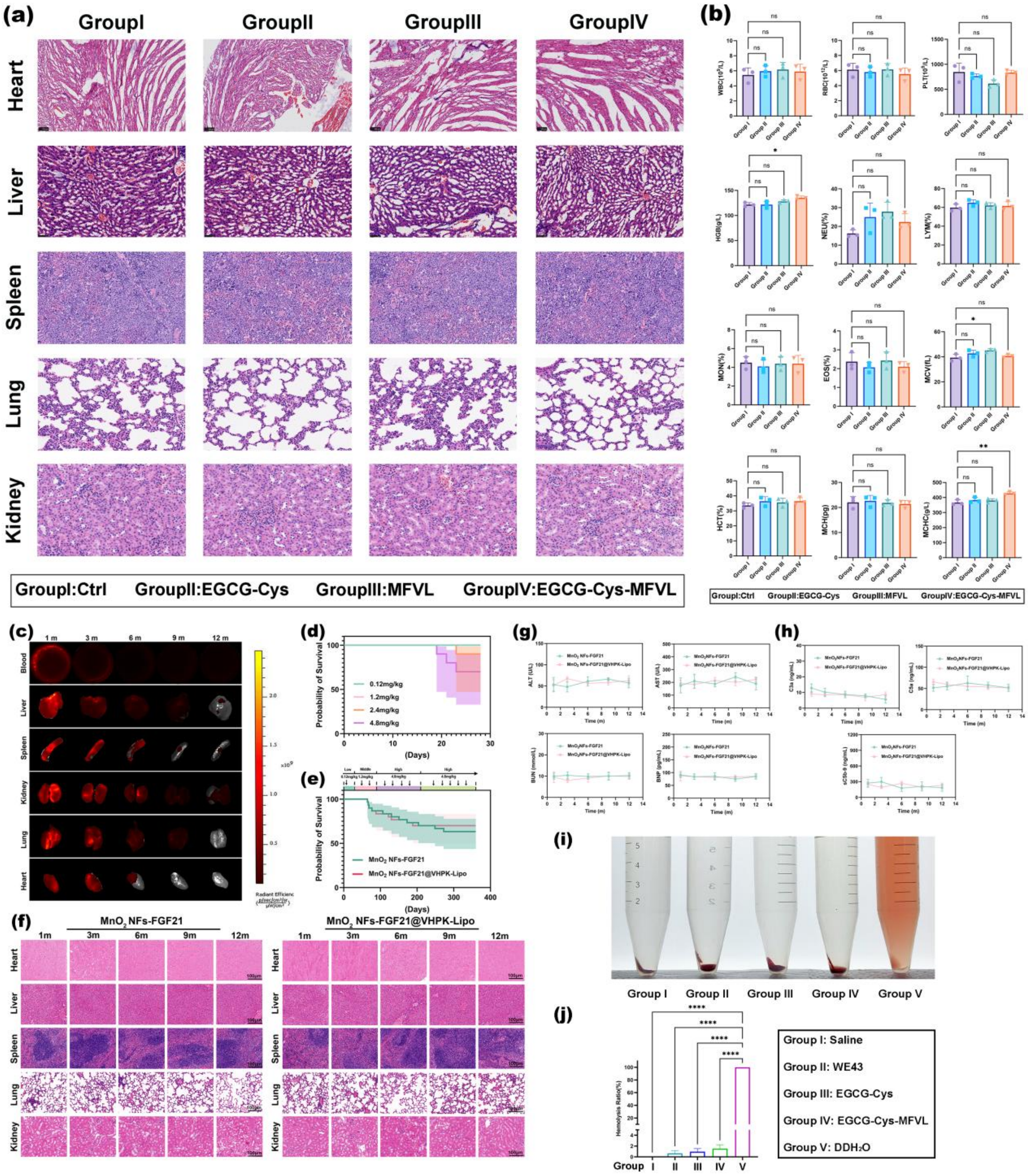

Fig. S7.*In vivo* biocompatibility, biodistribution, long-term safety, and hemocompatibility assessment of nanoparticle formulations and coated stents across multiple organ systems and hematological parameters (related to Fig. 7). (a) Major organs, including the heart, liver, spleen, lung, and kidney, were stained with H&E 1 month after stent implantation to evaluate systemic biocompatibility and potential off-target toxicity. Group I: bare stents (Ctrl), Group II: EC-coated stents, Group III: MFVL-coated stents, and Group IV: EGCG-Cys-MFVL-coated stents. Scale bars, 100 µm. **(b)** Comprehensive hematological analysis was performed 1 month after stent implantation to evaluate systemic hematotoxicity: WBC, RBC, PLT, hemoglobin, NEU%, LYM%, MON%, EOS%, MCV, HCT, MCH, and MCHC. Group I: bare stents (Ctrl), Group II: EC-coated stents, Group III: MFVL-coated stents, and Group IV: EGCG-Cys-MFVL-coated stents. **(c)** *Ex vivo* near-infrared I (NIR-I) fluorescence imaging was performed to assess the tissue distribution of Cy5.5-labeled MnO_2 NFs-FGF21 and MnO_2 NFs-FGF21@VHPK-Liposomes (0.12 mg/kg, monthly). At 1, 3, 6, 9, and 12 months post-administration, blood and major organs (heart, liver, spleen, lung, and kidney) were collected and

imaged under identical acquisition settings. Radiant efficiency was recorded for quantitative analysis. **(d)** Kaplan–Meier survival curves of mice intravenously injected with MnO_2 NFs-FGF21@VHPK-Liposomes at 0.12, 1.2, 2.4, or 4.8 mg/kg and monitored for 1 month. **(e)** Long-term survival assessment of mice treated with MnO_2 NFs-FGF21 or MnO_2 NFs-FGF21@VHPK-Liposomes under a staged monthly dosing regimen: 0.12 mg/kg (month 1), 1.2 mg/kg (months 2–3), and 4.8 mg/kg (months 4–12). All formulations were administered via tail vein injection once per month. Survival status was recorded throughout the study. **(f)** Hematoxylin and eosin (H&E) staining of heart, liver, spleen, lung, and kidney tissues collected at 1, 3, 6, 9, and 12 months after intravenous administration of MnO_2 NFs-FGF21 or MnO_2 NFs-FGF21@VHPK-Liposomes. Tissue sections were prepared and analyzed under identical histological conditions to assess long-term morphological alterations. Scale bars, 100 μm. **(g)** Mice received intravenous MnO_2 NFs-FGF21 or MnO_2 NFs-FGF21@VHPK-Liposomes (0.12 mg/kg, once monthly). Blood samples were collected at 1, 3, 6, 9, and 12 months for serum biochemical assessment, including alanine aminotransferase (ALT), aspartate aminotransferase (AST), blood urea nitrogen (BUN), and brain natriuretic peptide (BNP). **(h)** Under the same dosing regimen (0.12 mg/kg, once monthly), serum samples collected at 1, 3, 6, 9, and 12 months were analyzed for complement activation markers, including C3a, C5a, and soluble C5b-9 (sC5b-9). **(i)** Representative images of the hemolysis assay performed by incubating red blood cells with various test groups: Group I (physiological saline, negative control), Group II (WE43 alloy), Group III (EGCG-Cys nanocoating), Group IV (EGCG-Cys-MFVL nanocoating), and Group V (deionized water, positive control). **(j)** Quantification of hemolysis ratio (%) by spectrophotometric analysis of hemoglobin release from the supernatant, used to evaluate the hemocompatibility of the tested materials. Data are shown as mean ± SD. Statistical significance was calculated by one-way ANOVA with Tukey's test. Significance: *, $P < 0.05$; **, $P < 0.01$; ***, $P < 0.001$; ****, $P < 0.0001$; ns, not significant.
